# Network science reveals the structure and lasting impact of the Roman road system

**DOI:** 10.1038/s41467-026-75453-3

**Published:** 2026-09-15

**Authors:** Adam Pažout, Tom Brughmans, Pau de Soto, Matteo Mazzamurro, María Coto-Sarmiento, Clara Filet, Eduardo Herrera Malatesta, Magnus L. Nielsen, Gustav Emil Ølgaard, Peter B. Vahlstrup

**Affiliations:** 1 Department of History and Classical Studies, Aarhus University, Aarhus C, Denmark; 2 Social Resilience Lab, Aarhus University, Aarhus C, Denmark; 3 Centre for Urban Network Evolutions (UrbNet), Aarhus University, Højbjerg, Denmark; 4 Grup de Recerca en Arqueologia Clàssica (GRAC_UAB), Universitat Autònoma de Barcelona, Barcelona, Spain; 5 School of Physical and Chemical Sciences, Queen Mary University of London, London, UK; 6 Department of Archaeology and Anthropology, HUMANE, Human Ecology and Archaeology Research Group, Milà i Fontanals Institution for Research and Humanities, IMF-CSIC, Barcelona, Spain; 7 Institut Ausonius UMR 5607, University of Bordeaux-Montaigne, Pessac, France; 8 Faculty of Archaeology, Leiden University, Leiden, The Netherlands; 9 Center for Humanities Computing, Aarhus University, Aarhus C, Denmark

**Keywords:** Archaeology, History

## Abstract

Ancient infrastructure can leave lasting imprints on modern settlement patterns and mobility. The Roman road network connected vast regions of Europe, North Africa, and the Middle East, and is often mentioned as an example of such long-term impact. Yet its structural characteristics remain largely unquantified. Here we present a high spatial resolution quantitative exploratory analysis of the Roman Empire’s road system, enabled by our creation of a spatially detailed Empire-wide dataset. We address long-standing questions concerning the straightness and persistence of these roads, and the centrality of cities like Rome. We show that Roman roads tend to be straight where topography allows but less so than modern roads; provincial capitals were more centrally located than other cities for mediating terrestrial mobility; segments hypothesised to be main roads were more important intermediaries than secondary roads; and Roman road density correlates with major modern roads.

## Abstract (web summary)

A spatial analysis of nearly 300,000 km of Roman roads reveals they favoured straight routes when topography allowed, established provincial capitals as key transport hubs, and Roman roads correlate with major modern roads.

## Introduction

The Roman Empire was established in the late first century BCE, and existed as an integrated political unit until the fifth century CE in the west and in the east until the 15th century CE. At its height in 117 CE it covered an area of almost 5,500,000 km^2^ around the Mediterranean Sea, occupying much of Europe, the Middle East and North Africa. These regions were united for the first time into a single geopolitical unit which enabled mass mobility on a continental scale via maritime, riverine and terrestrial means. The Romans used and extended pre-existing roads. Terrestrial routes of some sort already connected all landlocked places where humans lived and there was a significant degree of continuity of their occupation, and vast areas were already densely urbanised with well-established road infrastructure by the time the Romans incorporated them. Importantly, however, the nature and degree of formalisation of these pre-Roman terrestrial routes varied markedly across the Empire’s later extent. In the eastern Mediterranean, where complex political organisation and urban development long predated Roman expansion, Roman authorities often encountered established long-distance networks of engineered roads linking cities and major economic and administrative centres^1^. In contrast, in many parts of the western Mediterranean, pre-Roman routes appear to have consisted largely of tracks and pathways with limited built long-distance infrastructure. Consequently, the role of Roman surveyors and engineers in creating terrestrial routes varied across the Empire, depending on those regional conditions, the density and organisation of pre-Roman communities, and the politico-military context. In some areas their work primarily consisted of upgrading and integrating existing corridors, whereas in others it required the planning and construction of new roads to connect administrative centres, military sites, and newly incorporated territories.

Significant investments were made into terrestrial road infrastructure during the Roman period, in part to support its military conquest and pacification of new regions between the 3rd century BCE and 3rd century CE, but equally to move goods and people between countryside, cities and ports. The gromatics and engineers who designed the road layouts normally chose those routes that were favourable, avoiding steep slopes and promoting roads with conditions enabling wheeled transport^2,3^. Pliny the Elder argued that Roman engineering distinguished itself from that of earlier civilisations by its practical utility, unlike, for example, ‘the pyramids, so many idle and frivolous pieces of ostentation of their resources’ (*Plin. NH* 36.75). Those engineering qualities were also identified by Vitruvius (*Vitr*. De arch. 1.3.2), ‘durability, convenience, and beauty’ became so embedded in the imperial landscape that parts of this infrastructure appear to have persisted in place for millennia.

Roman roads represent a medium that structured the flow of people, animals, disease, goods and ideas^4,5^. A detailed understanding of their structure is key to studies of major phenomena in this continental scale region, including the spread and impact of past epidemics, trade in foodstuffs and food security, the spread of technological innovations and beliefs, mass migration, and the millennia-long development of terrestrial transport. But the overall structuring principles of Roman roads are the subject of ongoing debate: their relationship to pre-Roman roads^6^, the preference for optimal paths through the landscape^7^, the degree to which a Roman interventionist government planned an efficient empire-wide road network from a top-down perspective^8,9^, and the role of local and provincial needs in road development^10^.

A precise quantitative description of the structure of Roman roads across the entire empire remains absent, despite Roman roads having been the subject of scholarly debate for over a century^11,12^. Our current understanding of its structure is largely based on qualitative studies of the available evidence^6,9,13^, which includes ancient itineraries and physical remains including preserved roads, milestones and bridges. There are several regional studies that have analyzed these engineering tasks applied to mountainous or irregular regions^14,15^. Previous quantitative studies tend to focus on a single structuring feature such as topography or network structure, or were limited to particular provinces or regions, or used an incomplete empire-wide dataset of low spatial resolution^16–28^. Previous studies have emphasised a number of long-held assumptions^25,27,29–32^ about Roman roads, including the high density of roads during Roman occupation, a tendency towards straight and paved roads, the centrality of Rome in the road network, and a high degree of correlation between present-day and Roman roads. Yet a quantitative and empirically-informed evaluation of these assumptions at an Empire-wide scale has never been performed. We perform a detailed descriptive approach to the roads’ topographic and network structure, which is complementary to previous work in economic history and historical economics that focused on identifying causal inference. This descriptive approach is crucial since we do not currently know how human interaction potential was shaped by road infrastructure during the Roman period, which in previous qualitative work is often inferred by abstract knowledge of ancient connections between places rather than by concrete spatially explicit paths crossing real-world topography.

Here we present a spatially detailed quantitative exploratory study of the structure of roads of the entire Roman Empire, made possible thanks to our work in creating the most comprehensive and detailed digital model of 299,171.31 km of roads of the Roman Empire^33^. We focus in turn on evaluating long-standing questions: (1) Were Roman roads predominantly straight?^9,29^ (2) What was their continuity up to the present day?^17,18,30^ (3) How central were major cities and Rome itself in the network?

## Results

### The straightness and topographic structure of roads

**Fig. 1:**
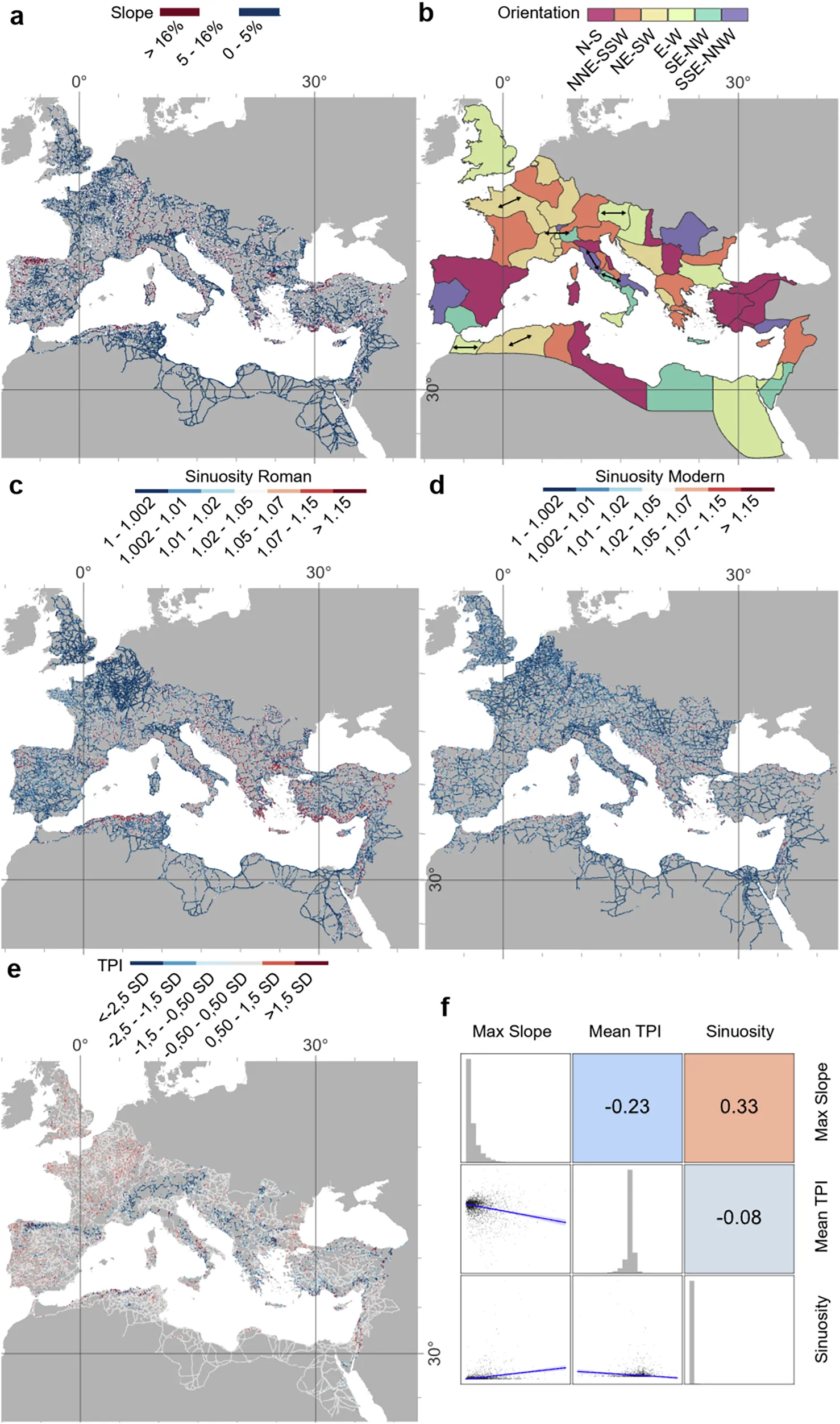
Topographic variables. **a** Maximum slope. **b** Dominant orientation of roads by province. Arrows indicate provinces where the dominant orientation of roads follows the orientation of mountains. **c** Roman Road sinuosity. **d** Sinuosity of modern roads. **e** Topographic position index (TPI) of the Roman roads, negative values indicate valleys, high positive values indicate ridges. **f** Matrix summarising the relationship between the topographic variables, lower left: scatterplots with linear trend, diagonal: histograms, upper right: Pearson’s ρ values. All variables were measured for 1000 m long road segments.

We analysed four properties that describe basic characteristics of Roman roads in their local topography: average and maximum slope, mean TPI (Topographic Position Index), orientation, and sinuosity (Fig. 1 and Supplementary Datasets 1–2). TPI is a terrain attribute defined as the difference between the elevation of a cell and the average elevation of cells in a given neighbourhood^34^. Sinuosity can be used as a measure of ‘straightness’ of the road, calculated as a ratio of actual road length to straight line distance between start and end point of 1 km long road segments. These metrics together provide us with valuable insight into the process of planning and construction of Roman roads, concerning constraints of ancient technology and transportation. Results were derived for 1 km stretches of roads to enable comparability. These results do not change much when taking road certainty categories or road types into account (see ‘Topographic structure by certainty’ and Supplementary Fig. 1 for details).

How straight were Roman roads? Sinuosity is very low in general (mean 1.048; median 1.0045) with most low sinuosity roads located in lowlands (mainly in Western Europe, the Pannonian basin, North Africa, the Po valley, Puglia, central Anatolia and Syria), where their course is not heavily dictated by the topography and straight-line connections can be more easily maintained (Fig. 1c). High sinuosity roads are found in mountainous areas and they predominate in Southeastern Europe. However, these results also reveal the variability in data collection efforts and resolution for different parts of the empire, as represented in our representativity map^33^.

The straightness of Roman roads is in part a function of topography. In flat terrain connections between two points will tend to be straight, radially extending from the settlements, as can be best seen in Northwest Europe. We observe that sinuosity is slightly positively correlated with maximum slope (Fig. 1f), i.e. growing maximum slope correlates with higher sinuosity of the roads. On the other hand, sinuosity is slightly negatively correlated with TPI, but their relationship is more complex. To a certain point an increasing TPI is correlated with growing sinuosity, but very high TPI values are rather correlated with lower sinuosity. The explanation might be that high TPI values are more associated with ridges which can support more straight roads than lower TPI values which rather indicate more complicated topography (upper/middle slopes etc.). In addition to topography, the Romans brought technological improvements which allowed planning and building (straight roads) more effectively, such as surveying instruments^29,31^ and construction methods^3^. Nevertheless, major modern roads (Fig. 1d) tend to be straighter than ancient roads, with lower mean and median sinuosity values (1.024 and 1.0026 respectively). The simple explanation is that modern road building avoids sharp turns and aims to build as straight roads as possible by strongly modifying the terrain to facilitate faster travel by car, which is made possible by modern surveying and construction methods.

The slope value (Fig. 1a) tells us about potential limits of each road segment regarding a suitable mode of transport, since animal-drawn carts and wagons are limited to gentle slopes. The upper slope limit for using vehicular traffic is thought to be anywhere between 5 and 16%, or between 2.86° and 9.09°^14^. The vast majority of roads (270,955.85 km of roads or 90.57%) are within the limits of possible vehicular traffic below 16% rise (maximum slope less than 9.09°). High slope values are located in the mountainous areas. Interestingly, the Alpine roads show lower slope values (the mean of average slope values is 2.68°; mean of maximum values is 6.79°) compared to roads in other mountain regions e.g., Taurus (3.33° and 8.11°), Pyrenees (3.14° and 7.85°), Cantabrian Mountains (4.98° and 11.26°), Atlas (2.92° and 7.06°), and Greece (2.86° and 7.11°). Most values fall between 0° and 10° (0-17.63% slope). The global median slope of the whole dataset is 3.88% (2.22°). This pattern is likely explained by two complementary factors. First, although the Alps constitute the highest mountain system in Europe, they also include a set of major longitudinal valleys and relatively favourable passes that can be exploited to maintain gentler gradients along long-distance corridors, compared to other mountain ranges where routes may be forced into steeper, shorter ascents and descents. Second, and probably more importantly, the comparatively low slopes of Alpine roads reflect a strong Roman engineering and strategic commitment to ensuring reliable overland connectivity from Italy to the rest of Europe. In this context, Roman surveyors and builders did not merely accept the constraints of the terrain, but actively intervened to reshape it where necessary through large-scale cuttings, terracing, retaining structures, and, in exceptional cases, tunnels, as exemplified by the rock-cut sections associated with the road system of the Aosta Valley^35^.

Most of the road segments fall into low TPI values (mean −17.8, median −4.98), which suggests that the preferred locations for the roads were flat areas or valleys, whereas fewer roads are located on slopes or prominent ridges (Fig. 1e). This pattern might be partly caused by higher representativity of the dataset for the low-lying regions, especially north-west Europe (see representativity map in de Soto et al.^33^). What stands out is the prevalence of high negative values indicating roads in valleys, especially in mountainous areas, south-eastern Europe and Asia Minor. On the other hand in most of north-western Europe, the Iberian peninsula, and North Africa the majority of roads are located in moderate topography, in level areas, on gentle slopes or small elevations.

In mountainous areas the road orientation is strongly determined by topography, it follows the orientation of mountain ranges and valleys, and cuts through mountain passes. This is most striking in a few provinces where the overall road orientation follows the overall orientation of mountain ranges (Fig. 1b): Alpes Graiae, Etruria (Regio VII), Latium et Campania (Regio I), Lugdunensis, Mauretania Caesariensis, Mauretania Tingitana, Noricum. However, in other provinces roads are overall perpendicular to mountain ranges: Africa Proconsularis, Asia, Bithynia et Pontus, Galatia, Hispania Citerior, Iudaea, Moesia Superior, Umbria (Regio VI). In non-mountainous regions other factors determined the orientation, including the settlement structure and river valleys.

### Roman and major modern road density correlate

**Fig. 2:**
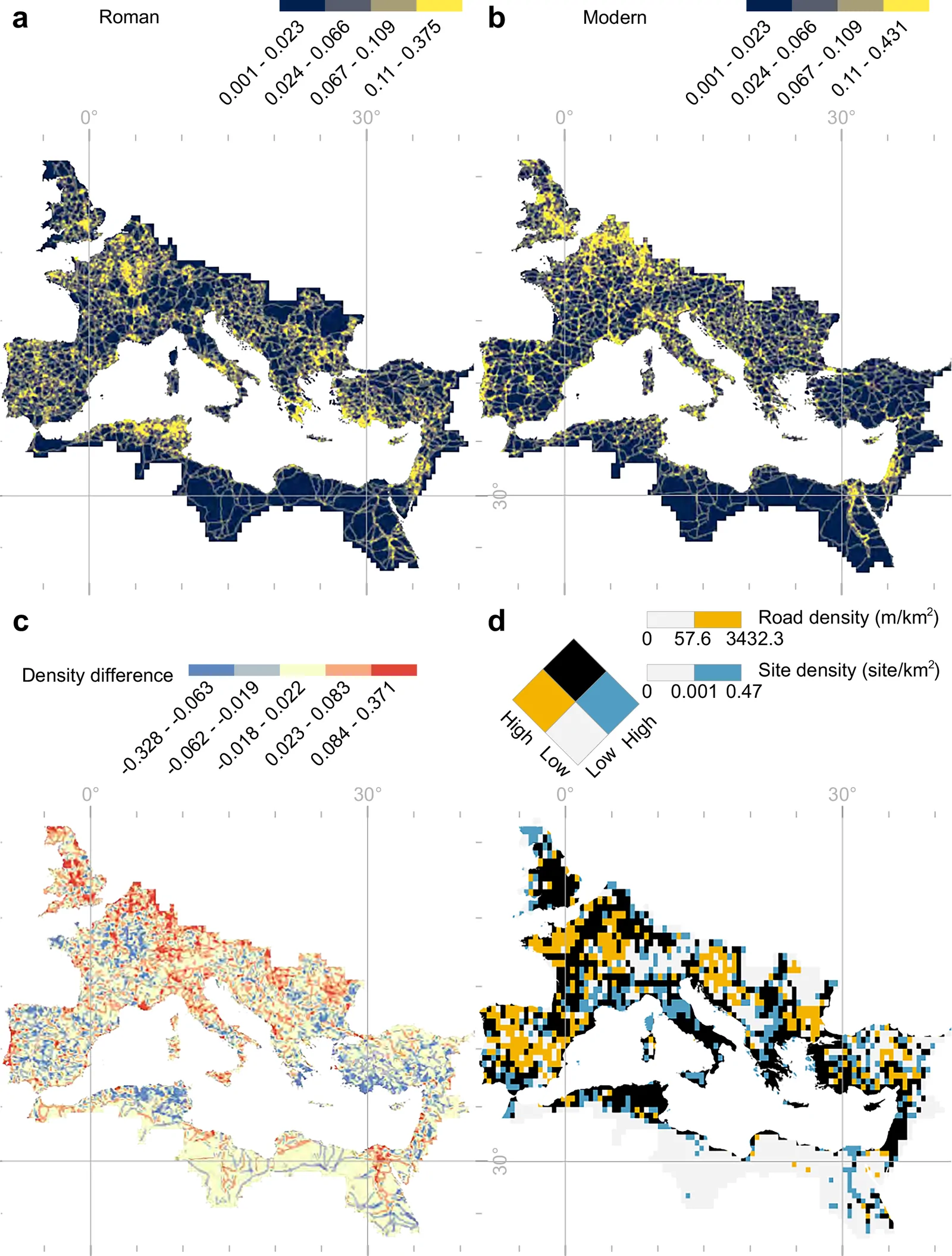
Density of roads. **a** kernel density of Roman roads. **b** kernel density of modern roads. **c** Difference of kernel density between Roman and modern roads, negative values indicate a decrease in road density from the Roman period, positive values indicate an increase in road density since the Roman period. **d** the relationship between the density of Roman roads and the density of Roman sites.

What is the relationship between Roman roads and major modern roads? The difference in kernel density between modern (Fig. 2b) and Roman roads highlights areas exhibiting significant changes in the road density between the Roman and modern period (Fig. 2c). We observe a conspicuous increase in road density in the core urban and industrial areas of Western Europe (northern Italy, Rhine-Meuse basin, Britain) and highly concentrated change around large urban centres (e.g., Paris, Marseille, Madrid). Areas with a lower density of modern roads are located mainly in Greece, Asia Minor and Northern Africa. The patterns observed in other regions are results of more varied processes. The differences in road density in the Iberian Peninsula can be related to Modern era centralisation tendencies focused on Madrid^22^. The case of Egypt shows increased urbanisation and rising importance of vehicular transport in the Nile Valley compared to the past when the country was reliant mainly on riverine transport^36^. However, the lower representativity of the dataset for certain regions (central Europe, western Balkans, Tuscany, Marche, central Anatolia, see de Soto et al.) can skew the results so that the difference between Roman and modern road density will be larger.

**Fig. 3:**
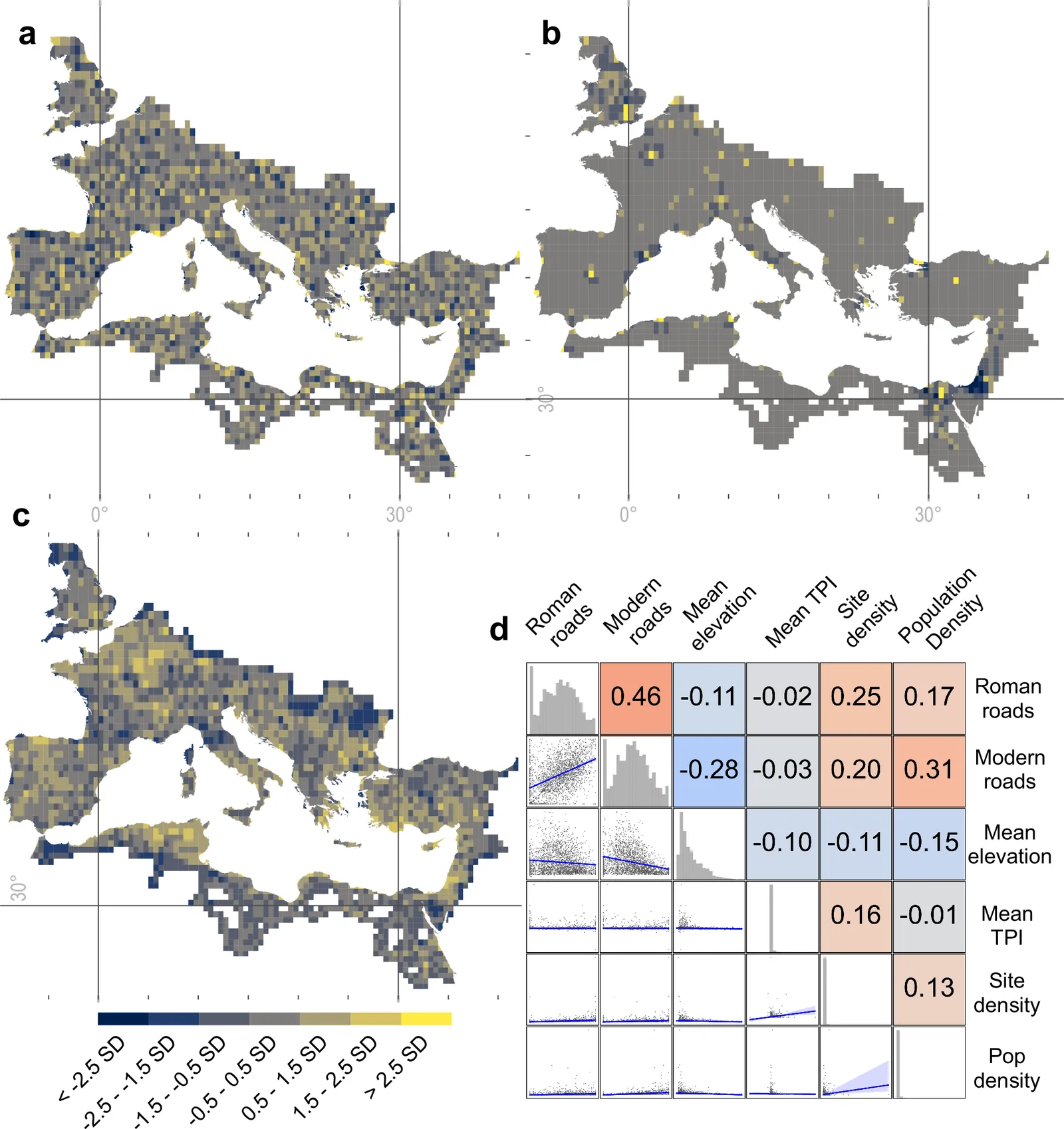
Geographically weighted regression analysis, showing standardised residuals of the model. **a** scenario 1: relationship between Roman and modern roads. **b** scenario 2: relationship between Roman roads and modern population. **c** scenario 3: relationship between Roman roads and topographic and anthropic variables (mean elevation, mean Topographic Position Index, site density). **d** linear regression of all dependent and explanatory variables (continuous ordinary least squares model), lower left: scatterplots showing linear trend and 95% confidence intervals of the fitted regression line, diagonal: histograms, upper right: Pearson’s ρ values. Asterisk shows a statistically significant relationship (*p* < 0.05): Modern roads *p* < 0.0001, Site density *p* = 0.029.

We subsequently explored the relationship between the location of Roman and major modern roads, and several key topographic and anthropic variables (elevation, TPI, density of Roman sites, and modern population, see ‘Linear regression’), using a 0.5° cell grid as the analytical unit (using the provinces as analytical units provides comparable results, see Supplementary Fig. 3). The results (Fig. 3d, Supplementary Table 3) indicate a positive correlation between locations of Roman and modern roads (Pearson’s ρ 0.46). Modern roads are positively correlated with Roman site density (0.2 for 0.5° cells and 0.37 for provinces), but less strongly than Roman roads are (0.25 for 0.5° cells and 0.45 for provinces). However, correlation with the modern population is lower (Pearson’s ρ 0.17). The topographic variables show very low negative correlation to Roman roads (Pearson’s ρ −0.11 for elevation and −0.02 for TPI) which suggests that topography has a slightly negative impact on the location of Roman roads: slightly fewer roads with growing slope or TPI. Modern roads are slightly more negatively correlated with elevation and TPI than Roman roads are (Pearson’s ρ −0.28 and −0.03).

We further explored the spatial relationship of these variables using Geographically Weighted Regression (see ‘Spatial regression’) in three scenarios: (1) whether the relationship between locations of modern roads and locations of Roman roads is consistent throughout the region, (2) whether the relationship between the density of the modern population and locations of Roman roads is consistent throughout the region, and (3) what is the explanatory power of selected environmental and anthropic variables (density of Roman settlements) on the location of the Roman roads. The results (Fig. 3a–c; Supplementary Table 4) show considerable spatial heterogeneity in scenario 1 with contrasting over- and under-predictions which are not organised into any large contiguous regions. The model explains a large degree of the variance (adjusted R^2^ = 0.61), but the results suggest that the relationship between Roman and modern roads is mediated by local (micro- to meso-level) circumstances. The model in scenario 2 does not explain much variance (adjusted R² = 0.25). The relationship between modern population and Roman roads is weak, but spatially consistent. The positive and negative residuals are concentrated in the main population and urban centres across the whole research area. In this case, our evidence is likely biased towards modern population centres where archaeological investigations are concentrated due to modern development, and recovery rates of archaeological remains are larger there than in the countryside. Model 3, the combination of Roman site density, elevation and TPI values, explains only a small part of the variance (adjusted R^2^ = 0.35). We again observe a highly locally varied and spatially heterogenous pattern, including systemic overprediction in North Africa, the southern Levant, southern and central Italy, southern Greece, Bulgaria and the Danube frontier, central and northern France, Britain, and central Spain. This suggests that the included variables capture only a limited and spatially uneven share of the processes shaping the location of Roman roads which are mediated at a micro- to meso-level.

The robustness of these results was explored by changing cell size, the neighbourhood size, and comparing with data confidence (see ‘Robustness of spatial regression’). Comparable results are obtained when using smaller 0.25° cells (Supplementary Fig. 5; Supplementary Table 6), although the resulting models for scenarios 1–2 perform worse. We equally observed worse performance when using a 1000 km neighbourhood for models of scenarios 1–2 (with different substantive results for scenario 1) and using an optimal number of neighbours for scenario 3 (Supplementary Fig. 5; Supplementary Table 5). Comparison with data reliability and representativity reveals results to be more robust in north-western Europe, Spain, Italy, Greece, the eastern Balkans, most of Asia Minor, the Near East, the Eastern Desert of Egypt, coastal Cyrenaica and Tripolitania, and North Africa. Finally, comparable results are obtained for scenario 1 when excluding cells with low representativity or reliability, although the model explains slightly less variance (adjusted R^2^ 0.53 as compared to 0.61; Supplementary Fig.6 and Supplementary Table 7).

There are significant differences in the density of Roman roads across the Empire. The dataset contains 299,171.31 km of roads in a total study area of 5,271,920.243 km^2^, giving a global road density in the Roman Empire of 56.748 m/km^2^ (Fig. 2a). High road density is observed in most of the densely urbanised regions of the Roman Empire. These include areas with a long history of urbanism and intensive trade activity (Italy, Greece, Asia Minor, Tunisia). Other high density areas correspond to the militarised border regions with long-term concentrations of army units creating conditions for urbanisation and development of (road) infrastructure (lower Rhine, middle and lower Danube)^37^. Low density areas include the marginal mountain and desert regions where habitability and roads are restricted, such as the Alps, Pyrenees, the southern Balkans, the Sahara and the Syrian desert. Some highly urbanised areas have lower road densities (such as Baetica, Hispania Tarraconensis or the Northern Levant), since their roads are concentrated to valleys or coastal plains strongly bounded by mountains. These densities also highlight the long-term effects of differential research intensities and data availability, where the road structure of intensely researched parts of the Roman Empire such as Latium, the Southern Levant, or Northern France, are particularly well known (compare confidence map in de Soto et al.)^33^. Density of ancient sites is positively correlated with Roman roads at a provincial scale (Pearson’s ρ 0.45, Supplementary Fig. 3b), but the correlation is weaker (Pearson’s ρ 0.25, Fig. 3d) when using 0.5° cells as analytical units (see ‘Linear regression’), which likely reflects lower representativity of the road dataset in certain regions covered (see representativity map in de Soto et al.)^33^. Some areas are revealed to have a high road density and low settlement density, suggesting an underrepresentation of ancient places in the dataset used (Fig. 2d, see ‘Ancient sites’) such as in the central Iberian peninsula, northern and central France, western Balkans, and Bulgaria. Other areas demonstrate the opposite case of low road density and high site density, identifying underrepresentation in the roads dataset (northern Britain, Tuscany and Marche in Italy, Corsica, south-west Germany; representativity and reliability in the road data is discussed in de Soto et al. 2025 and in ‘Robustness of spatial regression’)^33^.

### The structural location of major cities on the Empire-wide network

How central were major cities and Rome itself on the Roman road network? This question directly relates to the broader issue of transport-network design and to the role of cities in it. We answer this question by first exploring the location of cities on the Empire-wide network, we subsequently explore the most central roads per province, and finally combine this information with the centrality of cities per province to reveal the diverse ways in which roads structured the opportunities of city populations including Rome’s.

The Empire’s road network data representation comprises 10,516 nodes (representing intersections and dead ends) and 14,901 edges (representing roads), forming 13 connected components. The network’s Gamma index, Alpha index and average degree are strongly correlated (Supplementary Dataset 4, see ‘Network analysis’), as expected, since in the case of planar graphs they are all indices of density. To establish the significance of these values, we compare the results of some of the above measures with those of major modern roads (Supplementary Dataset 5). The Gamma Index is consistently higher for Roman roads than for major modern roads, reflecting great network density. The Alpha Index and global clustering coefficients are also generally higher. Although this result compares all Roman roads with only the major modern roads, it still indicates a remarkable level of terrestrial interconnectedness in Roman times.

**Fig. 4:**
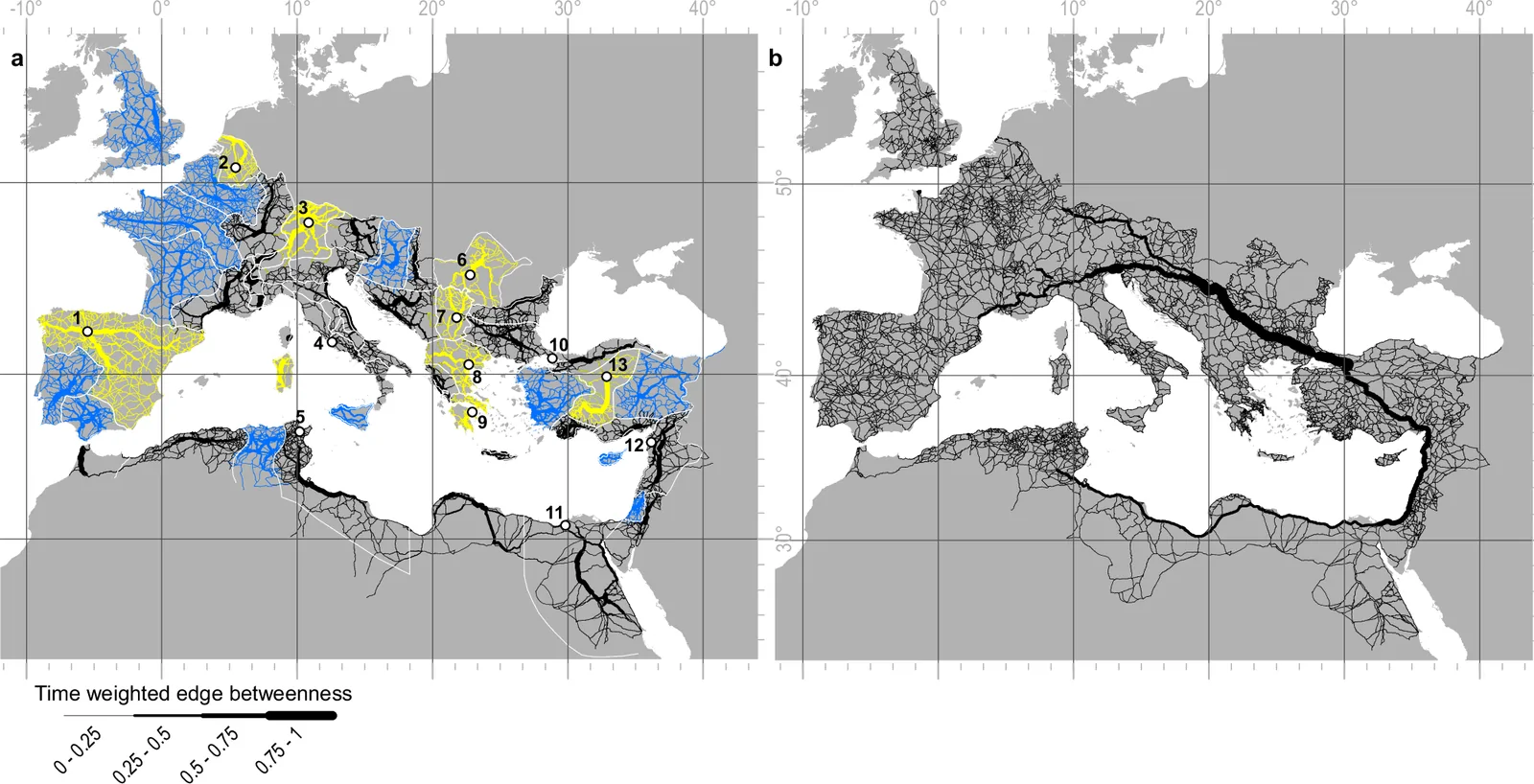
Network representation of the roads of the Roman Empire, line width represents time-weighted edge betweenness. **a** Results per Roman province (marked by white borders) reveal differences in the structure of key intermediary roads to provincial terrestrial movement: linear (black), distributed (blue), or centralised (yellow). **b** Results for the entire empire, revealing the importance of the Mediterranean coastal roads and the intercontinental crossing at Byzantium. Selected major cities marked for reference (including focal cities in centralised provinces): 1 Legio-León, 2 Atuatuca-Tongeren, 3 Augusta Vindelicorum-Augsburg, 4 Rome, 5 Carthage, 6 Sarmizegetusa, 7 Naissus-Niš, 8 Thessalonica, 9 Corinth, 10 Byzantium, 11 Alexandria, 12 Antioch, 13 Ancyra.

We computed a travel time weighted edge betweenness centrality^38^. This metric quantifies the segment’s importance as an intermediary for terrestrial connectivity, by measuring the proportion of shortest paths passing through each road segment based on travel time using Tobler’s hiking function (see ‘Travel time weighted edge betweenness’). Figure 4b demonstrates the importance of the Mediterranean coastal roads for mediating terrestrial movement throughout the entire empire, in particular in North Africa, the Levant, southern France, and northwestern Spain. It further highlights the structuring effects of natural features like rivers and valleys (Po valley, Rhine, Danube, Drava, Maritsa, Tundzha) and cultural features like the Roman frontier (Rhine, and Danube to some extent) and important urban settlements: the high betweenness path passes Lepcis Magna, Alexandria, Caesarea Maritima, Antioch (Antakya), Nicomedia (İzmit), Byzantium (Istanbul), Philippopolis (Plovdiv), Serdica (Sofia), Aquileia, and Narbo (Narbonne). Indeed, 24 out of 45 provincial capitals are located in the top two deciles for empire-wide edge weighted betweenness centrality (see Supplementary Dataset 7 for empire-wide centrality scores of provincial capitals). Byzantium is highly centrally located in the continental road network as a whole, since it connects the European and Asian components of the Roman road network (via a ferry crossing the Bosporus, another ferry connecting the European and Asian components is included at the Dardanelles^39^, see ‘Network analysis’). Interesting deviations include the high betweenness values for roads going south of the Danube, following the Drava instead and crossing the eastern Alps near Graz until it reaches the Rhine near Mannheim. The general decrease of values from the centre to the periphery is to be expected for a betweenness metric, but it equally highlights peripheral areas in the continental Roman Empire’s road system, including Tunisia, the Sahara, most of Egypt, the Syrian desert, most of northeastern Turkey, Greece, Dacia, Italy, and the entire Atlantic facade. The connectivity of many of these regions should instead be understood as mediating contacts within the province, across the frontier (Dacia, Syria, Sahara), via rivers (the Nile valley in Egypt), or maritime (Greece, Italy, the Atlantic facade, Tunisia, the Red Sea coast, north Turkey). Such terrestrial mobility results are hard to divorcee from the reliance on river- and seaborne transport for crossing large distances. Roads were more essential for connecting inland areas and enabling effective short- to mid-distance travel such as travel within provinces.

### Main roads central in provincial road networks

What were the most central roads in each province? Much has been written on the typological diversity of Roman roads and on how they should be classified. When referring to these routes using ancient terminology, one may invoke *viae publicae*, *viae vicinales*, and *viae privatae*^9^. For several decades, however, scholarship has tended to adopt a simpler dichotomy between primary and secondary roads. The former seems to comprise the major arterial corridors that structured overland connectivity across the Empire and are commonly recorded in the *Itineraria*, or marked by milestones^6^, yet quantitative evidence-driven confirmation that they could indeed have been more important (in highly specified ways) for structuring mobility are absent.

**Fig. 5:**
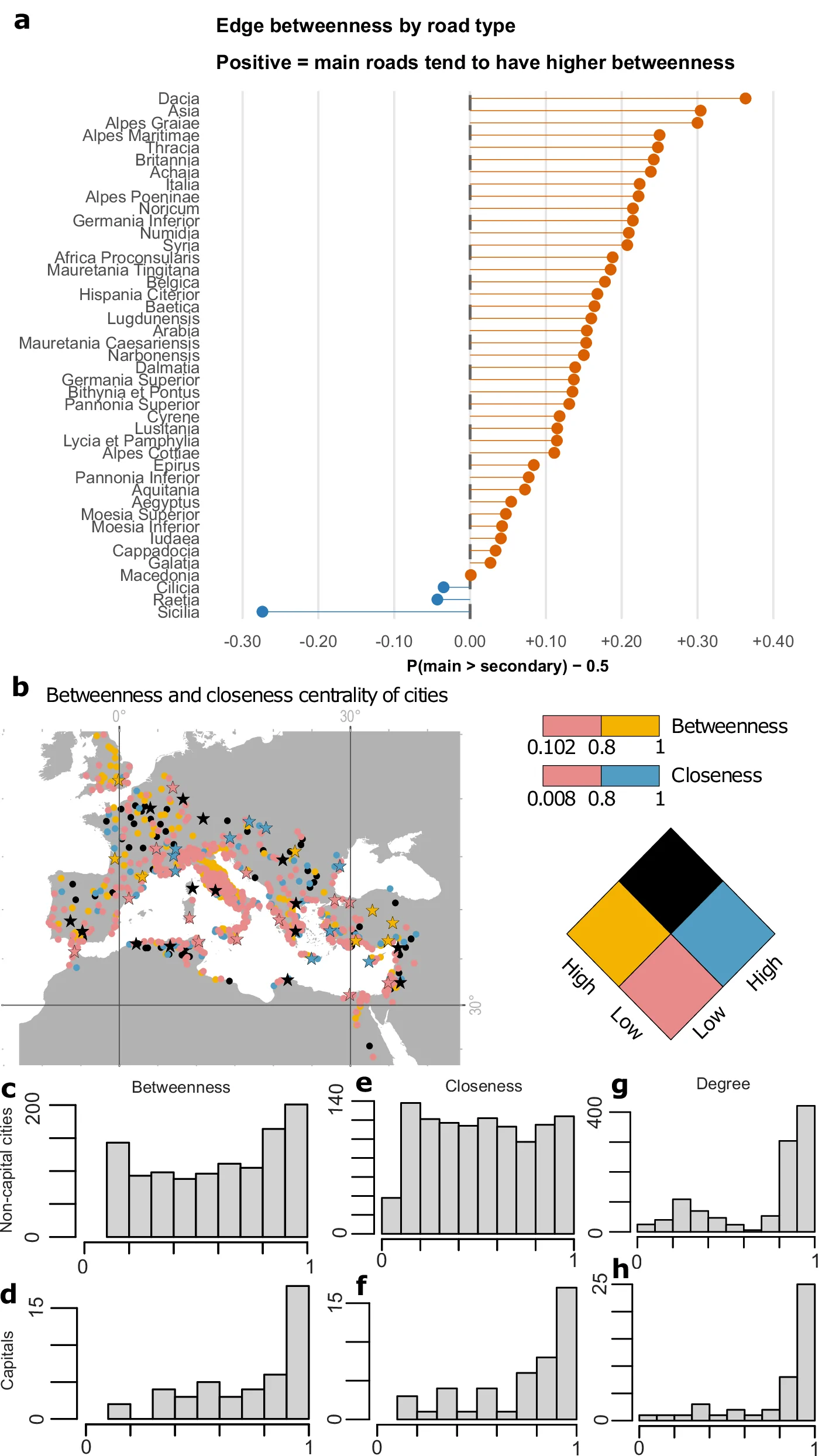
Centrality of roads and cities. **a** The lollipop chart shows, for each province, the probability that a randomly selected main road has higher betweenness than a randomly selected secondary road, providing a distribution-based measure of the relative strategic importance of the two road types. **b** betweenness and closeness centrality of Roman cities, provincial capitals are represented by a star and tend to have high betweenness or closeness or both, and those who do not are major maritime or riverine ports (in white, e.g. Alexandria, Lugdunum). **c**–**h** histograms of centrality deciles for closest nodes to cities. Capitals are skewed to the top 2 deciles of betweenness and closeness.

Across nearly all provinces with both main and secondary roads (40/43), main roads are more likely than secondary roads to have higher time-weighted edge betweenness (median probability = 0.65), indicating that they occupy more strategically important positions within the network (Fig. 5a). This shows the potential of betweenness to identify main roads in the Roman road network, and supports the interpretation that those sections classified as main roads were key intermediaries in Roman terrestrial transport. The degree and edge betweenness results are robust to random removal of uncertain road segments with a 20% probability (see ‘Robustness of network analysis results’).

The betweenness results further highlight roads that were important intermediaries for terrestrial mobility in each province, revealing a linear, centralised or distributed structuring of the roads in Roman provinces (Fig. 4a). Key intermediary roads are often structured linearly by a river (the Nile: Aegyptus; the Rhône: Gallia Narbonensis) which also marks the Roman frontier (the Danube: Pannonia Inferior, Moesia Inferior; the Rhine: Germania Superior), or along the coastline and the coastal mountain ranges around the Mediterranean (Italia, Epirus, Dalmatia, Lycia et Pamphylia, Cilicia, Bithynia et Pontus, eastern part of Africa Proconsularis, Mauretania Caesarensis, Cyrenaica). Interesting deviations include the frontier provinces Moesia Superior and Pannonia Superior, where the central roads are not along the Danube and through the provincial capitals, but rather centre on inland supply routes. Mountain ranges and passes structure key intermediary roads in mountainous parts of inland provinces (Noricum, Thracia, Alpes). In some provinces the high betweenness roads are revealed as being centred on focal settlements, from which the rest of the province can be effectively reached (Naissus in Moesia Superior, Sarmizegetusa in Dacia, Augusta Vindelicum in Raetia, Ancyra and Iconium in Galatia, Atuatuca in Germania Inferior, Corinth in Achaia, Thessalonica in Macedonia). Other provinces instead demonstrate a wider distributed coverage with multiple high betweenness roads (Pannonia Superior, Cappadocia, Asia, Britannia, Iudaea, western part of Africa Proconsularis, Numidia, Gallia Aquitania, Gallia Lugdunensis, Gallia Belgica, Hispania Tarraconensis, Lusitania, Hispania Baetica).

### Capitals central in provincial road networks

Were major Roman cities and provincial capitals characterised by central locations on the provincial road system? Nodes with high centrality values play an important role in the road network, serving as major local crossroads (degree centrality), strategic points along shortest paths (time-weighted betweenness), or locations that are quickly reachable from across the system (time-weighted closeness). The time-weighted betweenness and closeness histograms (Fig. 5c–f) reveal that capitals tend to be particularly centrally located as reachable places or intermediaries in a province when compared to other cities. Provincial capitals tend to be in central locations (35 capitals have at least one centrality score in the top decile; 14 out 45 capitals have all three scores in either top or second deciles) and particularly on major local crossroads (degree centrality: 25 capitals in top decile, plus 8 in second decile, Fig. 5h; although all cities tend to have high degree scores since roads connect cities, Fig. 5g). Capitals with no centrality score in the first decile are important riverine (Lugdunum, Colonia Agrippina) or coastal ports (Carthage, Alexandria, Tingis, Caesarea Maritima, Perinthus, Caralis). These results quantitatively confirm Roman provincial capitals are beneficially located for either maritime or provincial terrestrial mobility, or both (Rome, Antioch, Corinth, Corduba, Alalia, Nicopolis, Iol Caesarea, Viminacium; Fig. 5b). The estimated population of major Roman cities is not correlated with centrality on the road network, with the exception of a weak but significant Spearman correlation between population and degree centrality in Italia (ρ = 0.22, *p* < 0.001), and Britannia (ρ = 0.58, *p* < 0.01); a significant correlation between population and closeness centrality in Achaia (ρ = 0.54, *p* < 0.001); and in Germania Superior both closeness (ρ = 0.69, *p* < 0.01) and betweenness (ρ = 0.73, *p* < 0.01).

## Discussion

Our exploratory analysis of the topographic and network structure of the roads of the Roman Empire, presents spatially detailed answers at an Empire-wide scale to long-standing questions: were Roman roads straight, what was their relationship to the location of major modern roads, and how central were cities to structuring flows in the road network? Our findings are complementary to those of previous research in the economics literature on long-term persistence which, instead of the exploratory and descriptive spatial methods used here, uses econometric methods to identify causal relationships^19,21,24–27^. These studies have been performed on smaller parts of the Empire using lower resolution road data, and we argue our work can facilitate more comprehensive analyses in economic geography, urban studies, and transport geography, by integrating the spatially-detailed empire-wide dataset in their models. Our work further goes beyond previous comparable qualitative and exploratory research through a much higher degree of specification: we specify under which conditions Roman roads are straight, how straightness and correlation with major modern roads varies precisely throughout the Empire, which cities are central, how central they are, and what we mean by centrality.

How straight were Roman roads? Our results can inform ongoing debates to reassess what has traditionally been interpreted as the straightest road system of antiquity, by highlighting the variability of how road construction was shaped by the physical environment and where shorter but more sinuous routes were preferred. We conclude that Rome created a road system where straight alignments were consistently favoured when the terrain permitted, but at the scale of the whole empire the common assumption that Roman roads were uniformly rectilinear is not accurate. This pattern suggests planning aimed at improving directness and efficiency within technological and topographical constraints, rather than pursuing straightness at any cost.

The general ‘straightness’ of Roman roads is explained by them crossing such areas where topography enabled the construction of rather straight roads. Main roads, both split into 1000 m segments and unsplit, exhibit slightly lower sinuosity values than secondary roads, closer to modern roads (Supplementary Table 1, median sinuosity split: main roads 1.0035, secondary roads 1.0051, modern roads: 1.0026). Therefore, a case for straight Roman roads might be made with regard to the main roads, which likely represent public and military roads (*viae publicae*, *viae militares*), or axes of land divisions (*limites*) laid out and engineered or adapted by specialists (*mensores*, *gromatici*).

Our result that Roman roads are less straight than major modern roads is to be expected. While the Romans had highly accomplished engineers, capable of constructing bridges and tunnels, and at times cutting through or levelling terrain to maintain viable alignments^40,41^, present day technologies allow for far more extensive and systematic landscape modification. Modern surveying and large-scale earthmoving enable straighter, high-speed road corridors that are less constrained by local topography, whereas ancient roads more often negotiated the surrounding topography.

Mountain ranges, river valleys and settlement distribution are important structuring features for the location of Roman roads. The vast majority of roads (270,955.85 km of roads or 90.57%) are within the limits of possible vehicular traffic below 16% rise, and are built in areas with low topographic variability. Mountains provide the most formidable barriers to wheeled traffic in the Roman Empire, but the relatively low slope values of Alpine roads as compared to other mountain ranges reflects its major valleys and passes as well as significant resources in adapting the terrain to facilitate terrestrial transport out of and into the Italian peninsula, as demonstrated e.g., by the works on the Aosta Valley road^35^. The mountain roads are those where the difference between Roman and modern road sinuosity is most pronounced, as climbing strategies for walking (including pack animals) on steep slopes involve considerable use of switchback paths (zigzagging)^42^.

As a result of these calculations, our conclusion that Rome did seek to build straight roads wherever terrain conditions allowed confirms and expands on previous scholarship on Roman road planning^31^. At the scale of the whole Roman Empire, however, the common assumption that Roman roads were uniformly rectilinear is not entirely accurate, even if straight alignments were consistently favoured when feasible. As expected, slope and local topography appear to be the main factors shaping the structure of Roman roads. Thus, although Roman engineers could modify the landscape to a considerable degree, they could not avoid a minimum level of sinuosity. In this sense, geographic conditions directly structured the network. This is particularly clear in provinces of southern Europe and the Mediterranean basin, where more pronounced relief is associated with fewer straight roads, in opposition to north-western Europe, the Po valley, and the Pannonian basin with fewer obstacles for engineering straight roads.

What was the continuity of Roman roads up to the present day? Because we are comparing two networks separated by almost 2000 years, a wide range of historical, political, and economic processes have necessarily shaped the contemporary system. Traces of the Roman network are therefore unsurprising: many present-day cities either originated in, or already existed during, the Roman period, and connections between them often have deep historical roots. Over the intervening centuries, however, shifting demographic patterns, state formation, and changing economic priorities have also generated new motivations for building infrastructure where none previously existed, fostered the emergence of new communication hubs, and, conversely, reduced the strategic or economic relevance of other regions and corridors. Globally, Roman roads are moderately positively correlated (Pearson’s ρ 0.46) with major modern roads, consistent with a previous study on a less detailed dataset on an empire-wide scale^25^ and for the case of Italy^27^. Our more spatially detailed results allow for the identification of a conspicuous increase in road density in the core urban and industrial areas of Western Europe (northern Italy, Rhine-Meuse basin, Britain) and highly concentrated change around large urban centres (e.g., Paris, Marseille, Madrid). This increase is most pronounced in small ancient population centres that grew into major metropolitan areas (e.g. Birmingham, Brussels, Paris, Madrid). Areas with a lower density of modern roads are located mainly in Greece, Asia Minor and North Africa (Tunisia and Algeria), which were exceptionally densely urbanised areas in the Roman Empire. These differences reflect long-term historical shifts in terrestrial infrastructure driven by industrialisation, and urbanisation within nation states.

The high spatial resolution of our results and the wide coverage of our Roman roads dataset reveal enormous local variability in the effect of Roman roads on modern roads, suggesting the importance of local-scale mechanisms, in addition to the regional or continental scale ones focused on in previous studies. Our geographically weighted regression model (GWR) in turn reveals that the relationship between Roman and modern roads is overall strong (adjusted R² = 0.61), but it is shaped by highly local conditions. This conclusion broadly corresponds to findings by Dalgaard et al., who have identified such persistence in transport infrastructure through correlations between Roman and present-day road density, as well as exploring persistence in economic prosperity through comparisons with present-day nightlights and population density^25^. In contrast, our spatial regressions did not reveal a strong relationship between Roman roads and modern population densities, suggesting a weaker pattern of long-term persistence along this axis of population density only.

We further notice that there is no geographically continuous region where Roman roads alone have strong local association with modern road infrastructure. Model residuals show highly heterogeneous patterns throughout the research area. Which might suggest different trajectories of historical development across the former Roman Empire, down to the very local level. These results are more complementary to previous research in what concerns the correlation between Roman and modern roads, where high local variability was revealed: the persistence of the Roman road system into the later periods as one of the explanations for long-term persistence of transport networks, economic activities, integration and sustained economic development was demonstrated by Flückiger et al. for Western Europe^26^, by De Benedictis et al. for Italy^27^, and by Wahl^21^ for the formerly Roman part of Germany. Bosker and Buring further demonstrated the importance of Roman roads in shaping urban development throughout the following two millennia^43^. The dataset used in this research can thus be used for enhancing the empirical basis of studies of long-term persistence in economic activities^19,21,24–27,43–46^, which have identified correlations between pre-industrial and present-day prosperous places.

Local regression slope coefficients and t-scores reveal regions where the location of Roman roads has a positive local effect on the location of modern roads and are statistically significant (Supplementary Fig. 8). They are found mostly in North Africa and the Near East with few locations around the Mediterranean and in Britain. This result puts into question the previous finding by Dalgaard et al.^25^ where high correlation between Roman and modern roads was postulated for Western Europe and low correlation for North Africa and the Near East (explanatory power of the model not just slope magnitude), explained by changes in modes of transport (abandonment of vehicular traffic) in the Medieval period^25,47^.

Our findings imply that while we well capture the dominant spatially varying relationship, there are highly localised processes that influence the location of modern roads. The linear regression model hints at some of the possible local processes: (a) local topography (elevation, TPI), to which we may add (b) influence of natural movement corridors (in mountainous areas) suggested by road orientation results, (c) Roman settlement patterns and persistence of the urban form, and finally (d) by historical and institutional development and persistence, which were not considered in this research. Our results should be explored in the future with different econometric methods and assessing the impact of the role of major Roman roads (which make up a much larger proportion in the dataset used in previous work) in driving long-term economic persistence, the different modern population datasets used, as well as the spatially detailed nature of the road dataset.

We demonstrate that road sections classified as main roads were key intermediaries in provincial terrestrial transport, as revealed by consistently higher edge betweenness centrality scores. As such, we have presented a metric that quantitatively describes how main roads structured terrestrial mobility (made possible due to the ability to compare with 195,693.3 km of secondary roads in our dataset). Previous research identified the main roads based on qualitative characteristics (presence of milestones, itineraries, etc.) and in turn assumed their importance. Our results independently confirm these previous assumptions using quantitative metrics. This result might reflect conscious choice by the Romans in planning and developing the road system on a macro-level. These important intermediary roads further revealed differences in the structures of provincial road networks (distributed, linear, or centralised), which indicates differences in how provincial administration and inhabitants were able to use the roads, and how the flow of people, goods and ideas were structured in provinces. A linear or centralised system affords intermediary centres to exercise significant control in trade, information flows or provisioning of a standing army (e.g. Naissus in Moesia Superior), but it is equally more vulnerable to targeted attack, blockades or failure in a central settlement. Provinces with a more distributed road network (e.g. the *Galliae*) are less vulnerable in that sense. These structures demonstrate how Rome used its road network to organise and structure each of the territories it dominated. Through road construction, the framework for mobility was built, and the territories were organised, facilitating transport between the main cities of each province.

How central were major cities in the network? We quantitatively confirm that Roman provincial capitals are beneficially located for provincial terrestrial mobility, more so than non-capital cities, and specify the nature of this centrality using metrics for all Early Imperial capitals. Capitals tend to be particularly centrally located as reachable intermediaries in a province when compared to other cities.

Despite the proverb ‘All roads lead to Rome’, major cities like Antioch, Ancyra, Byzantium and Corinth are better located than Rome as intermediaries for Empire-wide terrestrial mobility. Rome is spatially central in the Italian Peninsula (which is itself an appendix to the highest betweenness path on an Empire-wide scale) but Rome is not on the road with the highest time-weighted betweenness centrality in Italy. The current paper concerns the structure of terrestrial transport infrastructure only, and our results therefore cannot be seen to reflect all mobility through the empire. The importance of riverine and maritime mobility should be highlighted for most transport over long distances, for which Rome is highly centrally located in the empire. Future work should combine this road dataset with navigable rivers and sea connections and alternative modes of transport (e.g. horse courier, ox cart, pack animal), to identify the structure of the multimodal Roman transport system, which will reveal a much higher centrality of regions central in the Mediterranean (such as Italy, Greece, and Tunisia) and especially of major Mediterranean ports^18^. Scheidel has demonstrated how sea-lanes bind together the ‘core’ Mediterranean areas of the Roman Empire^18^. We further expand on Scheidel’s analysis by identifying capital cities with exceptional locations for both waterborne and terrestrial mobility (Rome, Antioch, Corinth, Corduba, Thessalonica, Nicopolis ad Istrum, Iol Caesarea, Viminacium, Augusta Vindelicum, Mogontiacum).

High time-weighted edge betweenness of the main roads and high time-weighted betweenness and closeness centrality of many provincial capitals when viewed together might imply system-level decision making on provincial and even supra-provincial level in developing the road system for the needs of the imperial power. This raises contentious issues whether the Roman state was capable of such planning and execution, how this planning could have been organised, what was the role of maps in this process and whether Romans were capable of producing maps that could support such planning^8,29,48–53^. We argue that our results (and particularly the high time-weighted betweenness of main roads and capital cities at a provincial and empire-wide level) support the conclusion that such planning and development of the Roman transport network was undertaken on a provincial level (and that empire-wide high betweenness centrality of provincial capitals could reflect empire-wide planning). However, our analyses do not reveal the causes, mechanisms or sequence of planning. Future work should focus on collecting the detailed temporal data about road construction and refurbishment to enable evaluating the explanatory power of factors that structured planning, such as the physical environment, the pre-existing road system, military conquest and pacification, and changes of provincial capitals and administrative divisions.

Caveats to our data analysis should be noted. Chief among these is that the road data used is the best available but not complete due to variation in the reliability and intensity of scholarly work and excavations/surveys across the research area, the heterogeneity across the dataset affecting its representativity, and the varying empirical basis of individual road segments. We performed robustness checks of our main results in light of data uncertainty, and future work could additionally integrate uncertainty into estimations directly. We demonstrated that degree and edge betweenness results are robust to random removal of uncertain edge segments with 20% probability. We provide concrete guidance for targeting future fieldwork and future improvements to the dataset, through our categorisation of road certainty and our confidence map^33^, as well as our identification of areas with high site density but low road density (Fig. 2d). At the same time, it is also important to point out that the creation of this absolutely extraordinary network of roads was a gradual process. The Roman road system was a development of earlier routes and changed significantly throughout the imperial period. This long development unfolded in stages, driven by various military, political, economic, and other motivations, all influencing the decisions made. The layout of these roads, therefore, is the result of the evolution of pre-Roman roads, military needs, policies of domination, regional and local interactions, and, above all, economic interests. Our results are derived from an aggregation of the available information and necessarily assume contemporaneity and continuous use of settlements and roads. Like the dataset itself, they are therefore more representative of the 2nd–4th centuries CE structure of the empire, because some settlements and major road infrastructure were not yet in existence in the Republican and early Imperial period, and the system would be less uniformly in use and integrated in late antiquity. For all these reasons, it will be crucial in future research to evaluate the network from a diachronic perspective, assessing the existing network at each historical moment and the modifications made in relation to past and future periods. Comprehensive and comparable empire-wide data on this temporal change does not exist and future work should focus on systematically collecting it to enable the evaluation of the robustness of our findings in light of the dynamic nature of the Roman road system: temporal changes in empire-wide and provincial-level road network structure, development of centrality of cities, and the structural impact of major infrastructure projects including on the economic, administrative and military needs of the time. A further challenge is that the structure of terrestrial transport is most valuable for understanding regional mobility, especially in landlocked places, rather than long-distance movement for which interlocking means of transport should be considered. Future work needs to integrate terrestrial routes with maritime and riverine routes and infrastructure, to create a model of ancient transport at a higher spatial accuracy than is currently available^54^. This would be an invaluable resource for studying the development of Empire-wide flows of people, goods, ideas and disease that were not merely structured or restricted to terrestrial means of transport.

## Methods

### Empire and province boundaries

The geographical limits of the road data and the analysis region are defined by the extent of the Roman Empire during the Antonine dynasty, ca. around 150 CE, when the Empire reached its largest extent. Provincial boundaries are based on provincial boundaries and Roman Empire extent by the year AD 200 available at the Ancient World Mapping Center^55^ and are only approximate. Provinces east of Euphrates were excluded, due to being occupied by the Roman Empire for short time periods and to being excluded from the road data collection^33^. All boundaries were clipped to modern coastlines. Inclusion of Italian *regiones* enhances the granularity of the results in the Italian peninsula. Unfortunately, a similar finer division is not entirely possible for other provinces.

### Analysis grid cells

Summary statistics for Roman roads, modern roads, modern population, mean elevation, mean TPI, road density, site density, and comparison between roads and sites were aggregated in 0.5° latitude x 0.5° longitude cells covering the entire research area. These cells were used as analytical units because they provide good commensurability of the data and results whilst still offering a fine-grained resolution reflective of the density of Roman roads. Cells without Roman and modern roads were excluded from the analysis.

### Modern roads data

The dataset of modern roads used in the article is a spatial subset of the World Roads dataset available at ArcGIS Online^56^. It is based on a world transportation dataset supplied by Garmin International, Ltd. and distributed by Esri. It contains modern major roads and highways, and selected ‘Local roads’ in certain areas. All are paved roads. Ferries were removed from the dataset for the analysis. Only roads overlapping with 0.5 degree cells were selected for analysis. This dataset was selected because it is open access, covers the entire research area, provides comparable data coverage across the more than 30 modern countries in the research area, and crucially because it represents a comparable level of road inclusion to the Itiner-e dataset since both focus on major connections between settlements, neither includes city streets, private roads, or local tracks.

### Ancient sites

The site density and spatial network models methods use locations of 14,317 ancient sites that intersect with our research area derived from the Pleiades: A Gazetteer of Past Places^57^ dataset with, according to the Pleiades ontology, the type values of ‘settlement’, ‘villa’, ‘fort’, and ‘station’ (referring to a road station), and time period ‘Roman’ (featureTyp LIKE ‘%settlement%‘ Or featureTyp LIKE ‘%villa%‘ Or featureTyp LIKE ‘%station%‘ Or featureTyp LIKE ‘%fort%‘ And timePeri_1 LIKE ‘%roman%‘).

### Ancient city population

The city centrality results use Hanson’s dataset^58^ of urban settlements in the Roman Empire, with associated population estimates (871 cities).

### Modern population

Modern population data are based on the LandScan Global 2022 population dataset^59^. The population values were summarised within 0.5° cells.

### Density

For both Roman and modern roads, kernel density was calculated in a 25 km neighbourhood over an area bounded by the extent of the 0.5° cells defining the research area. A smooth density surface is fitted over each road segment, with a density value of 1 where the surface overlaps with the highest values of the segment, and the density decreases away from the segment, reaching 0 at the specified neighbourhood limit of 25 km. No custom weighting or rescaling was applied. A 25 km neighbourhood was selected as approximately representing the average 1-day walking distance of a traveller. The kernel density was calculated using the ‘Kernel Density’ tool in ArcGIS Pro 3.2^60^.

### Comparison road and settlement density

Digital high-resolution demography data for the entire Roman Empire does not currently exist. Here we use the 14,317 sites described in ‘Ancient sites’, with approximately equal coverage and representativity across modern-day national states. A site density (sites/km^2^) was then calculated for each 0.5°cell. Provincial site density was calculated using the 13,638 sites within the provincial boundaries.

### Slope, TPI and sinuosity

We present results for three basic topographic properties of roads: slope (average and maximum), mean TPI (topographic position index), and sinuosity. All road segments are split into 1 km sections (resulting in 407,800 road segments), used as an analytical unit to enable comparison of results.

Slope is calculated along each segment of a line over a Digital Elevation Model (DEM). The length of the segment is defined by the resolution of the DEM (see below). Maximum slope is obtained from the segment with the largest value. Average slope is obtained by taking a weighted average of the slope from each line segment. Slope was calculated using the ‘Add Surface Information’ tool in ArcGIS Pro 3.2^61^ and then converted from percentages to degrees.

TPI^62,63^ is a measure of prominence of a raster cell in a DEM in a specified neighbourhood, calculated as a difference between the elevation of the focal cell and the mean elevation of its neighbourhood. Positive values indicate elevated areas, whereas negative values represent valleys. The TPI was calculated using the ‘Topography Toolbox’ for ArcGIS Pro 3.2^64^.

Sinuosity is a measure of the ‘straightness’ of the road. It is calculated as a ratio of actual road length to straight line distance between the start and end point of a 1 km road segment. The values closer to 1 indicate straight roads (with 1 being completely straight), higher values indicate roads with a more winding course. Sinuosity was calculated using ‘Stream Gradient’ and the ‘Sinuosity Toolbox’ for ArcGIS 10.1^65^

Both slope and Mean TPI were calculated over a Copernicus GLO-90 3-arcsec resolution DEM covering the whole research area. The Copernicus DEM was chosen because it offers better coverage in the mountainous areas and better vertical accuracy compared to SRTM or Aster GDEM data. Mean TPI was calculated using a 47 cell radius neighbourhood, which corresponds to ca. 5 km (the resolution of the DEM is ca. 107 m at the latitude of the research area). The 5 km neighbourhood was selected as we assume that local topography has a decisive influence on the location of the road.

### Topographic structure by certainty

8191.1 km of road data is certain (2.737%), 22,280.9 is hypothetical (7.445%) and 268,801.2 is conjectured (89.818%). Certainty categories and their implications for data confidence are discussed in detail in de Soto et al.^33^. ‘Certain’ indicates roads digitised with high spatial accuracy, ‘conjectured’ roads are digitised with lower spatial accuracy, and ‘hypothetical’ roads are those that were identified to exist but were not located or had a less fixed track.

Supplementary Fig. 1a, b captures variability of the average and maximum slope values between the certainty categories, showing that ‘Certain’ road segments (i.e., those digitised with high spatial accuracy) have slightly lower mean values and lower maximum values compared to ‘Conjectured’ segments (1.45° vs 1.54°, and 16.1° vs 31.2°). This might reflect the lower spatial accuracy of the conjectured segments since they might be located on unusually steep slopes. Supplementary Fig. 1c demonstrates that the TPI values are skewed towards slightly negative values among all certainty categories, comparable to the overall results for the roads. Supplementary Fig. 1d shows variability of the road sinuosity, where ‘Certain’ segments have slightly lower values compared to ‘Conjectured’ and ‘Hypothetical’ segments. As with the average slope values, this might reflect lower spatial accuracy of the ‘Conjectured’ and ‘Hypothetical’ segments.

### Slope, TPI and sinuosity by road type

For definitions of main and secondary roads, see de Soto et al.^33^. Supplementary Datasets 1–2 demonstrate that main roads tend to have lower mean slope and sinuosity values, but slightly higher TPI values. However, the differences are slight. When looking at both road type and segment certainty we see a similar picture, but the Certain road segments tend to have higher average slope values and Hypothetical segments tend to have TPI values closer to zero. This is likely caused by the fact that many certain segments are recorded in upland areas, on slopes and mountains. Hypothetical segments are then mostly found in the desert areas of North Africa and Syria in moderately flat areas (hence TPI values closer to zero).

### Orientation

Orientation was determined based on road segments from one intersection to the next intersection. All road segments in the dataset are split at intersections with other road segments. Segment orientation was classified into six direction categories covering 30° of the compass each: N-S, NNE-SSW, NE-SW, E-W, SE-NW, SSE-NNW (see Supplementary Table 8 for orientation classes in degrees). Only the start and end point of each segment is taken into account during the calculation, using the ‘Linear Directional Mean’ tool in ArcGIS Pro 3.2^66^. Predominant orientations of roads per province were calculated by aggregating the total length per orientation. To enable this, all roads going across provincial borders were cut at the borders. Road segments were excluded from the analysis if they go beyond the borders of the Empire (captured by the polygon describing provincial borders). Supplementary Dataset 3 provides a comparison with the predominant orientations of mountain ranges in mountainous provinces (aggregated by total area of the orientations) computed from the GMBA Mountain Inventory v2 dataset^67^.

### Network analysis

We represented the road system of the Empire overall and of individual provinces as planar undirected networks, with links representing road segments and nodes representing intersections and dead ends. To enable an analysis of the entire continental part of the Roman Empire, two artificial road connections were added at known ancient ferries^39^ across the Bosporus and Dardanelles that connect Europe to Asia Minor. As a result, the largest component spans Europe, the Middle East, and Africa, while smaller components include Great Britain and Mediterranean islands (see Fig. 4).

Supplementary Dataset 4 summarises essential spatial network properties of the Roman road networks and Supplementary Dataset 5 for major modern roads, both per Roman province^68^:

Gamma index^69^: the ratio $\frac{e}{3(n-2)}$ where e is the number of edges and ${n}$ is the number of nodes. 3(n−2) is the maximum possible number of edges in an undirected planar network with n nodes. It is an index of network density.

Alpha index^70^ of the largest connected component: the ratio $\frac{e-n+1}{2n-5},$ measuring the density of bounded faces in the largest connected component. It ranges between 0, for a tree, and 1, for a maximally connected planar network.

Additional network analysis measures were derived to enable comparison with non-spatial networks:

Global clustering coefficient: the number of closed triples over the total number of triplets (both open and closed).

Average degree**:** the average number of connections of a node.

### Travel time weighted edge betweenness

The edge betweenness centrality is the number of shortest paths between any two nodes in a network that include a given edge^38^. This measure can be interpreted as an indicator of the edge’s role in ensuring efficient connections in the network.

We computed the edge betweenness for the empire as a whole (Fig. 4b) as well as per province for all road segments that fall within a provinces’ borders (Fig. 4a), using the edge_betweenness function from the R package igraph (v.1.4.3)^71^. Different definitions of a shortest path can be employed; in our case, we defined it on the basis of the travel time to traverse each segment.

To estimate the travel time, we applied Tobler’s hiking formula Eq. (1)^72^. Let l be the length of a road segment in kilometres and let s be its average slope. The walking speed v in kilometres per hour is given by

1 $$v=6 \, \exp (-3.5{|s}+0.05|)$$

and the travel time in hours is calculated as t=l/v.

The data set classifies each road segment as either main or secondary (see de Soto et al.^33^). For each province, we computed the probability that a randomly selected main road has higher betweenness than a randomly selected secondary road. This provided a measure of the relative strategic importance of the two types of roads (Fig. 5a).

### Cities and node centrality

Supplementary Dataset 6 presents for each province’s road network the time-weighted betweenness, time-weighted closeness and the degree of the nearest node to a Roman urban settlement (see ‘Ancient city population’). Capital cities are more strongly skewed towards the top decile for betweenness and closeness than all cities are.

### Robustness of network analysis results

Since the road dataset is characterised by a degree of uncertainty and incompleteness, we tested the robustness of node degree and betweenness centrality, and of edge betweenness centrality with respect to random removal of uncertain road segments (i.e., those classified as either conjectured or hypothetical) using methods from refs. ^73,74^.

Supplementary Fig. 2 illustrates the effect of randomly removing uncertain road segments (i.e. those classified as ‘conjectured’ or ‘hypothetical’) with a 20% probability across 100 iterations, demonstrating robustness of the network results presented here. Supplementary Fig. 2a, b show that nodes with high degree are slightly less frequent in the randomised networks. For instance, the node with the highest degree (10) retains an average degree just above 8, never dropping below 5. The edge betweenness centrality remained larger for main roads than for secondary roads in all sampled random networks (Supplementary Fig. 2c). The betweenness of main roads is on average 1.95 times that of secondary roads, with a minimum ratio of 1.30 and a maximum of 3.01 across the random networks.

Supplementary Fig. 2d–i shows the centrality deciles for the closest nodes to cities for 100 test networks, each generated by randomly removing conjectured or hypothetical road segments with a 20% probability. The skewedness of capitals to the top 2 deciles of betweenness and closeness remarked in Fig. 5c–h persists in the test networks, indicating that the association between the locations of provincial capital cities and nodes with relatively high values of these centrality measures is robust with respect to the removal of uncertain road segments from the provincial road networks.

### Linear regression

We use 0.5° cells as analytical units (Fig. 2d), as well as Roman provinces (Supplementary Fig. 3). Within these units we summarised several relevant environmental and anthropic variables (see Supplementary Table 2). Both Roman and modern roads data were compared based on the area within a 5 km buffer along roads, an approach used in previous studies that enables comparison with previous results^25^. All polygons containing no Roman and modern roads were excluded from the analysis. A comparison of the percentage of these buffers occupying each analytical unit demonstrates only a small difference between Roman and modern roads. However, in the case of Roman provinces, the variation is more significant and it displays a different pattern with more variability than Roman roads (Supplementary Fig. 3a). We compute Pearson correlation to quantify the strength of a linear relationship between these variables. The results indicate a moderate positive linear correlation between Roman and Modern roads (for 0.5° cells a coefficient of approximately 0.460, *p* < 2.2e-16, and for provinces approximately 0.539, *p* = 1.541e-05). These results suggest that as the percentage of buffer area for Roman roads increases, there tends to be a corresponding increase in the percentage of buffer area for modern roads, and vice versa (Fig. 3d and Supplementary Fig. 3b; Supplementary Table 1).

### Spatial regression

We use Geographically Weighted Regression (GWR) analysis using 0.5° cells as analytical units, to explore in a spatial context the relationship between the key anthropic and environmental variables and the location of Roman roads, and the association of Roman roads and the location of modern roads. We first identify the level of spatial autocorrelation to evaluate spatial dependencies and clustering of the data using Moran’s I index. Given the resolution of the dataset, 100 km distance bands were selected. A field representing the percentage of the 5 km buffer around Roman roads in each polygon cell was used as input (see ‘Linear regression’). The results show the highest Moran’s I index in the first distance band (100 km) which sharply decreases at larger distances. However, as we evaluate z-values against the null hypothesis that observed phenomena are not spatially correlated, we see the most significant spatial autocorrelation around the 900 km distance band (Supplementary Table 3). Therefore, when selecting a neighbourhood for GWR the neighbourhood threshold was set at 1000 km. Both Moran’s I index and GWR analysis were undertaken using ArcGIS Pro 3 tools^75,76^. Notably high z-scores and therefore very low *p* values might be caused by the effect of a large sample size which decreases the variance of Moran’s I index and inflates the z-scores. Moreover, it implies very strong spatial dependencies in the dataset. However, since Moran’s I is a global diagnostic useful for global spatial models, it might not be the best indicator for defining a neighbourhood for a local GWR model. It was decided to compare GWR results using the 1000 km neighbourhood with adaptive neighbourhood using a Golden Search method to identify the optimal number of neighbours in order to find the model that better explains the variance and provides a better fit (using AICc and Adjusted R^2^ as a measure).

We explored three different scenarios with GWR (Fig. 3a–c; Supplementary Table 4): (1) whether Roman roads are associated with the presence of modern roads, (2) or the density of the modern population; and (3) to what degree mean elevation, the mean TPI calculated in a 5 km neighbourhood, or site density are associated with the location of the Roman roads. With exception of scenario 3, the models using the optimal number of neighbours method (Supplementary Table 4) provided better fit than the 1000 km neighbourhood (Supplementary Table 5). The 1000 km neighbourhood model at this scale tends to oversmooth local relationships and its results are closer to a global linear regression model (Fig. 3c and Supplementary Table 5, note very similar adjusted R^2^ values to the linear regression). The differences are most pronounced in scenario 1, where the model using the optimal number of neighbours shows strong spatial non-stationarity (i.e., the relationship between Roman and modern roads is spatially very varied). The following paragraphs describe the best performing models reported in the article (Supplementary Table 4). It shows that for scenario 1 and 2 the scale of coefficient non-stationarity is smaller than the dominant scale of spatial clustering.

Scenario 1 (Fig. 3a and Supplementary Table 4): the model shows pronounced spatial non-stationarity, the relationship between Roman roads and the location of modern roads varies widely across the study area. There is no contiguous region or area where the model shows good local fit. The model explains a large share of the variance (R^2^ = 0.69, adjusted 0.61), but the scale at which the relationship operates is small to medium, considering the optimal number of neighbours (31). We may surmise that the relationship between Roman and modern roads is heavily dependent on local influences rather than on transregional or continental scale circumstances e.g., institutional differences between various European regions or between Europe and North Africa and the Near East that shaped persistence and development of transport infrastructure, while local effects of historical and institutional development are more likely. Among other causes influencing the varied effect of Roman roads on modern roads is undoubtedly (a) topographical constraints and natural movement corridors, and (b) varied representativity and reliability of the dataset that influences the confidence in the reported results in certain regions (chiefly central Europe, Balkans, and marginal and (semi-)desert areas) (see ‘Robustness of spatial regression).

Scenario 2 (Fig. 3b; Supplementary Table 4): The model explains little of the variance (R^2^ values 0.39, adjusted 0.25) but while the modelled relationship is spatially smooth, it fails to capture the key drivers of the variation. It is only in regions with a very high density of population - mainly the major cities (i.e. London, Paris, Madrid, Rome, Milan, Naples, Istanbul, Ankara, etc.) and a few regions such as the Lower Rhine and the Nile Valley, where under- or overpredictions are present. We may suggest that in this scenario, our results may be biased by modern development around contemporary major urban areas, with more investigations and higher recovery rates of archaeological remains providing detailed data on the Roman road network compared to other areas (e.g., rural Spain, western Balkans, etc.). But substantively, Roman roads are not very well associated with modern population density.

Scenario 3 (Fig. 3c and Supplementary Table 4): we explored the spatial relationship between Roman roads and several environmental and anthropic variables, which were selected to help us understand where they had the most decisive influence on the formation of the Roman road network. A global adjusted R^2^ value of 0.35 (0.5-degree cells) suggests a rather low fit of the model when using a limited number of chosen variables. This might be explained by poor coverage of the road dataset in certain regions (south-western France, upper Danube, central-south Balkans, central Italy, Sicily, Corsica) or by underrepresentation/overrepresentation of site data in the Pleiades dataset (coast of the Near East and North Africa, see ‘Ancient sites’). Additional environmental factors, especially related to climate, precipitations and soil quality might become helpful in marginal desert and mountainous environments.

### Robustness of spatial regression

We can assess the effect of the neighbourhood size by comparing with the GWR model using a 1000 km neighbourhood. Scenarios 2 and 3 (Supplementary Table 5) do not substantially differ when using either a 1000 km neighbourhood or optimal number of neighbours. In scenario 1 (Supplementary Fig. 4, Supplementary Table 5) the large neighbourhood tends to oversmooth the results and so the performance of the model is close to the non-weighted linear regression. The pattern of model residuals is locally varied and the presence of Roman roads does not have strong explanatory power on the location of modern roads, as in the case of the GWR model using a smaller neighbourhood. The global R^2^ value (0.47, adjusted 0.46) implies that the model explains a substantial share of the variance but the relationship between the two is more complex. The regions of overprediction (using standardised residuals) show interesting patterns. They cover the former Roman frontier in Europe, along the Rhine and most of the Danube. This may suggest that the sustained presence of the Roman army on the borders of the Roman Empire and investment in developing the road system, might have served as a framework for later development, and may be a process driving road persistency. Among other areas where we can observe high model residuals are regions around large modern agglomerations (London, Paris, Madrid, Marseille, Milan, Istanbul, Athens, Cairo, etc.). Extensive construction and improvements of the modern road network for the growing major cities could bias the model in such cases. In other instances, the high residuals might be caused by geography which was the major factor in defining settlement patterns and movement (the Nile Valley, Cyrenaica and Tripolitania in Libya, Israel, Lebanon, and Palestinian Territories). However, this pattern cannot be observed e.g., in the Alps or the Taurus Mountains, which require different explanations. In yet another instance, high residuals could be explained by both geography and the presence of modern major cities (e.g. Madrid, Ankara). We may observe a particularly telling pattern in the Iberian Peninsula. The highest residuals are found on the coast and in the interior around Madrid. This corresponds well with previous conclusions^22^ on the historical development of the road network in Spain, where centralisation tendencies focusing on Madrid resulted in a higher concentration of population and the development of a road network radially extending from Madrid to the detriment of the rest of the interior of Spain. Therefore, outside of Madrid, the model shows neutral or slightly negative residuals.

We compared the GWR results obtained for 0.5° cells with results obtained for a finer resolution dataset composed of 0.25° cells (Supplementary Fig. 5, Supplementary Table 6). We used the same Golden Search method to identify the optimal number of neighbours. Surprisingly, the models for scenarios 1 and 2 produced at 0.25° resolution performed worse than models obtained for 0.5° polygons both regarding global and adjusted R^2^ values. However, all 3 scenarios essentially reveal the same patterns (or lack thereof) as the coarser resolution models. In scenario 2, more places of over- and underpredictions are identified, but these still represent major modern urban centres, and so the issue of representativity of the road dataset with regards to recovery of archaeological data in modern cities remain. Only in scenario 3, the global and adjusted R^2^ values slightly improve, thus explaining more variance (0.4 vs. 0.35), implying that elevation, TPI and Roman settlement patterns have stronger a influence on the location of Roman roads at a small scale.

We compare the GWR results with the representativity and reliability provided for the Itiner-e dataset^33^. Representativity quantifies how the density and spatial detail of the dataset is representative with regard to the global Roman road density average. Reliability expresses reliability of the sources used for creation of the dataset. We overlay these categories over the GWR results for all 0.5° cells that have both Roman and modern roads (Supplementary Fig. 7). The definition of the categories was selected to highlight the differences between low to negative standardised residuals (<0.5) and high standardised residuals (>0.5), and low representativity (‘Low’ category in de Soto et al. (2025)), and high representativity (categories ‘Average’, ‘Above Average’, and ‘Exceptional’ in de Soto et al. (2025)). The reliability categories (‘Low’, ‘Medium’, ‘High’) were not changed. It shows regions where we have higher confidence in the results (high representativity and medium to high reliability) in north-western Europe, Spain, Italy, Greece, the eastern Balkans, most of Asia Minor, the Near East, the Eastern Desert of Egypt, coastal Cyrenaica and Tripolitania, and North Africa. Problematic areas (low representativity and low reliability) are found mostly in central Europe, the western Balkans, the Nile valley, marginal semi- and desert areas, and Corsica. However, poor results for the western Balkans are caused mainly by low reliability of sources, while the representativity of the data is high and therefore the residuals shown by GWR are likely robust and comparable to high confidence regions. The most problematic region is then central Europe roughly from the upper Rhine to Pannonia, which consistently exhibits low representativity and low reliability. The same applies to semi-desert and desert areas, but it is unlikely that the Roman road system there was more extensive, and therefore GWR results should hold. Areas of low representativity and medium reliability, covering peripheral regions of Britain, Portugal, Switzerland, Sardinia, parts of the Alps and Italy, central Anatolia, and most of Egypt, are places where the results might be potentially flawed, due to underrepresentation of roads in the dataset. We provide a further robustness check by re-creating scenario 1 using only 0.5° polygons that have either ‘Medium’ or ‘High’ reliability and ‘Average’ to ‘Exceptional’ representativity. The model (Supplementary Fig. 6 and Supplementary Table 7) leads to comparable results, although it has slightly lower R^2^ values (0.59, adjusted 0.53).

### Reporting summary

Further information on research design is available in the Nature Portfolio Reporting Summary linked to this article.

## Supplementary information

Supplementary Information

Description of Additional Supplementary Files

Supplementary Dataset 1-7

Reporting Summary

Transparent Peer Review file

## Data Availability

Roman roads dataset: the version of the Itiner-e dataset used to perform this analysis is available as a polyline vector layer in the shapefile format (itinere_roads.shp), geopackage (itinere_roads.gpkg) and as a geojson database (itinere_roads.geojson) on the Zenodo repository^77^. A growing gazetteer version of the dataset can be viewed and queried on the Itiner-e linked open data platform (https://itiner-e.org)^78^. The creation of the dataset is described in de Soto et al.^33^. The version of the road dataset split into 1000 m long segments with calculated topographic variables, the polygons representing various analytical units (provinces, 0.5° and 0.25°cells) containing topographic and anthropic variables, and additional data generated with analytical tools and used in this article are stored at a Zenodo repository^79^. Modern roads: World Roads dataset available at ArcGIS Online^56^. Provincial boundaries: Roman Empire extent by the year AD 200 available at the Ancient World Mapping Center^55^. Ancient site locations: Pleiades: A Gazetteer of Past Places^57^. City population estimates: Hanson Cities Database (OXREP)^58,80,81^. Modern population data: LandScan Global 2022 population dataset^59^.

## Code availability

The spatial datasets used and the code to reproduce the network analysis results is available at: https://github.com/MatteoMazzamurro/roman-road-networks^82^. Code and sources for the linear regression analysis are available here: https://github.com/Mcotsar/Ancient_Roads_Exploratory_Analysis
