# Supplementary material for: Network science reveals the structure and lasting impact of the Roman road system
Source: Nat Commun. 2026 Sep 15;17:9551. doi: 10.1038/s41467-026-75453-3 (PMC13578412; doi:10.1038/s41467-026-75453-3)
Supplement: Supplementary file 1 [file 41467_2026_75453_MOESM1_ESM.pdf]

Supplementary Information for

Network Science reveals the structure and lasting impact of the Roman road system

Adam Pažout, Tom Brughmans, Pau de Soto, Matteo Mazzamurro, María Coto-Sarmiento, Clara Filet, Eduardo Herrera Malatesta, Magnus L. Nielsen, Gustav Emil Ølgaard, Peter B. Vahlstrup

Corresponding author: Tom Brughmans, t.b@cas.au.dk

This PDF file includes:

Supplementary Figures 1 to 8
Supplementary Tables 1 to 8

Other supplementary materials for this manuscript include the following datasets:

Supplementary Datasets 1 to 7

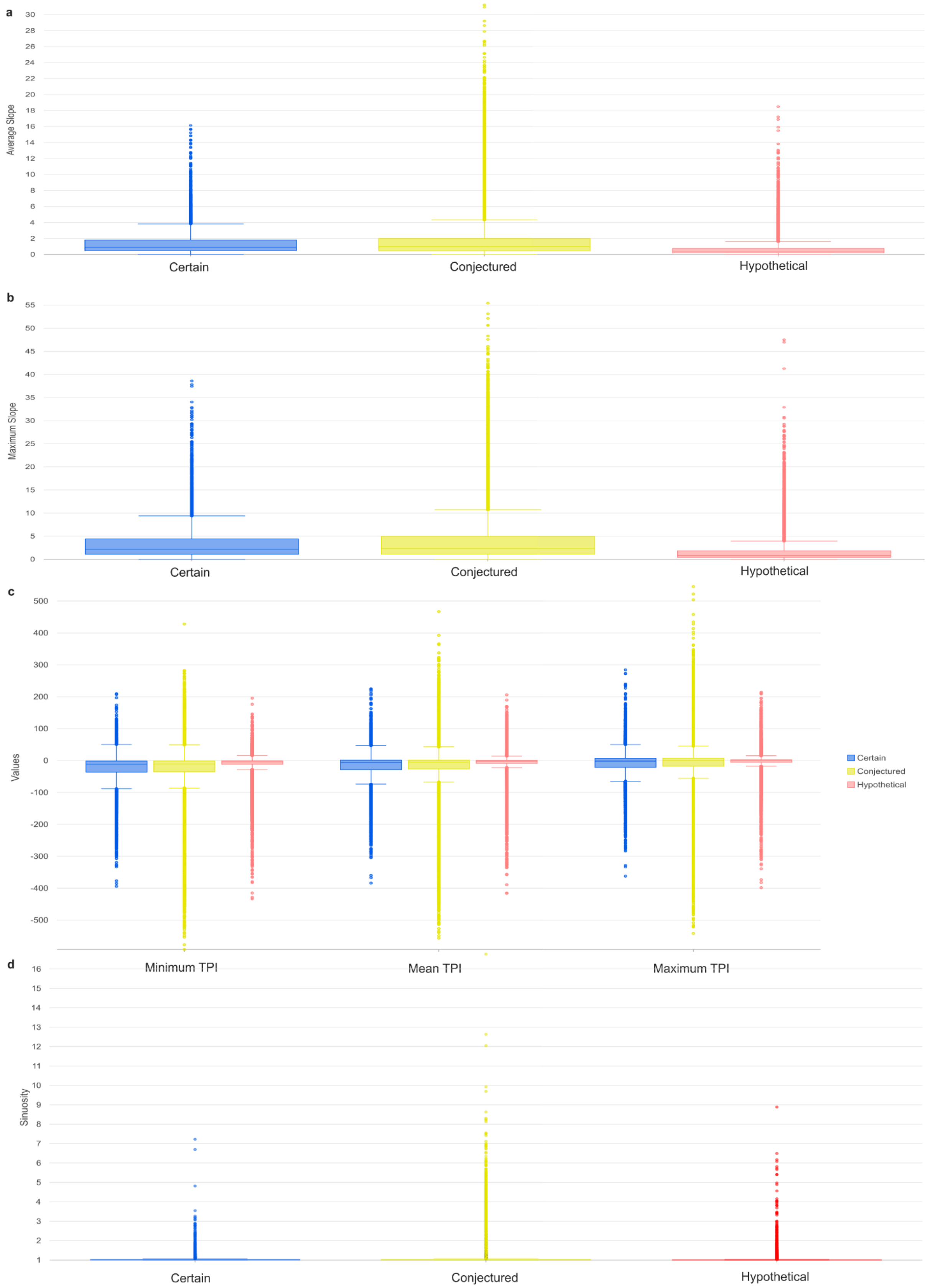

Supplementary Fig. 1. Topographical variables by certainty categories. **a**, average slope. **b**, maximum slope. **c**, minimum, mean, and maximum Topographic Position Index. **d**, sinuosity.

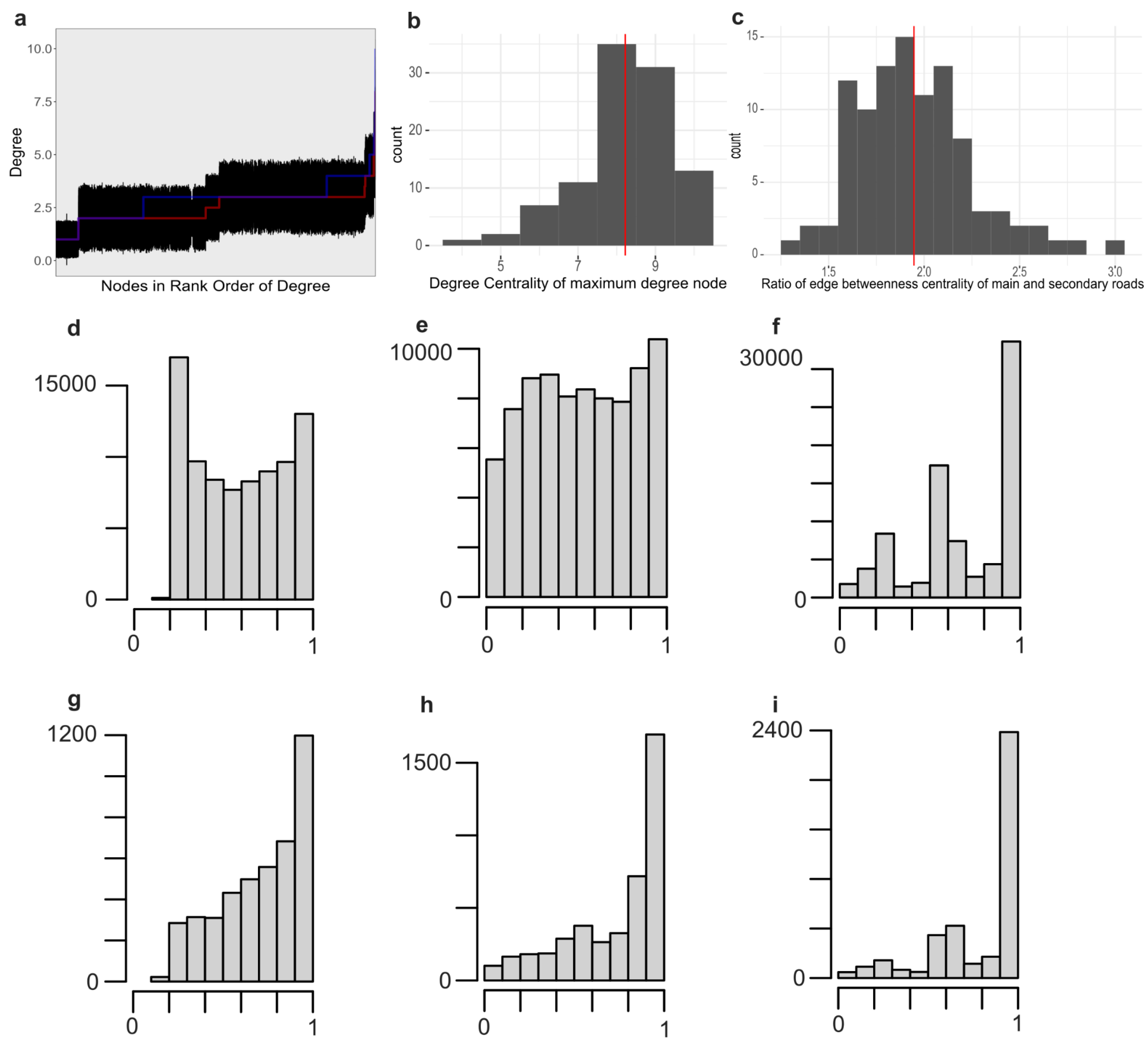

Supplementary Fig. 2. Impact on network metrics of randomly removing uncertain road segments with a 20% probability. **a**, the degree of nodes in the original network (blue line) and the median degree centrality across 100 random networks generated by removing uncertain road segments with a 20% probability (red line). The impact of edge removal on the degree distribution is modest. **b**, Histogram showing how the degree centrality of the node with the highest degree in the original network is affected by the random removal of road uncertain segments. The vertical line marks the mean degree centrality of this node across all random iterations. **c**, histogram of the ratio of edge betweenness centrality between main and secondary roads across the 100 random networks. The red line represents the mean value. The edge betweenness of main roads consistently exceeds that of secondary roads. **d-f**, histograms of centrality deciles for closest nodes to non-capital cities across 100 random networks. **g-i** histograms of centrality deciles for closest nodes to capital cities across 100 random networks. Capitals are skewed to the top 2 deciles of betweenness and closeness.

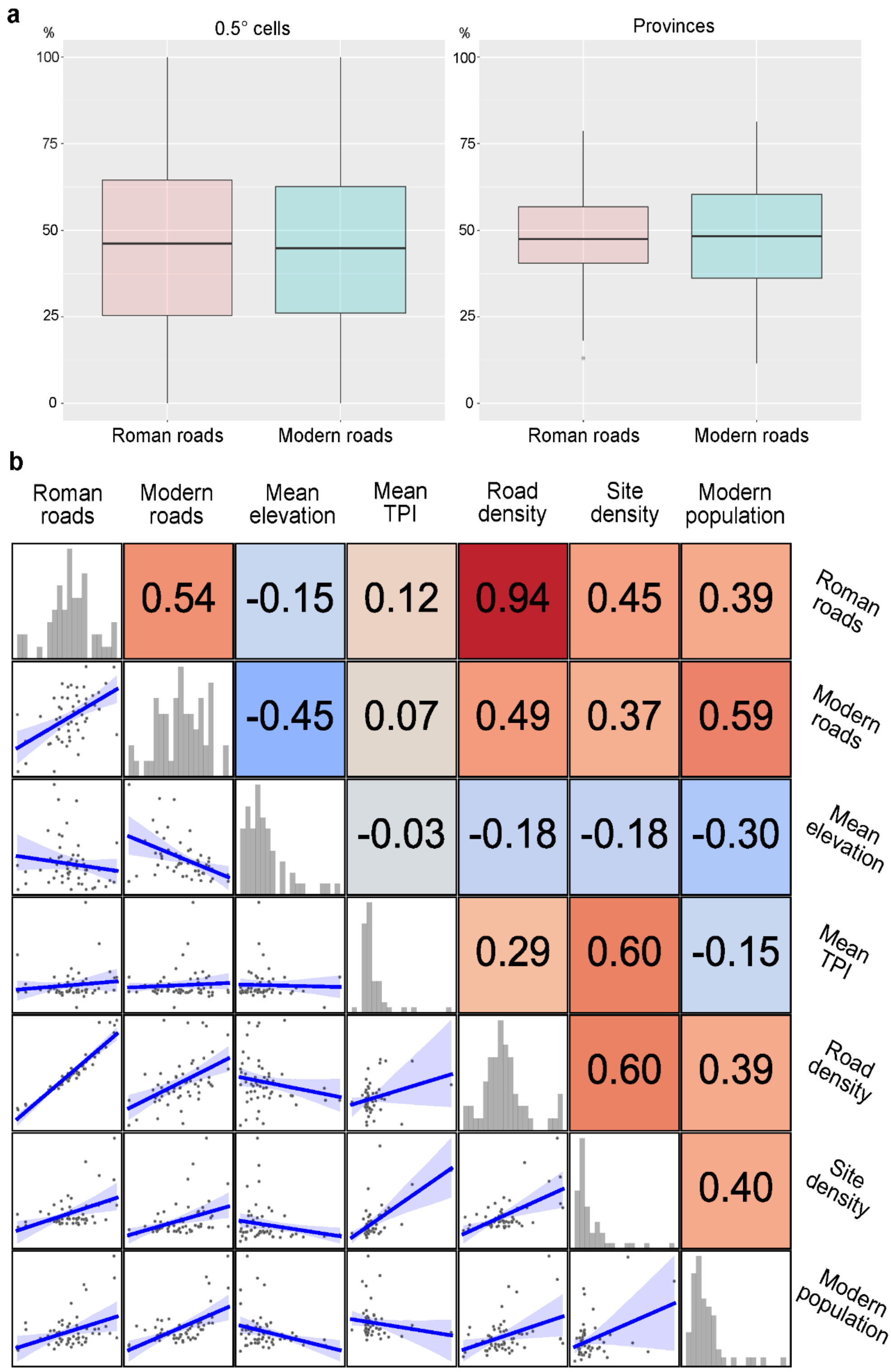

Supplementary Fig 3. **a**, Boxplot comparison data calculating the percentage of the buffers extracted for Roman roads and modern roads between 0.5° cells (basic unit of analysis) and Roman provinces. **b**, linear regression for topographic and anthropic variables summarized for provinces, lower left: scatterplots showing 95% confidence intervals of the fitted regression line, diagonal: histograms, upper right: Pearson's ρ values.

Supplementary Table 1. Results of the Generalized Linear Regression using 0.5° cell polygons. Values with asterisk signify statistically significant results (*p*<0.01). Coefficient: the strength and type of relationship between each explanatory variable and the dependent variable. VIF: Variance Inflation Factor, large VIF (> 7.5) indicate redundancy among explanatory variables.

Summary of GLR Results [Model Type: Continuous (Gaussian/OLS)]								
Variable	Coefficient	StdError	t-Statistic	Probability	Robust_SE	Robust_t	Robust_Pr	VIF
Intercept	23.287507	1.199908	19.407751	0.000000*	1.183123	19.683082	0.000000*	--------
MAREAPRC	0.438232	0.019680	22.267498	0.000000*	0.023341	18.774939	0.000000*	1.213506
MEANELEV	0.001966	0.001143	1.719799	0.085602	0.001049	1.873418	0.061126	1.104521
MEANTPI	-0.241091	0.145196	-1.660454	0.096960	0.239397	-1.007077	0.313983	1.040476
SITEDENS	335.683212	36.250917	9.259992	0.000000*	154.027314	2.179375	0.029381*	1.078651
MPOPDENS	0.000766	0.000925	0.828388	0.407516	0.000799	0.959427	0.337422	1.120371

GLR Diagnostics			
Input Features	05deg_cell_nonnull_stats	Dependent Variable	RAREAPRC
Number of Observations	2507	Akaike's Information Criterion (AICc)	22744.498860
Multiple R-Squared	0.237495	Adjusted R-Squared	0.235971
Joint F-Statistic	155.796068	Prob(>F). (5.2501) degrees of freedom	0.000000*
Joint Wald Statistic	621.850072	Prob(>chi-squared). (5) degrees of freedom	0.000000*

Koenker (BP) Statistic	363.294014	Prob(>chi-squared). (5) degrees of freedom	0.000000*
Jarque-Bera Statistic	54.685246	Prob(>chi-squared). (2) degrees of freedom	0.000000*

Supplementary Table 2. List of variables used for the exploratory analysis.

Variable	Description
landArea	Total land area of a polygon in square kilometers
romanArea	Area of a 5 km buffer along Roman roads in a polygon
modArea	Area of a 5 km buffer along Modern roads in a Polygon
rAreaPrc	Percentage of the total polygon area covered by a 5 km buffer around Roman roads
mAreaPrc	Percentage of the total polygon area covered by a 5 km buffer around Modern roads
meanElev	Mean elevation in the whole polygon
meanTpi	Mean TPI in the whole polygon calculated in a 5 km neighbourhood
roadLength	Length of Roman roads in a polygon
roadDens	Density of Roman roads in a polygon
siteCount	Number of Roman sites in a polygon
siteDens	Density of the sites in a polygon (sites/km^2)

modernPop	Modern population
mPopDens	Modern population density

Supplementary Table 3. Moran’s I index for the 5 km buffer around Roman roads in 0.5° cell units. Moran’s I was calculated for 100 km distance bands.

Distance band (m)	Moran's Index	Expected Index	Variance	z-score	*p*-value
100,000	0.446398	-0.000399	0.000126	39.817237	<0.000001
200,000	0.345045	-0.000399	0.000034	59.287036	<0.000001
300,000	0.278055	-0.000399	0.000016	68.950913	<0.000001
400,000	0.232556	-0.000399	0.00001	74.987103	<0.000001
500,000	0.194774	-0.000399	0.000007	76.420756	<0.000001
600,000	0.173586	-0.000399	0.000005	80.711268	<0.000001
700,000	0.155556	-0.000399	0.000004	83.163074	<0.000001
800,000	0.142264	-0.000399	0.000003	85.679041	<0.000001
900,000	0.129463	-0.000399	0.000002	86.494348	<0.000001
1,000,000	0.115582	-0.000399	0.000002	84.553077	<0.000001
1,100,000	0.101222	-0.000399	0.000002	80.827013	<0.000001
1,200,000	0.086621	-0.000399	0.000001	75.200667	<0.000001
1,300,000	0.07226	-0.000399	0.000001	68.014646	<0.000001

1,400,000	0.06085	-0.000399	0.000001	61.955	<0.000001
1,500,000	0.05215	-0.000399	0.000001	57.207615	<0.000001
1,600,000	0.045775	-0.000399	0.000001	54.098695	<0.000001
1,700,000	0.040654	-0.000399	0.000001	51.680049	<0.000001
1,800,000	0.037119	-0.000399	0.000001	50.641186	<0.000001
1,900,000	0.034518	-0.000399	<0.000001	50.438593	<0.000001
2,000,000	0.032822	-0.000399	<0.000001	51.26374	<0.000001
2,100,000	0.031098	-0.000399	<0.000001	51.923948	<0.000001
2,200,000	0.029199	-0.000399	<0.000001	52.039246	<0.000001
2,300,000	0.027106	-0.000399	<0.000001	51.532894	<0.000001
2,400,000	0.02529	-0.000399	<0.000001	51.238394	<0.000001
2,500,000	0.02312	-0.000399	<0.000001	49.868172	<0.000001
2,600,000	0.020751	-0.000399	<0.000001	47.67556	<0.000001
2,700,000	0.018985	-0.000399	<0.000001	46.42164	<0.000001
2,800,000	0.017687	-0.000399	<0.000001	45.978462	<0.000001
2,900,000	0.016321	-0.000399	<0.000001	45.07954	<0.000001
3,000,000	0.015053	-0.000399	<0.000001	44.155853	<0.000001

Supplementary Table 4. Geographically Weighted Regression model statistics results for 0.5° cell analytical units visualising the relationship between Roman roads and modern roads (scenario 1), modern population density (scenario 2), mean elevation, mean Topographic Position Index, and site density (scenario 3). These are the best performing models discussed in the article.

Scenario 1: modern roads	
Number of Features	2507
Dependent Variable	mAreaPrc
Explanatory Variables	rAreaPrc
Number of Neighbours	31
Model Diagnostics	
R^2	0.6979
AdjR^2	0.6111
AICc	21309.1604
Sigma-Squared	247.0776
Sigma-Squared MLE	191.9818
Effective Degrees of Freedom	1947.9645
Adjusted Critical Value of Pseudo-t Statistics	3.6805
Scenario 2: modern population	
Number of Features	2507
Dependent Variable	mPopDens
Explanatory Variables	rAreaPrc

Number of Neighbours	34
Model Diagnostics	
R^2	0.3974
AdjR^2	0.2536
AICc	38005.0962
Sigma-Squared	198138.0719
Sigma-Squared MLE	159972.0276
Effective Degrees of Freedom	2024.0929
Adjusted Critical Value of Pseudo-t Statistics	3.6419
Scenario 3: elevation, TPI, site density	
Number of Features	2507
Dependent Variable	rAreaPrc
Explanatory Variables	meanElev, meanTpi, siteDens
Distance Band (km)	1,000
Model Diagnostics	
R^2	0.3662
AdjR^2	0.3482
AICc	22378.0474
Sigma-Squared	433.5603
Sigma-Squared MLE	421.5677

Effective Degrees of Freedom	2437.6546
Adjusted Critical Value of Pseudo-t Statistics	2.9015

Supplementary Table 5. Geographically Weighted Regression model statistics results for 0.5° cell analytical units visualising the relationship between Roman roads and modern roads (scenario 1), modern population density (scenario 2), mean elevation, mean Topographic Position Index, and site density (scenario 3). These are the models using 1,000 km neighbourhood for scenarios 1-2 and optimal number of neighbours for scenario 3, that perform worse than the models presented in the article.

Scenario 1: modern roads	
Number of Features	2507
Dependent Variable	mAreaPrc
Explanatory Variables	rAreaPrc
Distance Band (km)	1000
Model Diagnostics	
R^2	0.4723
AdjR^2	0.4625
AICc	21764.3168
Sigma-Squared	341.5476
Sigma-Squared MLE	335.2843
Effective Degrees of Freedom	2461.0264
Adjusted Critical Value of Pseudo-t Statistics	2.9793
Scenario 2: modern population	

Number of Features	2507
Dependent Variable	mPopDens
Explanatory Variables	rAreaPrc
Distance Band (km)	1000
Model Diagnostics	
R^2	0.1206
$AdjR^2$	0.1041
AICc	38174.6921
Sigma-Squared	237829.4121
Sigma-Squared MLE	233468.0728
Effective Degrees of Freedom	2461.0264
Adjusted Critical Value of Pseudo-t Statistics	2.9793
Scenario 3: elevation, TPI, site density	
Number of Features	2507
Dependent Variable	rAreaPrc
Explanatory Variables	meanElev, meanTpi, siteDens
Number of Neighbours	1000
Model Diagnostics	
R^2	0.2311
$AdjR^2$	0.2233

AICc	22792.8774
Sigma-Squared	516.6367
Sigma-Squared MLE	511.4487
Effective Degrees of Freedom	2481.8248
Adjusted Critical Value of Pseudo-t Statistics	2.5706

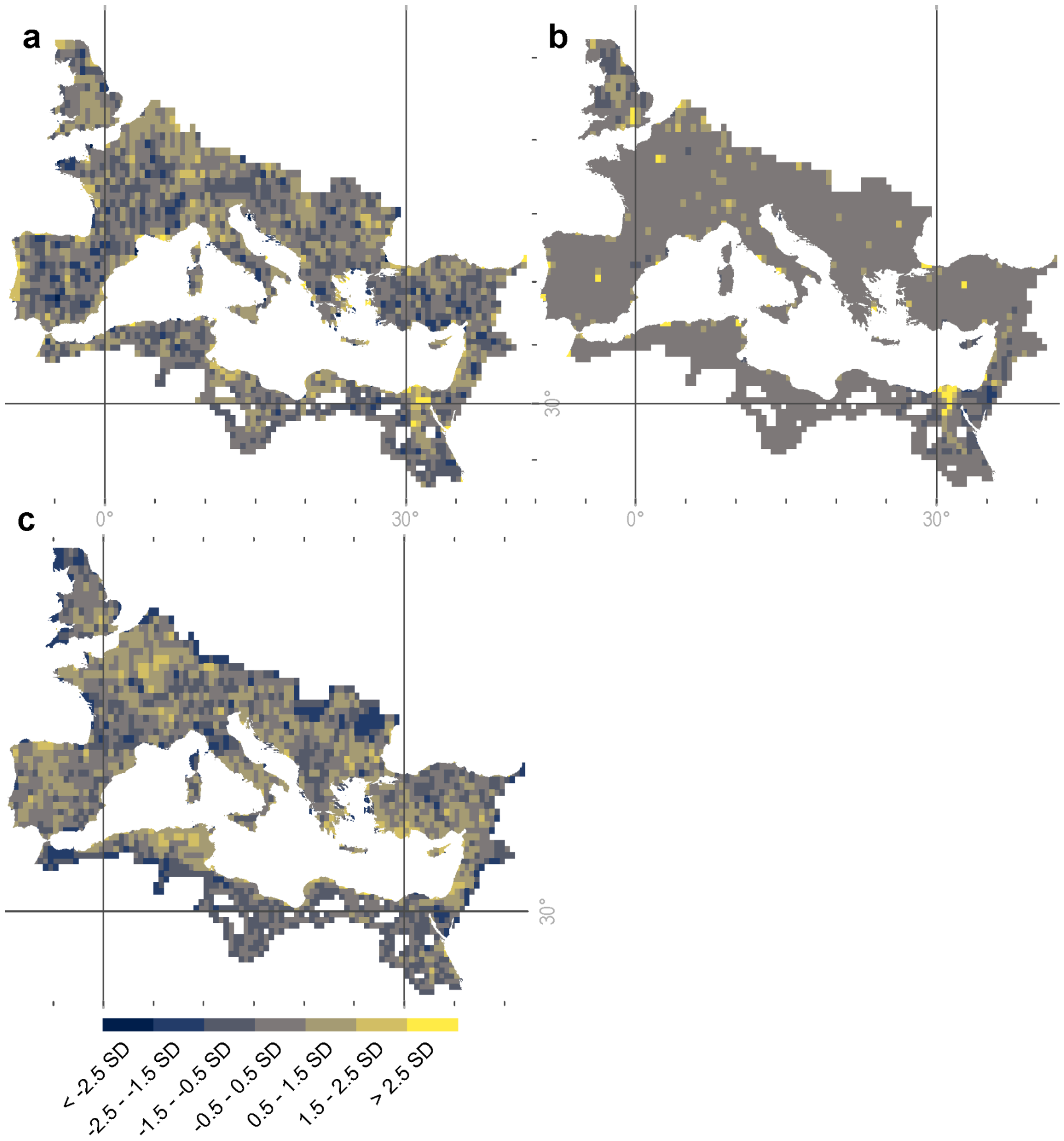

Supplementary Fig. 4. Geographically Weighted Regression results for scenario 1 (a) and scenario 2 (b), using a 1,000 km neighbourhood and for scenario 3 (c) using optimal number of neighbours.

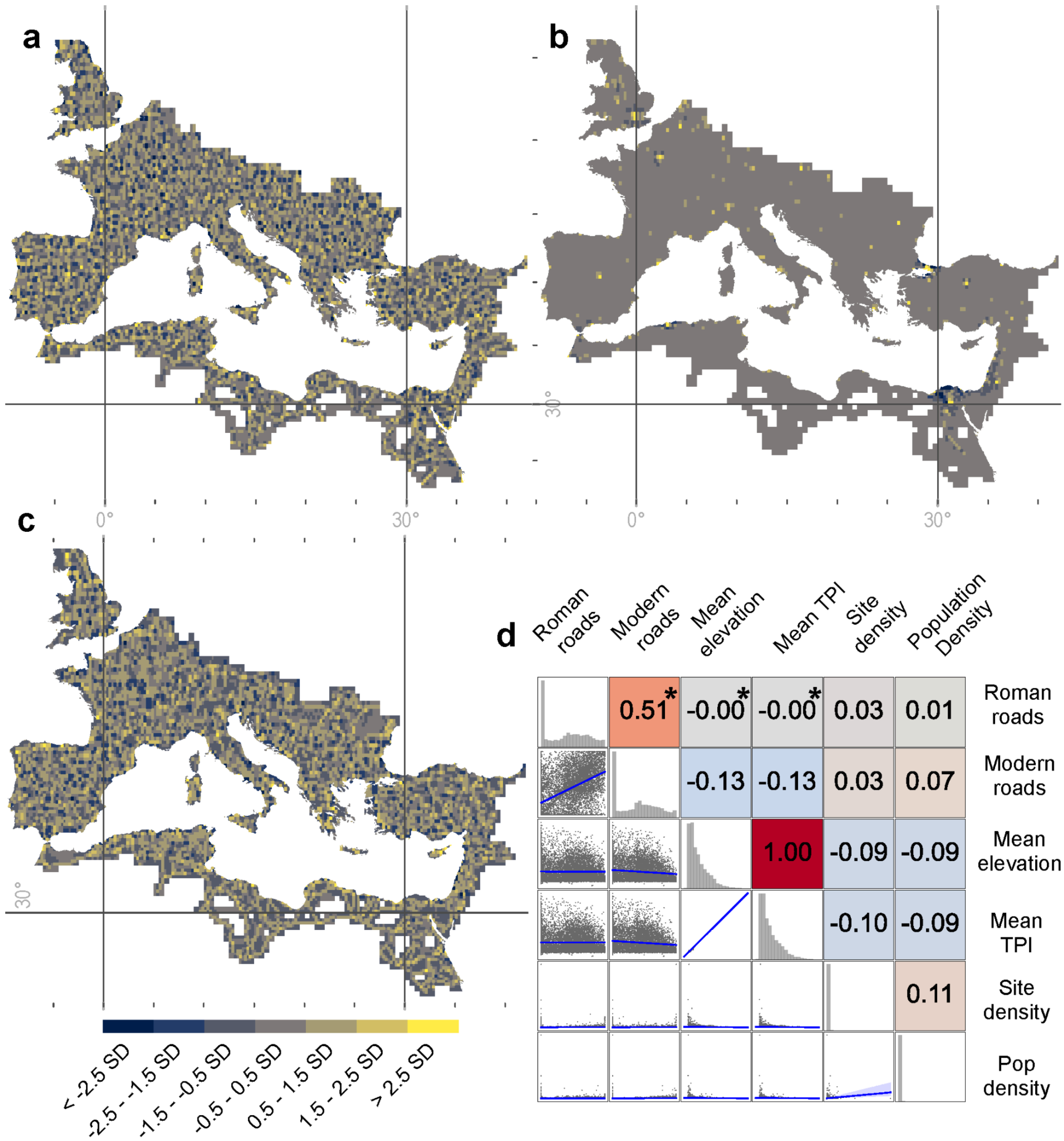

Supplementary Fig. 5. Geographically Weighted Regression analysis for 0.25° cells. **a**, scenario 1: relationship between Roman and modern roads. **b**, scenario 2: relationship between Roman roads and modern population. **c**, scenario 3: relationship between Roman roads and topographic and anthropic variables (mean elevation, mean Topographic Position Index, site density). **d**, linear regression of all dependent and explanatory variables, lower left: scatterplots showing linear trend, diagonal: histograms, upper right: Pearson's ρ values. Asterisk shows a statistically significant relationship (p-value < 0.05).

Supplementary Table 6. Geographically Weighted Regression model statistics results for 0.25° cell analytical units visualising the relationship between Roman roads and modern roads (scenario 1), modern population density (scenario 2), mean elevation, mean Topographic Position Index, and site density (scenario 3). All models use the optimal number of neighbours.

Scenario 1: modern roads	
Number of Features	9417
Dependent Variable	mAreaPrc
Explanatory Variables	rAreaPrc
Number of Neighbours	49
Model Diagnostics	
R^2	0.6009
AdjR^2	0.5334
AICc	84871.8472
Sigma-Squared	441.4987
Sigma-Squared MLE	377.5922
Effective Degrees of Freedom	8053.8979
Adjusted Critical Value of Pseudo-t Statistics	3.8950
Scenario 2: modern population	
Number of Features	9417
Dependent Variable	mPopDens

Explanatory Variables	rAreaPrc
Number of Neighbours	69
Model Diagnostics	
R^2	0.2104
$AdjR^2$	0.1198
AICc	158072.7120
Sigma-Squared	1079377.0684
Sigma-Squared MLE	968337.1563
Effective Degrees of Freedom	8448.2349
Adjusted Critical Value of Pseudo-t Statistics	3.8101
Scenario 3: elevation, TPI, site density	
Number of Features	9417
Dependent Variable	rAreaPrc
Explanatory Variables	meanElev, meanTpi, siteDens
Number of Neighbours	111
Model Diagnostics	
R^2	0.4648
$AdjR^2$	0.4028
AICc	87538.4099
Sigma-Squared	599.8888

Sigma-Squared MLE	537.6411
Effective Degrees of Freedom	8439.8412
Adjusted Critical Value of Pseudo-t Statistics	3.6435

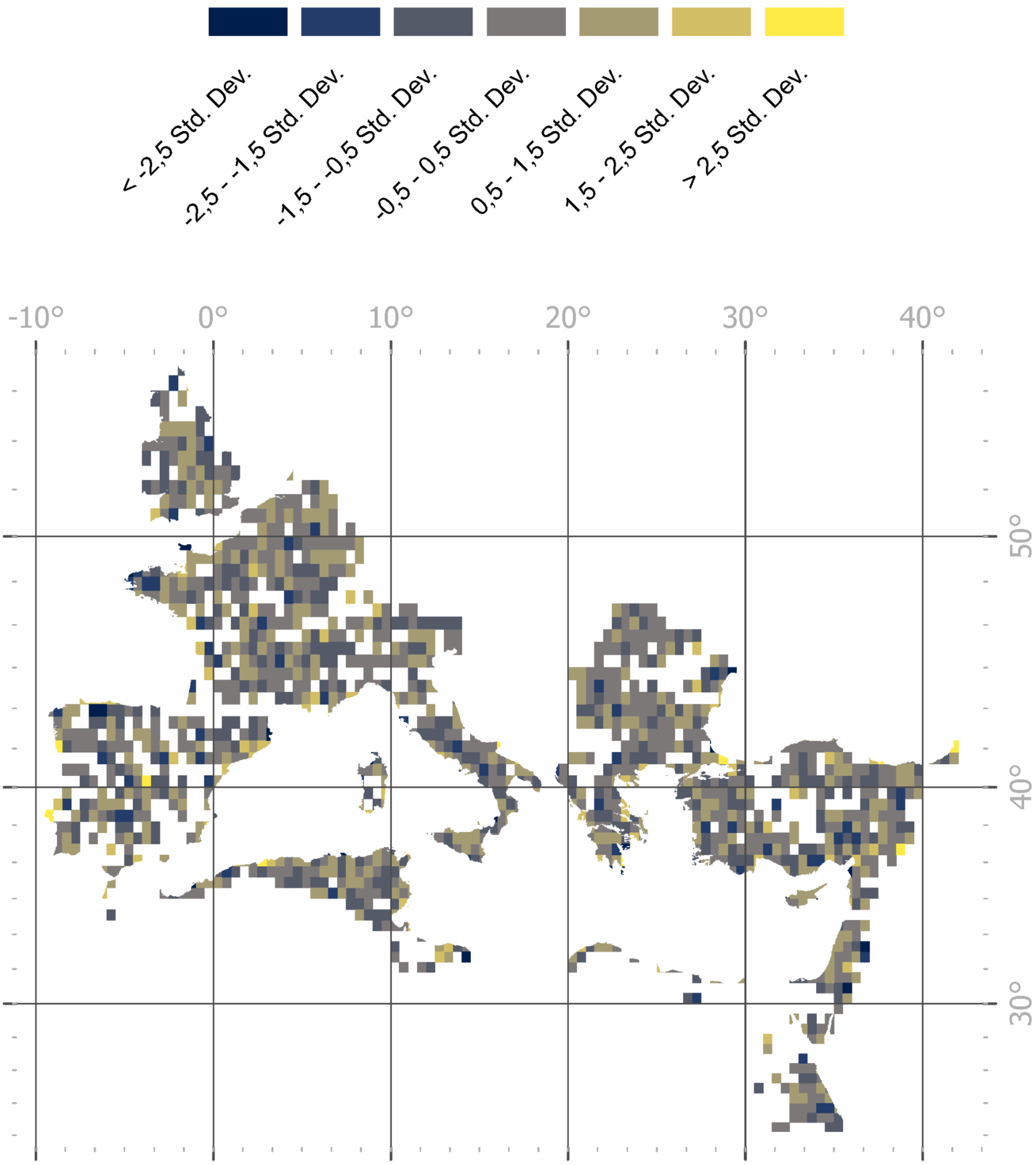

Supplementary Fig. 6. Geographically Weighted Regression analysis for 0.5° cells. Scenario 1 using only cells with ‘Average’ to ‘Exceptional’ representativity and ‘Medium’ to ‘High’ reliability.

Supplementary Table 7. Geographically Weighted Regression model statistics results for 0.5° cell analytical units visualising the relationship between Roman roads and modern roads using only cells with ‘Average’ to ‘Exceptional’ representativity and ‘Medium’ to ‘High’ reliability.

Scenario 1: modern roads	
Number of Features	1310
Dependent Variable	mAreaPrc
Explanatory Variables	rAreaPrc
Number of Neighbours	44
Model Diagnostics	
R^2	0.5958
$AdjR^2$	0.5250
AICc	11050.2628
Sigma-Squared	245.4774
Sigma-Squared MLE	208.9231
Effective Degrees of Freedom	1114.9265
Adjusted Critical Value of Pseudo-t Statistics	3.4061

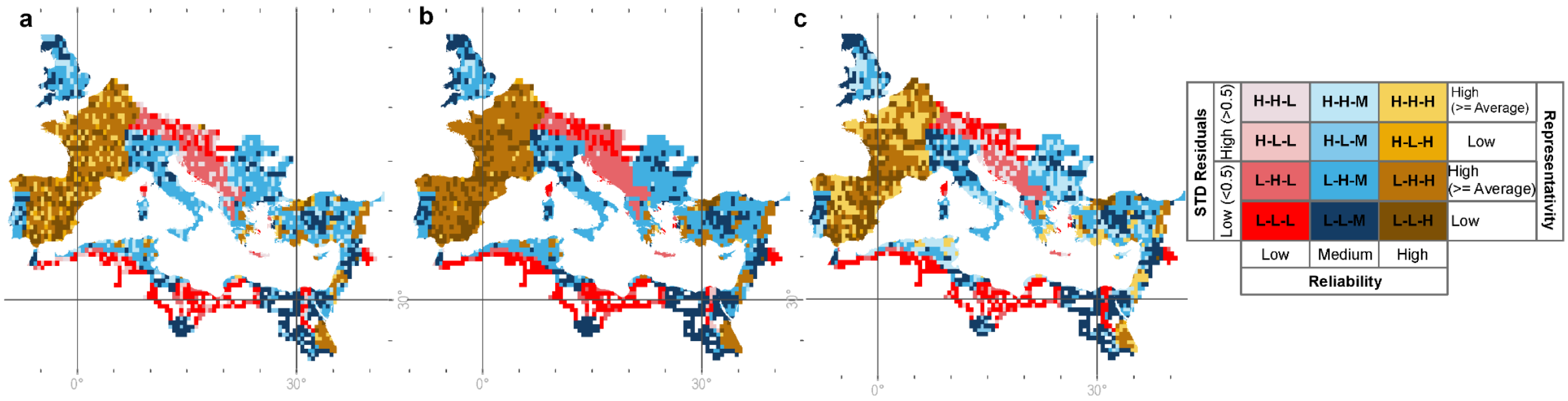

Supplementary Fig. 7. Confidence in the results of the Geographically Weighted Regression analysis. **a**, scenario 1 (Roman and modern roads). **b**, scenario 2 (Roman roads and modern population density). **c**, scenario 3 (Roman roads and topographic and anthropic variables.

Supplementary Table 8. Delimitation of orientation classes used in the article. Azimuth degrees with 0° indicating true north.

North - South (N-S)	345°-15°/165°-195°
North-North East - South-South West (NNE-SSW)	15°-45°/195°-225°
North East - South West (NE-SW)	45°-75°/225°-255°
East-West (E-W)	75°-105°/255°-285°
South East - North West (SE-NW)	105°-135°/285°-315°
South-south East - North-north West (SSE-NNW)	135°-165°/315°-345°

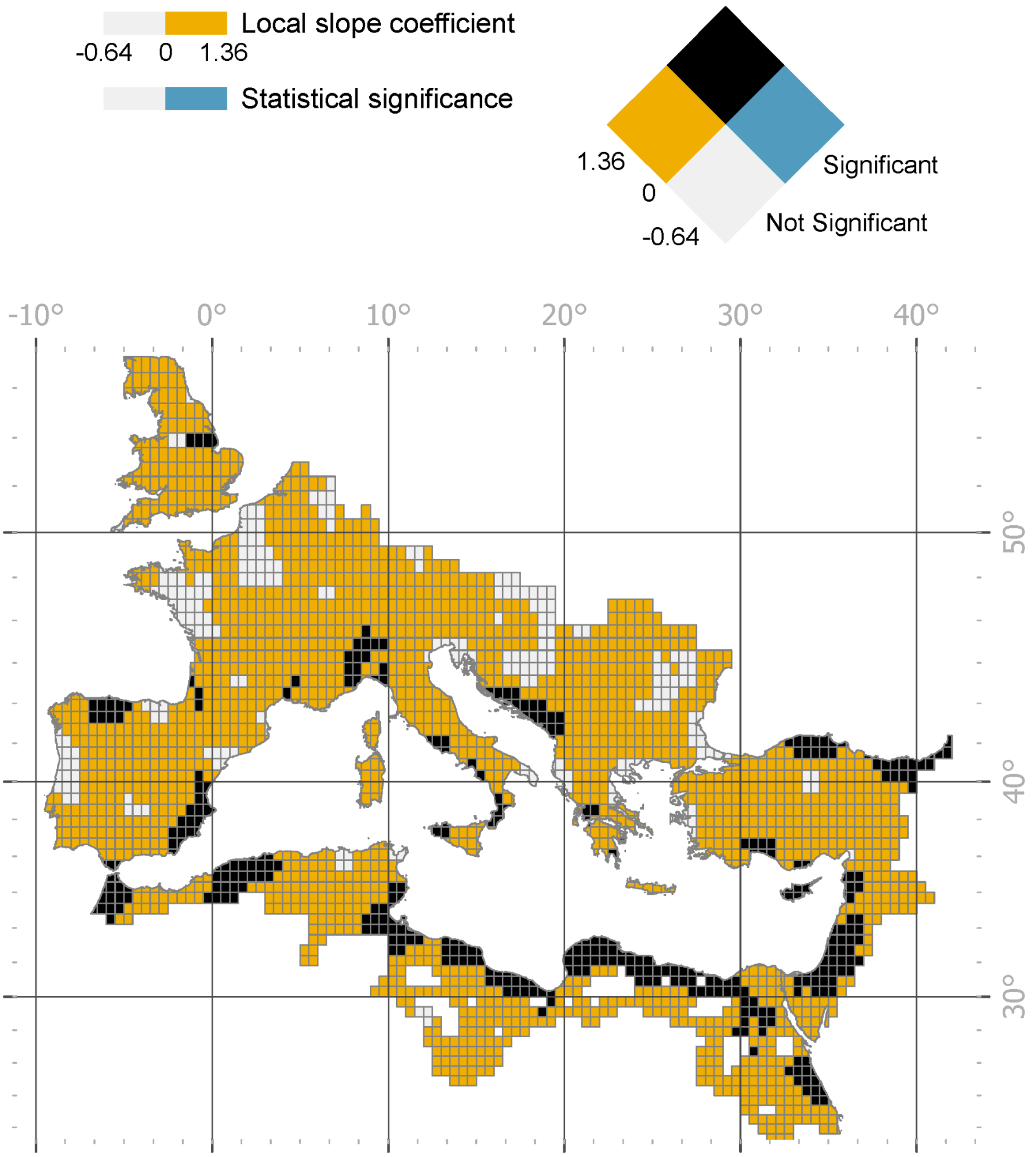

Supplementary Fig. 8. Map of local slope coefficients of the Geographically Weighted Regression analysis for scenario 1. Statistical significance is evaluated against *t*-statistics of the model (>3.68). Black areas have positive local influence of Roman roads on the modern roads and are statistically significant.
