# Supplementary material for: Network science reveals the structure and lasting impact of the Roman road system
Source: Nat Commun. 2026 Sep 15;17:9551. doi: 10.1038/s41467-026-75453-3 (PMC13578412; doi:10.1038/s41467-026-75453-3)
Supplement: Supplementary file 2 [file 41467_2026_75453_MOESM2_ESM.pdf]

<u>Description of Additional Supplementary Files</u>

Supplementary Dataset 1: Mean average and maximum slope, sinuosity and Topographic Position Index (5 km) by road type (Main/Secondary Road) and segment certainty. Values measured for 1,000 m long segments.

Supplementary Dataset 2: Mean average slope, maximum slope, sinuosity and Topographic Position Index (5 km) by road type (Main/Secondary Road) and segment certainty in provinces. Values measured for 1,000 m long segments.

Supplementary Dataset 3: Comparison between road and mountain ranges orientations for provinces where mountains cover more than 33% of province area. Green indicates where the predominant mountain ranges orientation matches the overall provincial road orientation; yellow indicates where the orientation of mountain ranges is perpendicular to roads.

Supplementary Dataset 4: Global network indices for the road network in Roman provinces.

Supplementary Dataset 5: Global network indices for the modern road network in Roman provinces.

Supplementary Dataset 6: Centrality metrics for the nearest node to 871 Roman urban settlements, derived for the Roman road network per province. Scores in the top decile of a province (top 10%) are highlighted in green, while those in the second decile of a province (10- 20%) are highlighted in yellow. The column ‘Capital’ identifies the provincial capital with the value ‘1’.

Supplementary Dataset 7: Empire-wide network centrality values (both raw values and quantile ranking) of the network node closest to that province's capital.
