# Supplementary material for: Network science reveals the structure and lasting impact of the Roman road system
Source: Nat Commun. 2026 Sep 15;17:9551. doi: 10.1038/s41467-026-75453-3 (PMC13578412; doi:10.1038/s41467-026-75453-3)
Supplement: Supplementary file 3 [file 41467_2026_75453_MOESM4_ESM.pdf]

nature portfolio

Corresponding author(s): Tom Brughmans

Last updated by author(s): Jun 1, 2026

Reporting Summary

Nature Portfolio wishes to improve the reproducibility of the work that we publish. This form provides structure for consistency and transparency in reporting. For further information on Nature Portfolio policies, see our Editorial Policies and the Editorial Policy Checklist.

Statistics

For all statistical analyses, confirm that the following items are present in the figure legend, table legend, main text, or Methods section.

n/a	Confirmed	
[ ]	[x]	The exact sample size (*n*) for each experimental group/condition, given as a discrete number and unit of measurement
[x]	[ ]	A statement on whether measurements were taken from distinct samples or whether the same sample was measured repeatedly
[ ]	[x]	The statistical test(s) used AND whether they are one- or two-sided<br>*Only common tests should be described solely by name; describe more complex techniques in the Methods section.*
[ ]	[x]	A description of all covariates tested
[ ]	[x]	A description of any assumptions or corrections, such as tests of normality and adjustment for multiple comparisons
[ ]	[x]	A full description of the statistical parameters including central tendency (e.g. means) or other basic estimates (e.g. regression coefficient) AND variation (e.g. standard deviation) or associated estimates of uncertainty (e.g. confidence intervals)
[ ]	[x]	For null hypothesis testing, the test statistic (e.g. *F*, *t*, *r*) with confidence intervals, effect sizes, degrees of freedom and *P* value noted<br>*Give P values as exact values whenever suitable.*
[x]	[ ]	For Bayesian analysis, information on the choice of priors and Markov chain Monte Carlo settings
[x]	[ ]	For hierarchical and complex designs, identification of the appropriate level for tests and full reporting of outcomes
[ ]	[x]	Estimates of effect sizes (e.g. Cohen's *d*, Pearson's *r*), indicating how they were calculated

Our web collection on statistics for biologists contains articles on many of the points above.

Software and code

Policy information about availability of computer code

Data collection	No original data was collected for this study: it analyses and uses previously published datasets.
Data analysis	The code to reproduce the network analysis results is available at: Matteo Mazzamurro. (2026). MatteoMazzamurro/roman-road-networks: R project for Network Science reveals the structure and lasting impact of the Roman road system - version 1.0.0 (v1.0.0). Zenodo. https://doi.org/10.5281/zenodo.20446953<br>This uses R version 4.3.0 (2023-04-21 ucrt). It relies on the following versions of the packages:<br>tidyverse 2.0.0<br>dplyr 1.1.4<br>sf 1.0.13<br>spatstat 3.0.7<br>raster 3.6.26<br>units 0.8.2<br>igraph 1.4.3<br>sfnetworks 0.6.3<br>spatgraphs 3.4<br>ggraph 2.1.0<br>ggspatial 1.1.9<br>RColorBrewer 1.1.3<br>colorRamps 2.3.1<br>cowplot 1.1.3

deldir 1.0.9
cccd 1.6
Desctool 0.99.58
stplanr 1.2.3

The linear regression analysis was performed in R v.3.2.4 and code is available here: https://github.com/Mcotsar/Ancient_Roads_Exploratory_Analysis

ArcGIS Pro version 3.2 was used to analyse Slope, TPI, orientation, density, GWR were analyzed using the following tools in that version: 'Geographically weighted regression', 'Spatial autocorrelation (Global Moran's I)', 'Linear directional mean', 'Kernel density', Add surface information', Topography toolbox'. Sinuosity was calculated using 'Stream Gradient' and the 'Sinuosity Toolbox' in ArcGIS version 10.1

For manuscripts utilizing custom algorithms or software that are central to the research but not yet described in published literature, software must be made available to editors and reviewers. We strongly encourage code deposition in a community repository (e.g. GitHub). See the Nature Portfolio guidelines for submitting code & software for further information.

Data

Policy information about availability of data

All manuscripts must include a data availability statement. This statement should provide the following information, where applicable:

- Accession codes, unique identifiers, or web links for publicly available datasets
- A description of any restrictions on data availability
- For clinical datasets or third party data, please ensure that the statement adheres to our policy

Roman roads dataset: the version of the Itiner-e dataset used to perform this analysis is available as a polyline vector layer in the shapefile format (itinere_roads.shp), geopackage (itinere_roads.gpkg) and as a geojson database (itinere_roads.geojson) on the Zenodo repository. A growing gazetteer version of the dataset can be viewed and queried on the Itiner-e linked open data platform (https://itiner-e.org). The creation of the dataset is described in de Soto et al.. The version of the road dataset split into 1,000 m long segments with calculated topographic variables, the polygons representing various analytical units (provinces, 0.5° and 0.25°cells) containing topographic and anthropic variables, and additional data generated with analytical tools and used in this article are stored at a Zenodo repository
Modern roads: World Roads dataset available at ArcGIS Online
Provincial boundaries: Roman Empire extent by the year AD 200 available at the Ancient World Mapping Center
Ancient site locations: Pleiades: Gazetteer of Ancient Places
City population estimates: Hanson Cities Database (OXREP)
Modern population data: LandScan Global 2022 population dataset

Research involving human participants, their data, or biological material

Policy information about studies with human participants or human data. See also policy information about sex, gender (identity/presentation), and sexual orientation and race, ethnicity and racism.

Reporting on sex and gender	*Use the terms sex (biological attribute) and gender (shaped by social and cultural circumstances) carefully in order to avoid confusing both terms. Indicate if findings apply to only one sex or gender; describe whether sex and gender were considered in study design; whether sex and/or gender was determined based on self-reporting or assigned and methods used.*<br>*Provide in the source data disaggregated sex and gender data, where this information has been collected, and if consent has been obtained for sharing of individual-level data; provide overall numbers in this Reporting Summary. Please state if this information has not been collected.*<br>*Report sex- and gender-based analyses where performed, justify reasons for lack of sex- and gender-based analysis.*
Reporting on race, ethnicity, or other socially relevant groupings	*Please specify the socially constructed or socially relevant categorization variable(s) used in your manuscript and explain why they were used. Please note that such variables should not be used as proxies for other socially constructed/relevant variables (for example, race or ethnicity should not be used as a proxy for socioeconomic status).*<br>*Provide clear definitions of the relevant terms used, how they were provided (by the participants/respondents, the researchers, or third parties), and the method(s) used to classify people into the different categories (e.g. self-report, census or administrative data, social media data, etc.)*<br>*Please provide details about how you controlled for confounding variables in your analyses.*
Population characteristics	*Describe the covariate-relevant population characteristics of the human research participants (e.g. age, genotypic information, past and current diagnosis and treatment categories). If you filled out the behavioural & social sciences study design questions and have nothing to add here, write "See above."*
Recruitment	*Describe how participants were recruited. Outline any potential self-selection bias or other biases that may be present and how these are likely to impact results.*
Ethics oversight	*Identify the organization(s) that approved the study protocol.*

Note that full information on the approval of the study protocol must also be provided in the manuscript.

Field-specific reporting

Ecological, evolutionary & environmental sciences study design

All studies must disclose on these points even when the disclosure is negative.

Study description	Analysis of the topographic and network structure of roads in the Roman Empire.
Research sample	This study uses an existing dataset: Itiner-e, published as:<br>De Soto, P., Pažout, A., Brughmans, T., Vahlstrup, P.B., Auir, Á., Bongers, T., Christoffersen, J.E.B., Crépy, M., Johansen, M.H., Lewis, J., Manière, L., Massa, M.R., Møller, L.M.H., Redon, B., Renda, G., Şahin, H., Sobotková, A., Spatzek, A.L., Verhagen, P., Weissova, B., 2025. Itiner-e: A high-resolution dataset of roads of the Roman Empire. Sci Data 12, 1731. https://doi.org/10.1038/s41597-025-06140-z<br>The dataset is available on this Zenodo repository: de Soto, P. et al. A High-Resolution Dataset of Roads of the Roman Empire: Itiner-e static version 2024. Zenodo https://doi.org/10.5281/zenodo.17122148 (2025).<br>The dataset includes a digitization of knowledge of the location of roads during the Roman Empire within a research area reflecting the size of the Roman Empire in the late second century CE.
Sampling strategy	No original data was collected for this study.
Data collection	No original data was collected for this study. The data collection of the existing dataset used can be found here: De Soto, P., Pažout, A., Brughmans, T., Vahlstrup, P.B., Auir, Á., Bongers, T., Christoffersen, J.E.B., Crépy, M., Johansen, M.H., Lewis, J., Manière, L., Massa, M.R., Møller, L.M.H., Redon, B., Renda, G., Şahin, H., Sobotková, A., Spatzek, A.L., Verhagen, P., Weissova, B., 2025. Itiner-e: A high-resolution dataset of roads of the Roman Empire. Sci Data 12, 1731. https://doi.org/10.1038/s41597-025-06140-z
Timing and spatial scale	No original data was collected for this study.
Data exclusions	No data was excluded in any analyses.
Reproducibility	All information to reproduce published data analysis results is provided and accessible. All existing data used is openly accessible and the analyses performed are available in code and publication, as well as a specification of the software and tools used. All attempts to reproduce data analysis results were succesful.
Randomization	Randomization not relevant to this study, since no original data was collected for this study.
Blinding	Blinding not relevant to this study, since no original data was collected for this study.

Did the study involve field work? ☐ Yes ☒ No

Reporting for specific materials, systems and methods

We require information from authors about some types of materials, experimental systems and methods used in many studies. Here, indicate whether each material, system or method listed is relevant to your study. If you are not sure if a list item applies to your research, read the appropriate section before selecting a response.

Materials & experimental systems

n/a	Involved in the study
☒	☐ Antibodies
☒	☐ Eukaryotic cell lines
☒	☐ Palaeontology and archaeology
☒	☐ Animals and other organisms
☒	☐ Clinical data
☒	☐ Dual use research of concern
☒	☐ Plants

Methods

n/a	Involved in the study
☒	☐ ChIP-seq
☒	☐ Flow cytometry
☒	☐ MRI-based neuroimaging

Plants

Seed stocks	*Report on the source of all seed stocks or other plant material used. If applicable, state the seed stock centre and catalogue number. If plant specimens were collected from the field, describe the collection location, date and sampling procedures.*
Novel plant genotypes	*Describe the methods by which all novel plant genotypes were produced. This includes those generated by transgenic approaches, gene editing, chemical/radiation-based mutagenesis and hybridization. For transgenic lines, describe the transformation method, the number of independent lines analyzed and the generation upon which experiments were performed. For gene-edited lines, describe the editor used, the endogenous sequence targeted for editing, the targeting guide RNA sequence (if applicable) and how the editor was applied.*
Authentication	*Describe any authentication procedures for each seed stock used or novel genotype generated. Describe any experiments used to assess the effect of a mutation and, where applicable, how potential secondary effects (e.g. second site T-DNA insertions, mosiacism, off-target gene editing) were examined.*
