# Supplementary material for: Network science reveals the structure and lasting impact of the Roman road system
Source: Nat Commun. 2026 Sep 15;17:9551. doi: 10.1038/s41467-026-75453-3 (PMC13578412; doi:10.1038/s41467-026-75453-3)
Supplement: Supplementary file 4 [file 41467_2026_75453_MOESM5_ESM.pdf]

nature portfolio

Peer Review File

Network Science reveals the structure and lasting impact of the Roman road system

Corresponding Author: Dr Tom Brughmans

This file contains all reviewer reports in order by version, followed by all author rebuttals in order by version.

Attachments originally included by the reviewers as part of their assessment can be found at the end of this file.

Version 0:

Reviewer comments:

Reviewer #1

(Remarks to the Author)
See the attached file.

(Remarks on code availability)
The code made available is not replicating the entire analysis summarised in the paper. The weighted regression analysis is produced by a different script.

Moreover, the authors do not made the data available to replicate the analysis. This is a practice that have to be promoted by scientific journals in general and Nature Communications in particular.

A R-project that include all the analysis is advisable. The authors could consider to deliver an R package with appropriate vignettes to instruct how to replicate the paper's content and how to expand the analysis of the original data.

At this stage we cannot guarantee that the script produced is compilable and that the analysis is consistent with the one contained in the paper.

Reviewer #3

(Remarks to the Author)
I co-reviewed this manuscript with one of the reviewers who provided the listed reports. This is part of the Nature Communications initiative to facilitate training in peer review and to provide appropriate recognition for Early Career Researchers who co-review manuscripts.

(Remarks on code availability)

Reviewer #4

(Remarks to the Author)
I co-reviewed this manuscript with one of the reviewers who provided the listed reports. This is part of the Nature Communications initiative to facilitate training in peer review and to provide appropriate recognition for Early Career Researchers who co-review manuscripts.

(Remarks on code availability)

Reviewer #5

(Remarks to the Author)

This paper presents an ambitious and technically sophisticated analysis of the Roman road system at an unprecedented spatial scale. By combining a newly compiled high-resolution dataset with network analysis, the authors address several long-standing questions concerning the structure of Roman roads, their relationship to modern infrastructure, and the role of major cities within the network. The scope of the paper, the analytical approaches, and the dataset used represent a major achievement and make this work well-suited for Nature Communications.

Overall, the paper makes a valuable contribution to the quantitative study of ancient infrastructure. I appreciated particularly the clarity of the research targets and the structure of the final discussion, which makes the paper easy to understand and read.

I recommend this paper for publication after minor revisions.

Below, I outline a few general points that I believe would help strengthen the manuscript. Please also refer to the comments added directly to the manuscript.

1. The methodological contribution of the paper is very strong; however, the historical and conceptual framing would benefit from clearer articulation. While the authors successfully identify and quantify structural patterns in the road network, the implications of these patterns for Roman history are sometimes left implicit or expressed in rather general terms. The argument would be strengthened by situating the study more explicitly within existing debates on Roman mobility, infrastructure, and imperial organisation.

2. The sections examining correlations between Roman and modern roads are among the most interesting in the paper and illustrate pretty well any bias and data limitations. In some cases, however, the interpretations appear to imply a stronger causal continuity than the data can strictly support. The discussion would benefit from greater emphasis on intervening historical processes—such as medieval transport systems, early modern state formation, and differing governance strategies across Roman provinces—that may have shaped long-term infrastructural trajectories.

3. The analysis of the centrality of major cities within provincial road networks is insightful. I wondered whether this could be complemented by applying a different analytical scale, evaluating city centrality within the Roman road network as a whole. Such a comparison between provincial (local) and empire-wide (global) centrality could reveal whether cities that are central at the provincial level retain their importance at an imperial scale, and if so, why. This would be an interesting aspect to explore.

4. I applaud the authors for making both the data and R scripts available online to ensure reproducibility. I note, however, that the GitHub repository containing the code for reproducing the network analysis does not currently include the original dataset. This should be added. In addition, I recommend publishing the dataset via Zenodo using the GitHub release function. Zenodo provides a permanent, citable DOI and offers greater reliability than GitHub for long-term data archiving

(Remarks on code availability)
I applaud the authors for making both the data and R scripts available online to ensure reproducibility. I note, however, that the GitHub repository containing the code for reproducing the network analysis does not currently include the original dataset. This should be added. In addition, I recommend publishing the dataset via Zenodo using the GitHub release function. Zenodo provides a permanent, citable DOI and offers greater reliability than GitHub for long-term data archiving

Reviewer #6

(Remarks to the Author)
The authors attempt and, in my estimation, succeed at producing the first ever truly quantitative study at scale of the Roman road system. The methods and data are significant and the analyses and their interpretations are appropriate in the context of the scale of the question and the limitations of the evidence. The results dispel many anecdotal myths, such as “Roman roads are straight”, “all roads lead to Rome”, “modern roads follow the route of Roman roads”, as well as adding nuance to other forms of received wisdom, including the idea that modern urbanism and industrial development are correlated if not predicated on previous Roman investment in transport infrastructure. These are important corrective results.

For this study to be taken up and extended by domain specialists, especially those from humanities disciplines, much of the statistical jargon and methods of quantitative analysis would need definition in plain language and to be built out in more detail. The scientific format of the paper, separating definitions from results and from interpretations is a further, if unavoidable, impediment. Additionally, since the methods used here are not novel (even if their application on these data is), the number of interpretive areas not undertaken in this, perhaps initial, paper is noteworthy. While it might be too much to ask for an attempt at “what it all means”, there are many next steps that might be briefly included or at least explored if the authors want their paper to be more than an announcement of analyses conducted. For example, the quantitative analysis leads to interpretive regimes that are bounded by quantitative parameters, but many answers in the historical disciplines are also found in qualitative and even subjective evidence. How might these be included? Next, for a subject that is archaeological in nature, the absence of chronology as a variable is striking. What further research would the authors recommend be undertaking for the development of the network over time to be a subject of study? Indeed, this might be a requirement for additional investigation into the question of whether the development of the Roman road system was strategic or organic?

In the end, this is an extremely impressive first analysis of a subject matter that lends itself to such analysis and which, in its absence, has generated many unsubstantiated myths. I recommend its publication, but believe the question if the extension of the results toward the humanistic disciplines in this iteration is best determined by discussion between the authors and editors.

Editorial notes:

The definition of TPI could usefully be moved up to the bottom of page 4 at its first mention from its current position at the bottom of page 5.

Roman for Rome on page 11.

On page 21, the terms "certain", “hypothetical”, and “conjectured” are used in relation to the robustness of the network analysis, but these terms are never defined.

(Remarks on code availability)

Version 1:

Reviewer comments:

Reviewer #1

(Remarks to the Author)
See the attached file

(Remarks on code availability)
I have checked the existence of the code, not the proper functioning of it, or the technical requirements to run it.

Reviewer #3

(Remarks to the Author)
I co-reviewed this manuscript with one of the reviewers who provided the listed reports. This is part of the Nature Communications initiative to facilitate training in peer review and to provide appropriate recognition for Early Career Researchers who co-review manuscripts.

(Remarks on code availability)

Reviewer #4

(Remarks to the Author)
I co-reviewed this manuscript with one of the reviewers who provided the listed reports. This is part of the Nature Communications initiative to facilitate training in peer review and to provide appropriate recognition for Early Career Researchers who co-review manuscripts.

(Remarks on code availability)

Reviewer #5

(Remarks to the Author)
The authors have made all the necessary amendments. I am happy with the revised manuscript. I would recommend it for publication.

(Remarks on code availability)

Reviewer #6

(Remarks to the Author)
I am grateful to the authors for their many revisions and candid replies on suggestions that could not be fully integrated. I have no further comments to offer.

(Remarks on code availability)

Version 2:

Reviewer comments:

Reviewer #1

(Remarks to the Author)
Dear Authors,
After this third round of revisions I am satisfied with the present version of the paper. I think this exchanges where fruitful on both sides. On my side, I learned a lot. I hope you appreciate the constructive aptitude that we as reviewers maintained during in refereeing the subsequent versions of the paper.

Best,

Luca

(Remarks on code availability)

Reviewer #3

(Remarks to the Author)
I co-reviewed this manuscript with one of the reviewers who provided the listed reports. This is part of the Nature Communications initiative to facilitate training in peer review and to provide appropriate recognition for Early Career Researchers who co-review manuscripts.

(Remarks on code availability)

Reviewer #4

(Remarks to the Author)
I co-reviewed this manuscript with one of the reviewers who provided the listed reports. This is part of the Nature Communications initiative to facilitate training in peer review and to provide appropriate recognition for Early Career Researchers who co-review manuscripts.

(Remarks on code availability)

Open Access This Peer Review File is licensed under a Creative Commons Attribution 4.0 International License, which permits use, sharing, adaptation, distribution and reproduction in any medium or format, as long as you give appropriate

credit to the original author(s) and the source, provide a link to the Creative Commons license, and indicate if changes were made.
In cases where reviewers are anonymous, credit should be given to 'Anonymous Referee' and the source.
The images or other third party material in this Peer Review File are included in the article's Creative Commons license, unless indicated otherwise in a credit line to the material. If material is not included in the article's Creative Commons license and your intended use is not permitted by statutory regulation or exceeds the permitted use, you will need to obtain permission directly from the copyright holder.
To view a copy of this license, visit https://creativecommons.org/licenses/by/4.0/

Referee report on *Network Science reveals the structure and lasting impact of the Roman road system* - submitted to *Nature Communications* - December 2025

1 General Assessment

The manuscript presents a quantitative analysis of the road network of the Roman Empire, made possible by the construction of the new *Itiner-e* dataset (de Soto et al., 2025), that many of the authors of the present paper contributed too), which documents nearly 300,000 km of ancient routes. The authors combine data and techniques from archeology, history, network science, and high-resolution spatial analysis to address three historically traditional questions: (i) the degree of straightness of Roman roads, (ii) the centrality of cities and provinces within the imperial road system, and (iii) the persistence of ancient infrastructure within the modern network.

To our knowledge, this is the first paper that exploits *Itiner-e* in a statistical analysis aimed quantifying and testing results on the characteristics of the *Itiner-e* Roman Roads network.

We think that the contribution could be of potential interest to the reader of *Nature Communications.* The main strengths are: i) the use of the most complete and detailed dataset of Roman roads currently available; ii) the effective integration of topographic indicators, geographically weighted regressions (GWR), kernel density estimation, and travel-time-weighted centrality measures; iii) the ability to provide quantitative - not merely qualitative - assessments of themes discussed for decades in archaeological scholarship.

The article is well written, presents some original findings, and is supported by informative visualisations. This work will likely resonate not only in archaeology and ancient history, but also in economic geography, urban studies, and research on long-run spatial persistence.

However, with respect to the existing literature, the manuscript should make clearer what is truly new and where the novelty stems from:

i. Dataset characteristics (use of *Itiner-e* instead of Barrington / DARMC)

ii. Geographical heterogeneities (Empire-wide instead of single-country, or using sub-regional vs country level analysis)

iii. Differences in the methodological approach (different network metrics, topographic controls, alternative statistics indicators)?

We would strongly encourage the authors to add a short subsection in the introduction and in the literature review that clearly spells out: (i) what existing papers have already shown; (ii) what exactly is added by using *Itiner-e* and their specific empirical strategy; (iii) whether their main message is confirmation, refinement, or overturning of previous findings. At the same time, we believe that the paper in its current form falls short of fully exploiting the richness of the *Itiner-e* dataset and of clearly positioning its contribution relative to existing work. We therefore recommend publication only after substantial revision.

That said, **several areas of the manuscript require clarification and methodological strengthening before the manuscript can be accepted for publication in *Nature Communications***. We hope the way we organised our comments and formulated our recommendations will allow to strengthen the merits of the contribution and to minimise its shortcomings. The major and minor points are detailed below.

2 Major Comments

1) *The position of the paper in the literature space.* Although the authors emphasise the novelty and originality of their contribution, this manuscript is not the first exploratory analysis of the Roman road network. Over the past 17 years - starting with Bosker et al. (2008) - numerous works in the social sciences and economics have measured and analysed the role of Roman roads in shaping city location, patterns of urbanisation, and the long-term effects of the Roman transportation system on modern road networks and contemporary economic outcomes (Bosker et al., 2008; Buringh et al., 2012; Bosker et al., 2013; Percoco, 2016; Bosker and Buringh, 2017; Wahl, 2017; Michael and Rauch, 2018; Licio (2021); Dalgaard et al., 2022; Flueckiger et al., 2022; Bottasso et al., 2022; De Benedictis et al., 2023). From this perspective, the research questions addressed in the manuscript are not original. What is original is the use of updated and exceptionally detailed data. Thus, the key contribution lies in the newly released dataset rather than in the novelty of the questions themselves.

For this reason, the authors should engage more thoroughly with previous literature and explicitly compare their approach and findings to prior work, especially to Wahl (2017), Dalgaard et al. (2022), Flueckiger et al. (2022), and De Benedictis et al. (2023), which place Roman roads at the core of their analyses.

A first point concerns the methodological approach. While the aforementioned studies in economic history and applied economics rely on causal

inference and identify the causal impact of Roman transport connectivity on ancient trade and modern economic integration, the manuscript under review instead provides a structural and descriptive account of the Roman road system. It quantifies the network's geometry and topology at extremely high resolution, employing measures such as sinuosity, topographic position index (TPI), centrality, and density, but without attempting any causal identification exercise.

A second difference lies in the quantitative methods used. Previous economic works rely on econometric tools such as instrumental variables, PPML estimations, and credible identification strategies. By contrast, the current manuscript employs topographic analysis, kernel density estimation, and travel-time-weighted centrality, offering a primarily exploratory and descriptive perspective.

The nature of the findings also differs. The economic literature has documented long-term economic persistence - such as the endurance of Roman trade patterns, medieval price correlations, and modern firm-ownership structures. The manuscript under review instead highlights territorial persistence: Correlations between Roman and modern road layouts (without claiming causality), the centrality of capitals, and systematic topographic patterns.

Despite these differences, the manuscript and the existing literature are in our opinion highly complementary. Integrating the high-resolution dataset developed in this manuscript into economic models could substantially improve the precision and reliability of future causal estimates. For this reason, the authors should more clearly position the paper's contribution, articulate how their dataset opens new "roads" for economic analysis, and emphasise the descriptive - rather than causal - but foundational nature of their work.

2) *Heterogeneity.* The dataset exhibits substantial heterogeneity: 2.7% of segments are classified as "certain," while nearly 90% are "conjectural." The authors address uncertainty only for the centrality measures (by randomly removing 20% of uncertain segments), whereas its impact on other results - road density, linear regressions, topographic indicators - is handled more qualitatively. To provide more representative statistics it would be interesting to incorporate the information about certainty into the regression models (for example, as weights or additional control variable). Replicate, at least in the appendix, selected analyses using only "certain" + "hypothetical" segments would also be helpful. Moreover, the manuscript would benefit from a more detailed discussion of the regions that may suffer from reconstruction biases potentially distorting the distributions of sinuosity and TPI (e.g., North Africa or the interior of the Iberian Peninsula). These additions would strengthen the empirical credibility of the study by demonstrating robustness

to areas with greater uncertainty.

3) *Finer spatial grids.* The 0.5° grid - approximately 55 km at Mediterranean latitudes - is suitable for an empire-wide analysis but remains quite coarse for capturing local relationships among roads, settlements, and geomorphology. The alternative of using provinces does not resolve this limitation, as provincial boundaries are even coarser and not designed for fine-grained spatial analysis. Employing finer grid cells (for example, 0.25°) would provide greater spatial detail, bring the study closer to the conventions in the existing literature, which generally relies on smaller cell sizes, and facilitate comparison with previous work conducted at higher spatial resolution. Finally, the chosen GWR bandwidth (100 km) is tied to the 0.5° grid structure (see a related suggestion below); however, it appears large and may smooth out meaningful local variation. This raises an important methodological question: What would the results look like if the GWR were estimated on smaller spatial units? It would be interesting to provide some descriptive statistics based on finer spatial grids.

4) *Causality (again).* The authors argue that there is "continuity" and that Roman roads are "good predictors" of modern ones. It would be advisable to avoid terms such as "predictor" or claims that Roman roads "explain" modern road patterns. This type of causal interpretation has been established in previous work using detailed econometric analysis, whereas the approach adopted here relies primarily on Pearson correlation coefficients and GWR to describe the relationship between ancient and modern infrastructure.

In this manuscript, the correlation is moderate (Pearson R $\approx$ 0.46-0.54), and the GWR maps (Fig. 3a-c) display substantial spatial heterogeneity, with positive associations in some regions and null or even negative associations in others. These patterns may reflect a combination of geomorphological constraints, historical or institutional persistence, or biases stemming from more intensive archaeological research near modern settlements. A more cautious formulation could distinguish clearly among three mechanisms: i) topographic persistence, whereby both ancient and modern roads cluster in similar natural transit corridors; ii) historical or institutional persistence, which might shape long-run infrastructural trajectories; iii) detection and research bias, arising from uneven archaeological documentation. Clarifying these dimensions could help avoid over-interpretation and provide a more accurate account of what the evidence can support.

5) *Centrality.* The authors compute centrality by comparing Roman roads with modern road networks. However, the manuscript does not clearly de-

fine what is meant by "main modern roads." It remains unclear whether the analysis includes only motorways or also primary roads. This point should be specified explicitly. Moreover, the authors should justify the restriction to major modern roads when computing centrality. Including a broader set of road types could provide a more representative picture of contemporary accessibility, which may not be fully captured by focusing exclusively on high-capacity roads.

Since the manuscript does not include riverine and maritime connections, We would suggest better clarifying in the conclusions that terrestrial centrality does not necessarily correspond to actual multimodal centrality. The authors should indicate how the network structure and centrality rankings might change with the inclusion of riverine and maritime layers (both ancient and contemporary) and, even if only qualitatively, discuss the extent to which the current findings are driven by the choice of a purely terrestrial network.

6) *Start and end points.* The authors state that "Sinuosity can be used as a measure of the "straightness" of the road, calculated as the ratio of actual road length to the straight-line distance between the start and end point (Fig. 1c)." However, the definition of the start and end points is ambiguous. It would be helpful to clarify whether these points correspond to the limits of the entire road or to the endpoints of the 1 km road segments used in the analysis, particularly given the earlier statement that "Results were derived for 1 km stretches of roads to enable comparability."

Another point has to do with the comparison between pre-Roman and Roman roads. The authors compare Roman with modern roads finding that "[. . .] major modern roads (Fig. 1d) tend to be straighter than ancient roads [. . .]". While this result is interesting, given that the Romans were known for constructing straight their roads, it would be interesting also comparing Roman with pre-Roman roads.

7) *Biases and uneven mapping of Roman Roads.* An important issue concerns the spatial bias in the mapping of Roman roads. The *Itiner-e* data paper itself is very explicit that coverage, spatial resolution and source reliability vary substantially across regions due a variety of factors, and it provides representativity, reliability and confidence maps precisely to visualise these heterogeneities.

Given this, it would be useful if the authors engaged more directly with the question of which regions are better or worse represented in their analyses, and why. For example, the confidence map highlights large areas of low representativity and/or low reliability in parts of northern Italy (Tuscany,

Marche, Po Valley), the Western Balkans, the Upper and Middle Danube, Corsica, Sardinia and Central Anatolia. These are precisely regions where the correlation between Roman and modern roads appears weaker or more heterogeneous in their geography weighted coefficients maps. It would help the analysis if the authors would:

i. Explicitly overlay their main results (correlations and geographically weighted coefficients maps) with the confidence categories from *Itiner-e* (de Soto et al., 2025);

ii. Discuss to what extent low correlations in some regions might reflect genuine heterogeneity in the coverage of Roman Roads versus data gaps or low-resolution digitisation.

The authors should consider using the confidence classes in the maps of the de Soto et al., (2025) paper at least to provide robustness checks that restrict the analysis to polygons with medium/high reliability and average/above average representativity.

This would both acknowledge the non-uniform quality of the underlying archaeological record and strengthen the credibility of regional comparisons.

8) *Interpreting regional variation in Roman-modern correlations with the new dataset.* The manuscript rightly emphasises that the new dataset reveals a more complex picture of the relationship between Roman and modern roads, including regions of Europe with low correlation and parts of the Near East and North Africa where correlation is surprisingly high, contrary to some earlier expectations. The authors conclude that "other variables, likely cultural, institutional, economic and political, are on a regional scale stronger predictor of post-Roman infrastructural and economic developments".

This observation opens an important question that could be developed further: To what extent are these new patterns driven by the improved road data in *Itiner-e*, and to what extent by differences in the modern road data or in the methodology used in this analysis vs the ones in previous papers? Since *Itiner-e* nearly doubles the known road length relative to DARMC and greatly increases spatial precision in several regions (Iberian Peninsula, Aegean, Egypt, etc.), (de Soto et al., 2025) it would be valuable to clarify whether:

i. The regions where they now find higher correlations (e.g. parts of the Near East and North Africa) are precisely those where *Itiner-e* adds many roads or enhances spatial accuracy.

ii. Conversely, some European regions with low correlation (e.g. Brittany, parts of southern Italy) coincide with areas of low representativity / low reliability in the confidence map.

A simple way to address this would be to show, for some representative

regions, how the Roman-modern correlation changes when using DARMC vs *Itiner-e*, or when restricting the *Itiner-e* data to high-confidence polygons. This would help the reader to understand whether the new dataset truly changes our substantive understanding of where Roman roads matter most for modern infrastructure, or whether the differences mainly reflect the underlying archaeological/documentary biases or statistical choices.

If the authors wish to argue that "other variables" are locally stronger predictors of modern infrastructure, it would be useful to at least sketch what these could be (e.g. medieval and early-modern state capacity, post-Roman trade routes, religious or political frontiers) and, if possible, include a simple multivariate specification where Roman roads are analysed jointly with a set of such covariates.

9) *Chronology and the military vs non-military purposes of roads.* The authors point out that not all Roman roads were built with purely military objectives. The point on the military needs determining the development of the network has been never questioned by historians, at least for the Consular (main) roads. One of the strengths of the *Itiner-e* dataset is that, where available, it includes fields on chronology and on the magistrate or emperor under whose tenure the road was formalised (Lower-Date, Upper-Date, Construction Period, see table 2 of De Soto et al.). Although the data paper emphasises that chronological information is incomplete, these attributes still offer an opportunity for at least exploratory differentiation between major phases of road construction. Therefore, the dataset appears well suited to testing whether there are systematic differences between roads created in the Republican period (509-27 BCE) and those formalised in the Imperial period (27 BCE-ca. 400 CE). By using the information on Roman sites could ask whether:

i. Republican roads are more strongly associated with military expansion and frontier consolidation.

ii. Whereas later Imperial roads serve more clearly administrative, commercial or accessibility functions within already consolidated provinces.

Even if a full empire-wide chronological reconstruction is beyond the scope of the current paper, a limited analysis on the subset of segments with known construction periods, or at least a descriptive discussion of how much of the network can be reliably dated and what spatial patterns emerge, would enrich the argument that Roman roads cannot be reduced to a purely military infrastructure. It would also make the most of the chronological fields that have been included in *Itiner-e*. 10) Cultural features, Pleiades, and the distinction between main and secondary roads. The manuscript briefly refers to "cultural variables" and acknowledges that major roads were often linked

to military campaigns, but the potential of the data to explore cultural and functional differentiation within the network is not fully exploited.

Itiner-e is explicitly linked to Pleiades and other gazetteers of ancient places, which contain rich information on the location of forts, sanctuaries, temples, theatres, baths and thermal complexes, cemeteries, and other features of Roman daily and religious life. Some regions - around major sanctuaries, spa towns, or legionary bases - may show distinctive patterns in how roads branch, converge, or change hierarchy. *Itiner-e* itself distinguishes between “Main Roads” and “Secondary Roads”, based on milestones, inclusion in itineraries and continuity with historically known routes (de Soto et all, 2025). A more explicit discussion of this potential - and of how secondary roads might reflect cultural and everyday mobility rather than only military or administrative needs - would help underline the added value of the new dataset.

Using *Itiner-e* plus Pleiades for some regions, would illustrate how cultural factors shaped differently the layout of both major and minor roads. Two lines of analysis could be hinted at more clearly:

i. Functional differences between main and secondary roads.

ii. If it is true that secondary roads are likely to have been less directly tied to long-distance military campaigns and more to local economic, social and cultural connectivity, correlating the density of secondary roads with the presence of thermal sites, sanctuaries, villas, forts or other specific categories of sites could help to substantiate the claim that Roman roads also served non-military purposes and to show which parts of the network were more closely associated with which types of activities.

3 Minor Comments

1) The structure of the paper is difficult to follow and can be better organised. Placing the Materials and Methods section at the end of the manuscript makes it harder for the reader to fully understand the analysis while reading the main text. Key methodological information is often required to interpret the results, yet the reader is forced to repeatedly move back and forth within the paper to retrieve these details. We recommend integrating methodological information into the sections where it is most directly relevant or alternatively repositioning the Materials and Methods section earlier in the manuscript. This would substantially improve readability and allow the reader to better assess the assumptions, data, and techniques at the point where they are discussed.

2) The paper contains many interesting ideas and pieces of information scattered throughout the manuscript, but several of them are only briefly mentioned and not sufficiently developed. As a result, the reader is left wanting more explanation and context. While we recognise that these aspects cannot be explored in great detail without substantially increasing the length of the paper, providing some additional discussion would help address otherwise unresolved curiosity. In particular, the following points would benefit from further elaboration:

i. Pre-existing roads used and re-paved by the Romans.

ii. Alpine roads show lower slope values, orientation of roads.

iii. Modern roads are slightly more negatively correlated with elevation and TPI than Roman roads are.

iv. While the Roman roads are globally good predictors of modern road infrastructure there are other variables, likely cultural, institutional, economic and political, that are on a regional scale stronger predictor of post-Roman infrastructural and economic developments.

v. Cultural features like the Roman frontier (Rhine, and Danube to some extent): Are these cultural or geographical features?

3) The authors acknowledge that the network aggregates multiple historical phases. It would be useful to discuss, even qualitatively, which of the results may be sensitive to the lack of temporal differentiation in the network. Clarifying how the aggregation of different phases might blur regional or temporal variation would help the reader better assess the robustness and interpretation of the findings.

4) Regarding the "cultural variables" discussed on page 6, we would not classify ancient sites and modern population as cultural variables. Rather, these measures are more appropriately interpreted as indicators of urbanisation. Reframing them as such would improve conceptual clarity.

5) An opening parenthesis is never closed in the following passage: "(mainly in Western Europe, the Pannonian basin, North Africa and low lying regions of Italy and Asia Minor (Po valley, Puglia, central Anatolia and Syria)".

6) In Figure 1, panel f) is described as a "linear regression of the topographic variables". This wording is misleading. The figure appears instead to present a matrix composed of histograms, Pearson correlation coefficients, and scatterplots. The caption and related text should be revised accordingly to accurately reflect the content of the figure.

7) The manuscript sets the minimum neighbourhood distance for the GWR kernel at 1000 km because the global Moran's I z-score peaks at the 900 km band. Because z-scores inflate with the large number of cell pairs at long distances, that peak does not by itself indicate the scale at which the substantive autocorrelation is strongest. Standard practice is to select a GWR bandwidth by optimising an information criterion (AICc or cross-validation) and/or by ensuring a minimum neighbour count for numerical stability. Please clarify:

i. whether an adaptive bandwidth subsequently underwent AICc/CV optimisation in ArcGIS, and

ii. what practical consideration (e.g., required minimum number of neighbours per local regression) led to the 1000 km threshold.

Stating these points explicitly would make the bandwidth choice transparent and reassure readers that the local models are neither under- nor over-smoothed.

8) The factor 0.6 in the stated pace equation diverges from the canonical "inverse-Tobler" value of $1/6 \approx 0.167$. On level ground it implies a speed of only about 1.4 km h^{-1}, far below the walking or cart speeds normally assumed in Roman-period studies. Because the edge weights in the betweenness analysis depend exponentially on slope, the choice of the factor affects which routes appear shortest. Please either

i. confirm that the 0.6 is a typographical error and provide the corrected value, or

ii. supply a citation and brief explanation (e.g., empirical adjustment for cart traffic) that justifies using a slower, non-standard variant of Tobler's function.

9) The stated study-area of 5.27 billion km^2 is impossible (it exceeds Earth's surface area) and conflicts with the 5.5 million km^2 value cited elsewhere in the paper. Using 5.27 million km^2 reproduces the published density of 56.748 m km^2, showing that the text contains a typographical order-of-magnitude error. Please correct the area value.

10) The six orientation classes are said to be "covering of the compass each," yet the labels given (N-S, NNE-SSW, NE-SW, E-W, SE-NW, SSE-NNW) do not correspond to the centres of six contiguous 30° bins. As written, a reader cannot deduce the exact angular boundaries used to generate Figure 1b. Please specify the numerical limits (or mid-points) of each class so that the orientation analysis can be replicated unambiguously.

11) The Discussion treats the lower sinuosity of main roads as noteworthy

evidence for "straight Roman roads," whereas earlier sections describe the main-versus-secondary difference as small ("negligible" / "does not change much"). Clarifying the magnitude of the effect in the Discussion - perhaps by noting that it is minor but still the closest Roman analogue to modern straightness - would make the narrative more consistent.

12) We initially found the phrase "density value of 1 where the surface overlaps with the highest values of the segment" ambiguous, since ArcGIS's Kernel Density tool normalises a quartic kernel to a peak of 1 only as an intermediate step before rescaling outputs to line-length-per-area units after all kernels are summed. Clarifying that the final raster values are the standard ArcGIS line-density outputs and that no custom weighting or rescaling was introduced would remove this uncertainty.

13) The statement that the observed local correlations are "rather accidental" is currently tied to the low global adjusted R^2 (0.10). A low global R^2 in a GWR does not, by itself, render all local relationships spurious. It would be clearer to attribute the likely spuriousness to the pattern of correlations being confined to large modern cities - where archaeological recovery of Roman roads is better - rather than to the summary fit statistic alone.

14) Statement ["previous studies suggested lower correlation"] initially led us to pause, because Dalgaard et al. (2022) describe "weaker persistence" chiefly through a smaller coefficient, whereas the present paper uses local GWR R^2 as its correlation measure. After revisiting Dalgaard, however, we see that their camel-belt specification also reports a noticeably lower goodness-of-fit, so summarising their finding as "lower correlation" is therefore defensible. To eliminate any ambiguity for readers who recall only the coefficient result, you might add a brief clause clarifying that both studies refer to explanatory power (model fit), not merely slope magnitude.

15) Table S3 lists an adjusted R^2 of 0.3482 (unadjusted 0.3662) for Scenario 3, whereas the main text cites 0.39. Please verify which figure is correct and amend either the text or the table so the reported statistics are consistent.

16) The phrase (Fig. 3a; SI Appendix, Table S3) "a strong relationship but not too significant" conflates two different ideas. R^2 indicates goodness-of-fit, while statistical significance would be assessed with an F-test p-value (which, given 2,500+ observations, would be vanishingly small for an R^2 of 0.47). Consider rephrasing to convey that the model explains a substantial yet far-from-total share of the variance, without using the term "significant."

17) (p.5 paragraph "The straightness of Roman roads . . . ") In the sentence "We observe that sinuosity is slightly positively correlated with maximum slope (Fig. 1f), i.e. growing average slope correlates with higher sinuosity of the roads." "i.e." implies that the correlation with maximum slope is the same statement as the correlation with average slope, even though the variables are distinct. Replacing "i.e." with a connector such as "and similarly" (or separating the findings) would report the two positive correlations without equating them.

——————————————-

4 References

Bosker, M., Brakman, S., Garretsen, H., De Jong, H., & Schramm, M. (2008). Ports, plagues and politics: Explaining Italian city growth 1300-1861. *European Review of Economic History*, 12(1), 97-131.

Bosker, M., & Buringh, E. (2017). City seeds: Geography and the origins of the European city system. *Journal of Urban Economics*, 98, 139-157.

Bosker, M., Buringh, E., & van Zanden, J. L. (2013). From Baghdad to London: Unraveling urban development in Europe, the Middle East, and North Africa, 800-1800. *The Review of Economics and Statistics*, 95(4), 1418-1437.

Bottasso, A., Conti, M., Robbiano, S., & Santagata, M. (2022). Roads to innovation: Evidence from Italy. *Journal of Regional Science*, 62, 981-1005.

Buringh, E., van Zanden, J. L., & Bosker, M. (2012). Soldiers and booze: The rise and decline of a Roman market economy in North-Western Europe. Utrecht University, *CGEH Working Paper* No. 32.

Dalgaard, C. J., Kaarsen, N., Olsson, O., & Selaya, P. (2022). Roman roads to prosperity: Persistence and non-persistence of public goods provision. *Journal of Comparative Economics*, 50(4), 896-916.

De Benedictis, L., Licio, V., & Pinna, A. M. (2023). From the historical Roman road network to modern infrastructure in Italy. *Journal of Regional Science*, 63, 1162-1191.

De Soto, P., et al. "Itiner-e: A high-resolution dataset of roads of the Roman Empire." *Scientific data*, 12.1 (2025): 1731.

Flueckiger, M., Hornung, E., Larch, M., Ludwig, M., & Mees, A. (2022). Roman transport network connectivity and economic integration. *The Review of Economic Studies*, 89, 774-810.

Licio, V. (2021). "When history leaves a mark: A new measure of Roman roads." *Italian Economic Journal*, 7.1 (2021): 1-35.

Michaels, G., & Rauch, F. (2018). Resetting the urban network: 117-2012. *The Economic Journal*, 128(608), 378-412.

Percoco, M. (2016). Highways, local economic structure and urban development. *Journal of Economic Geography*, 16(5), 1035-1054.

Wahl, F. (2017). Does European development have Roman roots? Evidence from the German limes. *Journal of Economic Growth*, 22(3), 313-349.

Referee report (second round) for the manuscript "Network Science reveals the structure and lasting impact of the Roman road system," submitted to Nature Communications

General Assessment

We thank the authors for submitting a revised version of the manuscript that we evaluated individually and discussed collectively. The paper has improved in terms of clarity, positioning within the literature, and methodological transparency compared to the initial submission. We think that the authors-reviewers interaction was positive and productive as far as the paper's content.

The contribution remains primarily descriptive and exploratory, but this is now more clearly acknowledged and explicitly framed as complementary to causal econometric studies. This clarification significantly improves the conceptual coherence of the manuscript and helps align the empirical strategy with the scope of the conclusions. We think that the paper will become a benchmark in this field of study and the minor revisions that we ask the authors to implement are mainly due to the conviction of the important role the paper will play as a reference for future works.

The treatment of uncertainty is now more transparent and methodologically sound. In addition, the discussion of data heterogeneity—both in terms of representativeness and reliability—has been expanded, and the new robustness exercises using smaller spatial grid cells (0.25 degrees) are a useful addition. The reconsideration of the GWR bandwidth and neighborhood specification, now based on a standard AICc optimization criterion with an adaptive neighborhood structure further strengthens the analysis.

The authors have also addressed several technical concerns raised in our previous review, including the order-of-magnitude error in the reported study area, the clarification of sinuosity endpoints, and a more precise description of the modern road dataset.

Overall, the revision represents a substantial improvement over the original version. That said, we think that a small number of methodologically relevant issues remain and should be addressed before the manuscript can be considered for acceptance. At this stage, we recommend minor revisions focused on these remaining points.

Comments

1) Positioning in the literature. While the narrative is now more coherent and the complementary nature of the contribution is clearly articulated, one aspect could still be sharpened. In particular, the paper would benefit from a more explicit distinction between i) findings that confirm previous results, ii) those that refine them, and iii) those that lead to substantively different conclusions.

At present, this differentiation is still largely implicit. For example, on page 18 the authors note that "*This result puts into question the previous finding by Dalgaard et al., where high correlation between Roman and modern roads was postulated for Western Europe and low correlation for North Africa and the Near East […]*". However, the broader implications of this statement for the existing literature are not fully systematized.

Given the relevance that this paper will play for future research in this field, a brief and explicit summary paragraph (even with the use of a Table of results) would help the readers substantially. This would strengthen the paper's positioning and make its added value more immediately visible.

2) Uncertainty. The paper still treats uncertainty largely as a robustness dimension rather than integrating it directly into estimation. We think this could be acceptable for an exploratory, high-level Nature Communications paper, but the authors should be careful not to overstate "robustness" where representativity is structurally low. A sharp sentence

in the Results or Discussion paragraph clarifying that these are robustness checks, not corrections for measurement error, would prevent overinterpretation.

3) Causality language. The GWR section still uses language such as “influence”, “effect”, “prediction”, “explanatory variable” (for instance, on page 27 the authors states “[…] *the influence of Roman roads on the location of modern roads varies widely*”; “*There is no contiguous region or area where the model shows good prediction*.”). Even if intended descriptively, this phrasing can be read as causal by non-technical readers. We recommend systematically replacing:
“influence/effect” → “association/relationship”; “predict/prediction” → “fit/local association/ explanatory power”.

4) Travel-time weighting (Tobler hiking function constant). The manuscript continues to report a factor of 0.6 in the stated pace equation without further explanation or correction. This parameter choice requires clarification. If it is a typo error, the constant should be corrected and the results updated accordingly. If it is intentional, the authors should provide a clear justification and citation for this specification. For example, whether it reflects calibration to carts or pack animals, terrain constraints, or empirical estimates of Roman travel times.
In addition, a sensitivity analysis would be appropriate. The authors should demonstrate that the main centrality results (particularly the distinction between primary and secondary roads and the inferred centrality of cities) remain stable under plausible variations of this parameter. Given that the betweenness-based findings are central to the paper’s contribution, this issue is not merely technical but substantively important for the manuscript’s credibility and acceptance.

5) Geographical heterogeneity. The paper still presents the results largely as a broad overview of the legacy of Roman roads across a wide geographical context. As noted in point 1, the authors should more clearly distinguish between: (i) genuinely novel results that arise at the geographical level from the use of a more complete dataset; (ii) new evidence that revisits or extends previous findings, such as the discussion related to the evidence in Dalgaard et al. for North Africa; and (iii) results that mainly confirm the existing literature on the persistence of historical infrastructure and its effects on modern outcomes in some geographical areas. Structuring the results section around these three elements—potentially as separate subsections—would significantly improve the clarity of the contribution and help readers understand what is new relative to the existing literature from a geographical standpoint.

Minor remark
6) In sentence *“[…] which is complementary to previous work in economic history that focused on identifying causal inference*” on page 3 we would rephrase as “*which is complementary to previous work in economic history and historical economics that focused on identifying causal inference*”.

Reply to reviews

We are grateful for the deep level of engagement with our work by the reviewers, resulting in these highly constructive and detailed comments. We have made major revisions in light of these, including new robustness checks, figures and a much elaborated interpretation and contextualisation in the literature. We present here a reply in bold to each individual comment. All changes are highlighted in the manuscript in light green, and include new figures, as well as new supplementary tables, figures and files. We believe the paper is stronger thanks to this.

REVIEWER COMMENTS

Reviewer #1 (Remarks to the Author):

Comments from the file created by Reviewer #1.

"General comment:The article is well written, presents some original ndings, and is supported by informative visualisations. This work will likely resonate not only in archaeology and ancient history, but also in economic geography, urban studies, and research on long-run spatial persistence. However, with respect to the existing literature, the manuscript should make clearer what is truly new and where the novelty stems from:

i. Dataset characteristics (use of Itiner-e instead of Barrington / DARMC)

ii. Geographical heterogeneities (Empire-wide instead of single-country, or using sub-regional vs country level analysis)

iii. Differences in the methodological approach (different network metrics, topographic controls, alternative statistics indicators)?"

It is so great for us to receive a review that engages with our work in such detail and makes elaborate constructive comments based on this engagement. Thank you! We have made major modifications accommodating all major and minor comments (including contextualising our novelty with respect to existing literature).

Major comment 1) The position of the paper in the literature space. Although the authors emphasise the novelty and originality of their contribution, this manuscript is not the first exploratory analysis of the Roman road network. Over the past 17 years - starting with Bosker et al. (2008) - numerous works in the social sciences and economics have measured and analysed the role of Roman roads in shaping city location, patterns of urbanisation, and the long-term effects of the Roman transportation system on modern road networks and contemporary economic outcomes (Bosker et al., 2008; Buringh et al., 2012; Bosker et al., 2013; Percoco, 2016; Bosker and Buringh, 2017; Wahl, 2017; Michael and Rauch, 2018; Licio (2021); Dalgaard et al., 2022; Flueckiger et al., 2022; Bottasso et al., 2022; De Benedictis et al., 2023). From this perspective, the research questions addressed in the manuscript are not original. What is original is the use of updated and exceptionally detailed data. Thus, the key contribution lies in the newly released dataset rather than in the novelty

of the questions themselves. For this reason, the authors should engage more thoroughly with previous literature and explicitly compare their approach and findings to prior work, especially to Wahl (2017), Dalgaard et al. (2022), Flueckiger et al. (2022), and De Benedictis et al. (2023), which place Roman roads at the core of their analyses. A first point concerns the methodological approach. While the afore-mentioned studies in economic history and applied economics rely on causal inference and identify the causal impact of Roman transport connectivity on ancient trade and modern economic integration, the manuscript under review instead provides a structural and descriptive account of the Roman road system. It quantifies the network's geometry and topology at extremely high resolution, employing measures such as sinuosity, topographic position index (TPI), centrality, and density, but without attempting any causal identification exercise.

A second difference lies in the quantitative methods used. Previous economic works rely on econometric tools such as instrumental variables, PPML estimations, and credible identification strategies. By contrast, the current manuscript employs topographic analysis, kernel density estimation, and travel-time-weighted centrality, offering a primarily exploratory and descriptive perspective.

The nature of the findings also differs. The economic literature has documented long-term economic persistence - such as the endurance of Roman trade patterns, medieval price correlations, and modern firm-ownership structures. The manuscript under review instead highlights territorial persistence: Correlations between Roman and modern road layouts (without claiming causality), the centrality of capitals, and systematic topographic patterns.

Despite these differences, the manuscript and the existing literature are in our opinion highly complementary. Integrating the high-resolution dataset developed in this manuscript into economic models could substantially improve the precision and reliability of future causal estimates. For this reason, the authors should more clearly position the paper's contribution, articulate how their dataset opens new \roads"" for economic analysis, and emphasise the descriptive - rather than causal - but foundational nature of their work."

We have made strategic additions to more clearly position the contribution of our work with respect to previous work. The introduction clearly states the points mentioned by the reviewer in their general comment (i) the use of the new more detailed dataset, (ii) empire-wide focus as well as all provinces. We have modified the introduction by adding additional references and specifying (iii) differences in methodological approach. We agree with the reviewer that this makes our work complementary to previous economics work, and have stated this even more explicitly now. We have added a section to the discussion elaborately positioning our contribution and the complementarity of our work to previous work. We emphasise the exploratory, spatially-detailed, and empire-wide aspects as novelties, and we have modified the discussion to clarify how these findings are complementary to previous work (some of which focuses on causality, uses different road data, uses econometrics methods, and can be regionally specific). We have cited the references which were not yet in the paper in appropriate places in the introduction and discussion, and throughout the text.

Major comment 2) Heterogeneity. The dataset exhibits substantial heterogeneity: 2.7% of segments are classified as "certain," while nearly 90% are "conjectural." The authors

address uncertainty only for the centrality measures (by randomly removing 20% of uncertain segments), whereas its impact on other results - road density, linear regressions, topographic indicators - is handled more qualitatively. To provide more representative statistics it would be interesting to incorporate the information about certainty into the regression models (for example, as weights or additional control variable). Replicate, at least in the appendix, selected analyses using only "certain" + "hypothetical" segments would also be helpful. Moreover, the manuscript would benefit from more detailed discussion of the regions that may suffer from reconstruction biases potentially distorting the distributions of sinuosity and TPI (e.g., North Africa or the interior of the Iberian Peninsula). These additions would strengthen the empirical credibility of the study by demonstrating robustness to areas with greater uncertainty.

The impact of road certainty on results is assessed for both descriptive topographical and network analyses. The results per certainty categories for topographic analysis (slope, TPI, sinuosity) are reported in Supplementary Information (previously SI Appendix) (Supplementary Fig. 1, Supplementary Dataset 1). For the GWR, we believe the comparison with data reliability and representativity was more appropriate (which we implemented in detail, see reply to major comment 7), because both GWR and the confidence map concern analyses grids rather than being road segment level metrics like slope, TPI , Sinuosity, and road segment certainty.

Major comment 3) Finer spatial grids. The 0.5° grid - approximately 55 km at Mediterranean latitudes - is suitable for an empire-wide analysis but remains quite coarse for capturing local relationships among roads, settlements, and geomorphology. The alternative of using provinces does not resolve this limitation, as provincial boundaries are even coarser and not designed for fine-grained spatial analysis. Employing finer grid cells (for example, 0.25°) would provide greater spatial detail, bring the study closer to the conventions in the existing literature, which generally relies on smaller cell sizes, and facilitate comparison with previous work conducted at higher spatial resolution. Finally, the chosen GWR bandwidth (100 km) is tied to the 0.5° grid structure (see a related suggestion below); however, it appears large and may smooth out meaningful local variation. This raises an important methodological question: What would the results look like if the GWR were estimated on smaller spatial units? It would be interesting to provide some descriptive statistics based on finer spatial grids.

We thank the reviewer for this suggestion, and the GWR analysis for a finer spatial grid of 0.25°x0.25° cells was undertaken, and a new Supplementary Figure 5 and Supplementary Table 6 has been added. Accompanying figures and tables were added to the Supplementary Information. We provide a more detailed reply to the GWR settings in our reply to Minor comment 7 which raises a related comment, and has led to new analyses and revisions. Both comments led to an elaborate new Method section 'Robustness of spatial regression' which is also summarised in a new paragraph in the body text.

Major comment 4) Causality (again). The authors argue that there is ""continuity"" and that Roman roads are ""good predictors"" of modern ones. It would be advisable to avoid terms such as \predictor"" or claims that Roman roads ""explain"" modern road patterns. This type of causal interpretation has been established in previous work using detailed econometric analysis, whereas the approach adopted here relies primarily on Pearson correlation

coefficients and GWR to describe the relationship between ancient and modern infrastructure.

In this manuscript, the correlation is moderate (Pearson R _ 0.46-0.54), and the GWR maps (Fig. 3a-c) display substantial spatial heterogeneity, with positive associations in some regions and null or even negative associations in others. These patterns may reflect a combination of geomorphological constraints, historical or institutional persistence, or biases stemming from more intensive archaeological research near modern settlements. A more cautious formulation could distinguish clearly among three mechanisms: i) topographic persistence, whereby both ancient and modern roads cluster in similar natural transit corridors; ii) historical or institutional persistence, which might shape long-run infrastructural trajectories; iii) detection and research bias, arising from uneven archaeological documentation. Clarifying these dimensions could help avoid over-interpretation and provide a more accurate account of what the evidence can support.

We agree with the need to emphasize the exploratory nature of our results, and the word "predictor" in the article was replaced and the affected sentences rephrased around terms such as "relationship" and "correlation". The text in the Results and Discussion sections was expanded with further discussion of the mechanisms affecting our results, especially with regard to the GWR, along the three lines suggested by the reviewer. We have further nuanced our interpretation of the spatially heterogeneous results as suggested by the reviewer.

Major comment 5) Centrality. The authors compute centrality by comparing Roman roads with modern road networks. However, the manuscript does not clearly define what is meant by ""main modern roads."" It remains unclear whether the analysis includes only motorways or also primary roads. This point should be specified explicitly. Moreover, the authors should justify the restriction to major modern roads when computing centrality. Including a broader set of road types could provide a more representative picture of contemporary accessibility, which may not be fully captured by focusing exclusively on high-capacity roads.

Since the manuscript does not include riverine and maritime connections, We would suggest better clarifying in the conclusions that terrestrial centrality does not necessarily correspond to actual multimodal centrality. The authors should indicate how the network structure and centrality rankings might change with the inclusion of riverine and maritime layers (both ancient and contemporary) and, even if only qualitatively, discuss the extent to which the current findings are driven by the choice of a purely terrestrial network.

The description of the modern road dataset has been elaborated, and arguments have been added why this is an appropriate dataset, and why the comparable coverage between the two datasets enables a meaningful comparison. We have modified the discussion section to more clearly state the results exclusively reflect the structure of terrestrial transport infrastructure. We have highlighted the importance of riverine and maritime connections (especially for the case of Rome, but we equally argue in our results section that the centrality of a list of capital cities should be understood in light of their function as major ports), argued for future work to identify the structure of the multimodal transport network, and have provided a qualitative interpretation of how this would lead to different centrality results.

Major comment 6) Start and end points. The authors state that “Sinuosity can be used as a measure of the “straightness" of the road, calculated as the ratio of actual road length to the straight-line distance between the start and end point (Fig. 1c)." However, the definition of the start and end points is ambiguous. It would be helpful to clarify whether these points correspond to the limits of the entire road or to the endpoints of the 1 km road segments used in the analysis, particularly given the earlier statement that “Results were derived for 1 km stretches of roads to enable comparability." Another point has to do with the comparison between pre-Roman and Roman roads. The authors compare Roman with modern roads finding that “[. . .] major modern roads (Fig. 1d) tend to be straighter than ancient roads [. . .]". While this result is interesting, given that the Romans were known for constructing straight their roads, it would be interesting also comparing Roman with pre-Roman roads.

Start and end points of the sinuosity analysis were derived for 1 km long road segments, the clarification was added to the sentence in question. Comparison with pre-Roman roads was not attempted as we lack appropriate digital dataset of pre-Roman roads that could be used for the comparison. Our discussion now more elaborately emphasises the value of such longitudinal analysis and argues for the need to collect the necessary temporal data. (see also elaborate reply to Major comment 9, which also concerns temporal data)

Major comment 7) Biases and uneven mapping of Roman Roads. An important issue concerns the spatial bias in the mapping of Roman roads. The Itiner-e data paper itself is very explicit that coverage, spatial resolution and source reliability vary substantially across regions due a variety of factors, and it provides representativity, reliability and confidence maps precisely to visualise these heterogeneities. Given this, it would be useful if the authors engaged more directly with the question of which regions are better or worse represented in their analyses, and why. For example, the confidence map highlights large areas of low representativity and/or low reliability in parts of northern Italy (Tuscany, Marche, Po Valley), the Western Balkans, the Upper and Middle Danube, Corsica, Sardinia and Central Anatolia. These are precisely regions where the correlation between Roman and modern roads appears weaker or more heterogeneous in their geography weighted coefficients maps. It would help the analysis if the authors would:

i. Explicitly overlay their main results (correlations and geographically weighted coefficients maps) with the confidence categories from Itiner-e (de Soto et al., 2025);

ii. Discuss to what extent low correlations in some regions might reflect genuine heterogeneity in the coverage of Roman Roads versus data gaps or low-resolution digitisation. The authors should consider using the confidence classes in the maps of the de Soto et al., (2025) paper at least to provide robustness checks that restrict the analysis to polygons with medium/high reliability and average/above average representativity. This would both acknowledge the non-uniform quality of the underlying archaeological record and strengthen the credibility of regional comparisons.

A new dedicated section describing ‘Robustness of spatial regression’ was added to the methods, and summarised in the body text. The representativity and reliability maps from de Soto et al. (2025) were overlaid with the GWR results and a new GWR model for scenario 1 is now applied to high confidence data only. All of this new work

is summarised in Results and elaborately described in 'Robustness of spatial regression', with new Supplementary Figures 6, 7, and Supplementary Table 7.

Major comment 8) Interpreting regional variation in Roman-modern correlations with the new dataset. The manuscript rightly emphasises that the new dataset reveals a more complex picture of the relationship between Roman and modern roads, including regions of Europe with low correlation and parts of the Near East and North Africa where correlation is surprisingly high, contrary to some earlier expectations. The authors conclude that "other variables, likely cultural, institutional, economic and political, are on a regional scale stronger predictor of post-Roman infrastructural and economic developments"" This observation opens an important question that could be developed further: To what extent are these new patterns driven by the improved road data in Itiner-e, and to what extent by differences in the modern road data or in the methodology used in this analysis vs the ones in previous papers?

Since Itiner-e nearly doubles the known road length relative to DARMC and greatly increases spatial precision in several regions (Iberian Peninsula, Aegean, Egypt, etc.), (de Soto et al., 2025) it would be valuable to clarify whether:

i. The regions where they now find higher correlations (e.g. parts of the Near East and North Africa) are precisely those where Itiner-e adds many roads or enhances spatial accuracy.

ii. Conversely, some European regions with low correlation (e.g. Brittany, parts of southern Italy) coincide with areas of low representativity / low reliability in the confidence map.

A simple way to address this would be to show, for some representative regions, how the Roman-modern correlation changes when using DARMC vs Itiner-e, or when restricting the Itiner-e data to high-confidence polygons. This would help the reader to understand whether the new dataset truly changes our substantive understanding of where Roman roads matter most for modern infrastructure, or whether the differences mainly reflect the underlying archaeological/documentary biases or statistical choices. If the authors wish to argue that \other variables"" are locally stronger predictors of modern infrastructure, it would be useful to at least sketch what these could be (e.g. medieval and early-modern state capacity, post-Roman trade routes, religious or political frontiers) and, if possible, include a simple multivariate specification where Roman roads are analysed jointly with a set of such covariates.

A new section 'Robustness of spatial regression' has been added that implements some of the suggestions of the reviewer: the reliability and representativity of the Itiner-e data is visualised in Supplementary Fig. 7 and their impact on the results is more explicitly discussed in Results and Discussion, and Supplementary Fig. 6 visualises the areas/polygons of higher confidence in the Itiner-e data. Results are now more explicitly interpreted at a regional level and the variations in density in the Itiner-e dataset are now discussed in this context. Additional arguments for the comparability of the modern road data with Itiner-e have been added (see also reply to major comment 5). Our significantly elaborated interpretation in the discussion expands on the factors that could be explored for explanatory power in future work.

Major comment 9) Chronology and the military vs non-military purposes of roads.The authors point out that not all Roman roads were built with purely military objectives. The

point on the military needs determining the development of the network has been never questioned by historians, at least for the Consular (main) roads. One of the strengths of the Itiner-e dataset is that, where available, it includes fields on chronology and on the magistrate or emperor under whose tenure the road was formalised (Lower-Date, Upper-Date, Construction Period, see table 2 of De Soto et al.). Although the data paper emphasises that chronological information is incomplete, these attributes still offer an opportunity for at least exploratory differentiation between major phases of road construction. Therefore, the dataset appears well suited to testing whether there are systematic differences between roads created in the Republican period (509-27 BCE) and those formalised in the

Imperial period (27 BCE-ca. 400 CE). By using the information on Roman sites could ask whether:

i. Republican roads are more strongly associated with military expansion and frontier consolidation.

ii. Whereas later Imperial roads serve more clearly administrative, commercial or accessibility functions within already consolidated provinces.

Even if a full empire-wide chronological reconstruction is beyond the scope of the current paper, a limited analysis on the subset of segments with known construction periods, or at least a descriptive discussion of how much of the network can be reliably dated and what spatial patterns emerge, would enrich the argument that Roman roads cannot be reduced to a purely military infrastructure. It would also make the most of the chronological fields that have been included in Itiner-e.

The Itiner-e dataset includes only 1/3 main roads and 2/3 secondary roads. We specify in several places that military objectives were a motivation in main road construction alongside other factors, but that this is less the case for secondary roads.

We agree that a longitudinal analysis or temporal comparison would be highly insightful. Sadly the information to perform this on an empire-wide scale in an empirically informed way is not currently available, and is also not reflected in the itiner-e dataset. Chronological information is highly sparse and mostly available for main roads which form the minority of the network. Even for provinces with the best data at most it allows for showing sequences of selected stretches of major road construction/redevelopment, which is interesting but not the same as an empirically informed temporal development of the provinces road network as a whole. Such an analysis is therefore not feasible in the current paper. We have therefore decided to elaborately mention this in the discussion as an important avenue of future research dependent on data collection/aggregation. Our introduction also emphasises the temporal development of Roman roads based on a pre-Roman substrate. Our paper publishing the Itiner-e dataset (de Soto et al. 2025) discusses this lack of reliable chronological information at this scale, the lack of such information in the current version of the dataset, and the need for future research to expand open data in this direction.

Major comment 10) Cultural features, Pleiades, and the distinction between main and secondary roads.The manuscript briefly refers to “cultural variables"" and acknowledges that

major roads were often linked to military campaigns, but the potential of the data to explore cultural and functional differentiation within the network is not fully exploited. Itiner-e is explicitly linked to Pleiades and other gazetteers of ancient places, which contain rich information on the location of forts, sanctuaries, temples, theatres, baths and thermal complexes, cemeteries, and other features of Roman daily and religious life. Some regions - around major sanctuaries, spa towns, or legionary bases - may show distinctive patterns in how roads branch, converge, or change hierarchy. Itiner-e itself distinguishes between “Main Roads"" and \Secondary Roads"", based on milestones, inclusion in itineraries and continuity with historically known routes (de Soto et all, 2025). A more explicit discussion of this potential - and of how secondary roads might reflect cultural and everyday mobility rather than only military or administrative needs - would help underline the added value of the new dataset.

Using Itiner-e plus Pleiades for some regions, would illustrate how cultural factors shaped differently the layout of both major and minor roads. Two lines of analysis could be hinted at more clearly:

i. Functional differences between main and secondary roads.

ii. If it is true that secondary roads are likely to have been less directly tied to long-distance military campaigns and more to local economic, social and cultural connectivity, correlating the density of secondary roads with the presence of thermal sites, sanctuaries, villas, forts or other specific categories of sites could help to substantiate the claim that Roman roads also served non-military purposes and to show which parts of the network were more closely associated with which types of activities.

We thank the reviewer for this comment, which points to a clear and potentially valuable line of research. We agree that combining Itiner-e with Pleiades-linked categories of places could help examine whether main and secondary roads relate differently to forts, sanctuaries, thermal sites and other features of Roman daily and religious life, and whether some regions show distinctive branching and hierarchy patterns. We have pointed out how our results show differences between main and secondary roads in edge betweenness. However, in this paper we do not have the space to further develop this analysis in a systematic way, and we plan to pursue it in future work. In the meantime, we have expanded the section “Main roads central in provincial road networks” to describe more explicitly how roads are catalogued in Itiner-e, and to discuss (also in the Discussion section) the likely functional differences between road classes and the kinds of mobility they supported within provincial networks.

Minor comment 1) The structure of the paper is difficult to follow and can be better organised. Placing the Materials and Methods section at the end of the manuscript makes it harder for the reader to fully understand the analysis while reading the main text. Key methodological information is often required to interpret the results, yet the reader is forced to repeatedly move back and forth within the paper to retrieve these details. We recommend integrating methodological information into the sections where it is most directly relevant or alternatively repositioning the Materials and Methods section earlier in the manuscript. This would substantially improve readability and allow the reader to better assess the assumptions, data, and techniques at the point where they are discussed.

The style guidelines of Nature Communications require the Results section to precede and be separate from the Materials and Methods, the order of which also cannot be moved. We decided to adhere to the style guidelines of the journal (as requested by the editor). However, we have made significant changes throughout with the aim of making the text and results more readily understandable, with significant expansion in the Method section.

Minor comment 2) The paper contains many interesting ideas and pieces of information scattered throughout the manuscript, but several of them are only briefly mentioned and not sufficiently developed. As a result, the reader is left wanting more explanation and context. While we recognise that these aspects cannot be explored in great detail without substantially increasing the length of the paper, providing some additional discussion would help address otherwise unresolved curiosity. In particular, the following points would benefit from further elaboration:

i. Pre-existing roads used and re-paved by the Romans.

ii. Alpine roads show lower slope values, orientation of roads.

iii. Modern roads are slightly more negatively correlated with elevation and TPI than Roman roads are.

iv. While the Roman roads are globally good predictors of modern road infrastructure there are other variables, likely cultural, institutional, economic and political, that are on a regional scale stronger predictor of post-Roman infrastructural and economic developments.

v. Cultural features like the Roman frontier (Rhine, and Danube to some extent): Are these cultural or geographical features?

We also note similar requests by Reviewers 5 and 6 to elaborate on the historical and substantive contexts. Based on the reviewers' comments, we have sought to clarify several historical and substantive aspects. These additions are intentionally brief so as not to exceed the manuscript's length limits. Specifically, we (i) expand on the existence and regional variability of pre-Roman routes and explain that the role of Roman engineering ranged from upgrading pre-existing roads to constructing new ones, depending on settlement organisation and the politico-military context; (ii) briefly explain why modern roads tend to be straighter and less constrained by topography than Roman roads, given contemporary surveying capabilities and large-scale construction methods; (iii) elaborate on the interpretation of Alpine slope values, highlighting both the presence of relatively favourable passes and valleys and, above all, the strong Roman strategic interest and engineering effort to secure overland connectivity between Italy and transalpine Europe; and (iv) note that on the comparison with modern roads that subsequent historical, political, and economic changes have generated new corridors and communication hubs that can drive departures from Roman-era networks.

Minor comment 3) The authors acknowledge that the network aggregates multiple historical phases. It would be useful to discuss, even qualitatively, which of the results may be sensitive to the lack of temporal differentiation in the network. Clarifying how the aggregation

of different phases might blur regional or temporal variation would help the reader better assess the robustness and interpretation of the findings.

In addition to the more elaborate discussion of the temporal information (see reply to Major comment 9), we have expanded the discussion to clarify the aggregated nature of the dataset affects the temporal interpretation of the results, further emphasising the need to collect temporal information.

Minor comment 4) Regarding the “cultural variables" discussed on page 6, we would not classify ancient sites and modern population as cultural variables. Rather, these measures are more appropriately interpreted as indicators of urbanisation. Reframing them as such would improve conceptual clarity.

"Cultural variables" were changed to "anthropic variables" throughout the article, and it was further specified in the specific context on p. 6 that this means the density of Roman period settlements.

Minor comment 5) An opening parenthesis is never closed in the following passage: “(mainly in Western Europe, the Pannonian basin, North Africa and low lying regions of Italy and Asia Minor (Po valley, Puglia, central Anatolia and Syria)".

This is now corrected.

Minor comment 6) In Figure 1, panel f) is described as a “linear regression of the topographic variables". This wording is misleading. The figure appears instead to present a matrix composed of histograms, Pearson correlation coefficients, and scatterplots. The caption and related text should be revised accordingly to accurately reflect the content of the figure.

The caption was changed to "matrix summarizing the relationship between the topographic variables."

Minor comment 7) The manuscript sets the minimum neighbourhood distance for the GWR kernel at 1000 km because the global Moran's I z-score peaks at the 900 km band. Because z-scores inflate with the large number of cell pairs at long distances, that peak does not by itself indicate the scale at which the substantive autocorrelation is strongest. Standard practice is to select a GWR bandwidth by optimising an information criterion (AICc or cross-validation) and/or by ensuring a minimum neighbour count for numerical stability. Please clarify:

i. whether an adaptive bandwidth subsequently underwent AICc/CV optimisation in ArcGIS, and

ii. what practical consideration (e.g., required minimum number of neighbours per local regression) led to the 1000 km threshold.

Stating these points explicitly would make the bandwidth choice transparent and reassure readers that the local models are neither under- nor over-smoothed.

We are very grateful to the reviewers for this observation and suggestion. Adaptive neighbourhood optimization to find optimal number of neighbours (instead of a

distance band) using the Golden Search method was applied to find the best performance model comparing AICc and adjusted R2. It was found that in case of scenario 1 and 2, the best performing models use smaller neighbourhoods than was used initially, thus revealing that our GWR model oversmoothed the results. Scenario 1 was affected the most, with scenario 2 providing similar results using both 1,000 km distance band and optimal number of neighbours. The results and discussion were updated in the Results and Methods section, including Figure 3 and tables in the Supplementary Information. For completeness and comparison, the supplementary figures and tables now include the originally reported results using 1,000 km neighbourhood as well as optimal number of neighbours.

Minor comment 8) The factor 0.6 in the stated pace equation diverges from the canonical “inverse-Tobler"" value of 1/6 ≈ 0.167. On level ground it implies a speed of only about 1.4 km h^{-1}, far below the walking or cart speeds normally assumed in Roman-period studies. Because the edge weights in the betweenness analysis depend exponentially on slope, the choice of the factor affects which routes appear shortest. Please either

i. confirm that the 0.6 is a typographical error and provide the corrected value, or

ii. supply a citation and brief explanation (e.g., empirical adjustment for cart traffic) that justifies using a slower, non-standard variant of Tobler's function.

This is not a typographic error, but rather an issue that required us to modify the text to specify the units. The text now specifies the physical units: the pace is expressed in seconds per meter. This translates into a speed of around 5 km/h.

Minor comment 9) The stated study-area of 5.27 billion km2 is impossible (it exceeds Earth's surface area) and conflicts with the 5.5 million km2 value cited elsewhere in the paper. Using 5.27 million km2 reproduces the published density of 56.748 m km2, showing that the text contains a typographical order-of-magnitude error. Please correct the area value.

Typographical error in decimal separator was corrected.

Minor comment 10) The six orientation classes are said to be “covering of the compass each," yet the labels given (N-S, NNE-SSW, NE-SW, E-W, SE-NW, SSE-NNW) do not correspond to the centres of six contiguous 30° bins. As written, a reader cannot deduce the exact angular boundaries used to generate Figure 1b. Please specify the numerical limits (or mid-points) of each class so that the orientation analysis can be replicated unambiguously.

A new Supplementary Table 8 showing delimitation of angular boundaries was included and referenced in the caption of fig1.

Minor comment 11) The Discussion treats the lower sinuosity of main roads as noteworthy evidence for “straight Roman roads," whereas earlier sections describe the main-versus-secondary difference as small (“negligible" / “does not change much"). Clarifying the magnitude of the effect in the Discussion – perhaps by noting that it is minor but still the closest Roman analogue to modern straightness - would make the narrative more consistent.

We agree with the need to avoid confusion and ensure the narrative is consistent. We have added the numbers in the discussion to explicitly indicate the difference, and

nuanced the statement in the discussion to indicate it is a slight difference, and we modified the sentence in the method to use the same terminology "slight".

Minor comment 12) We initially found the phrase “density value of 1 where the surface overlaps with the highest values of the segment" ambiguous, since ArcGIS's Kernel Density tool normalises a quartic kernel to a peak of 1 only as an intermediate step before rescaling outputs to line-length-per-area units after all kernels are summed. Clarifying that the final raster values are the standard ArcGIS line-density outputs and that no custom weighting or rescaling was introduced would remove this uncertainty.

In the Methods section, a clarification was added that "no custom weighting or rescaling was applied."

Minor comment 13) The statement that the observed local correlations are “rather accidental" is currently tied to the low global adjusted R2 (0.10). A low global R2 in a GWR does not, by itself, render all local relationships spurious. It would be clearer to attribute the likely spuriousness to the pattern of correlations being confined to large modern cities - where archaeological recovery of Roman roads is better - rather than to the summary fit statistic alone.

The sentences in question were reformulated according to the suggestion.

Minor comment 14) Statement [“previous studies suggested lower correlation"] initially led us to pause, because Dalgaard et al. (2022) describe “weaker persistence" chiefly through a smaller coefficient, whereas the present paper uses local GWR R2 as its correlation measure. After revisiting Dalgaard, however, we see that their camel-belt specification also reports a noticeably lower goodness-of-fit, so summarising their finding as “lower correlation" is therefore defensible. To eliminate any ambiguity for readers who recall only the coefficient result, you might add a brief clause clarifying that both studies refer to explanatory power (model fit), not merely slope magnitude.

We have added a clarification in parentheses to the sentence, and the comparison with previous studies is significantly expanded, see reply to major comment 1.

Minor comment 15) Table S3 lists an adjusted R2 of 0.3482 (unadjusted 0.3662) for Scenario 3, whereas the main text cites 0.39. Please verify which figure is correct and amend either the text or the table so the reported statistics are consistent.

Typographical error was fixed in the Methods section and the value was corrected to 0.35 (rounded).

Minor comment 16) The phrase (Fig. 3a; SI Appendix, Table S3) “a strong relationship but not too significant" conflates two different ideas. R2 indicates goodness-of-fit, while statistical significance would be assessed with an F-test p-value (which, given 2,500+ observations, would be vanishingly small for an R2 of 0.47). Consider rephrasing to convey that the model explains a substantial yet far-from-total share of the variance, without using the term “significant."

The sentence was rephrased.

Minor comment 17) (p.5 paragraph “The straightness of Roman roads . . . ") In the sentence “We observe that sinuosity is slightly positively correlated with maximum slope (Fig. 1f), i.e. growing average slope correlates with higher sinuosity of the roads." \i.e." implies that the correlation with maximum slope is the same statement as the correlation with average slope, even though the variables are distinct. Replacing “i.e." with a connector such as “and similarly" (or separating the findings) would report the two positive correlations without equating them.

"Average" in the sentence was replaced with "maximum", as the analysis and the text deals only with maximum slope.

Reviewer #1 (Remarks on code availability):

The code made available is not replicating the entire analysis summarised in the paper. The weighted regression analysis is produced by a different script.

Moreover, the authors do not made the data available to replicate the analysis. This is a practice that have to be promoted by scientific journals in general and Nature Communications in particular.

A R-project that include all the analysis is advisable. The authors could consider to deliver an R package with appropriate vignettes to instruct how to replicate the paper's content and how to expand the analysis of the original data.

At this stage we cannot guarantee that the script produced is compilable and that the analysis is consistent with the one contained in the paper.

The datasets are available in a dedicated Zenodo repository: Pažout, A. et al. Network Science reveals the structure and lasting impact of the Roman road system: Supplementary datasets. Zenodo https://doi.org/10.5281/ZENODO.18610056 (2025) .

To facilitate reproduction of analysis, all data sets are now also included on the network analysis GitHub repository cited in the text. https://github.com/MatteoMazzamurro/roman-road-networks

The creation of an R package is outside of the scope of this paper.

As also mentioned in minor comments 7 and 12, the GWR was performed in ArcGIS (desktop GUI software, not code-based and therefore not in the script), and the Methods now clearly specify the ArcGIS version and tools used.

Reviewer #3 (Remarks to the Author):

I co-reviewed this manuscript with one of the reviewers who provided the listed reports. This is part of the Nature Communications initiative to facilitate training in peer review and to provide appropriate recognition for Early Career Researchers who co-review manuscripts.

Reviewer #4 (Remarks to the Author):

I co-reviewed this manuscript with one of the reviewers who provided the listed reports. This is part of the Nature Communications initiative to facilitate training in peer review and to provide appropriate recognition for Early Career Researchers who co-review manuscripts.

Reviewer #5 (Remarks to the Author):

This paper presents an ambitious and technically sophisticated analysis of the Roman road system at an unprecedented spatial scale. By combining a newly compiled high-resolution dataset with network analysis, the authors address several long-standing questions concerning the structure of Roman roads, their relationship to modern infrastructure, and the role of major cities within the network. The scope of the paper, the analytical approaches, and the dataset used represent a major achievement and make this work well-suited for Nature Communications.

Overall, the paper makes a valuable contribution to the quantitative study of ancient infrastructure. I appreciated particularly the clarity of the research targets and the structure of the final discussion, which makes the paper easy to understand and read.

I recommend this paper for publication after minor revisions.

Below, I outline a few general points that I believe would help strengthen the manuscript. Please also refer to the comments added directly to the manuscript.

1. The methodological contribution of the paper is very strong; however, the historical and conceptual framing would benefit from clearer articulation. While the authors successfully identify and quantify structural patterns in the road network, the implications of these patterns for Roman history are sometimes left implicit or expressed in rather general terms. The argument would be strengthened by situating the study more explicitly within existing debates on Roman mobility, infrastructure, and imperial organisation.

We thank the reviewer for this constructive suggestion. Reviewers 1 and 6 also recommended strengthening the historical and conceptual framing, and we have made the necessary changes. We have revised the Introduction and discussion to situate the study more explicitly within key debates on Roman mobility, infrastructure, and imperial organisation. In particular, we now foreground questions concerning (i) who designed and coordinated the road network (centralised imperial planning versus provincial and local drivers), (ii) the functions and intended uses of roads (military, administrative, and economic), and (iii) the role of Roman surveyors and engineers in route selection and construction—especially the extent to which Roman roads represent new construction versus the upgrading and formalisation of pre-existing corridors. We have expanded the discussion of several results to make their historical implications more explicit, thereby facilitating interpretation by Roman historians and archaeologists and clarifying how the quantified structural patterns relate to broader arguments about connectivity and territorial organisation in the Roman world.

2. The sections examining correlations between Roman and modern roads are among the most interesting in the paper and illustrate pretty well any bias and data limitations. In some

cases, however, the interpretations appear to imply a stronger causal continuity than the data can strictly support. The discussion would benefit from greater emphasis on intervening historical processes—such as medieval transport systems, early modern state formation, and differing governance strategies across Roman provinces—that may have shaped long-term infrastructural trajectories.

We appreciate the reviewer's point and agree that present-day transport networks reflect multiple historical layers, with intervening processes and regionally variable trajectories across the former Roman Empire. While this article focuses on Roman roads, and on the fact that they contributed to the long-term configuration of mobility, we do not intend to imply a direct or exclusive causal continuity from Roman to modern infrastructures. We have therefore reworded selected sentences (see also replies to reviewer 1) to ensure that no explanatory power or causality is implied, emphasising the exploratory nature of our results. We have further revised the relevant sections by adding phrasing that explicitly acknowledges the multicausal nature of modern network formation, including later medieval and early modern transport systems, state formation, and differences in governance and investment strategies between regions.

3. The analysis of the centrality of major cities within provincial road networks is insightful. I wondered whether this could be complemented by applying a different analytical scale, evaluating city centrality within the Roman road network as a whole. Such a comparison between provincial (local) and empire-wide (global) centrality could reveal whether cities that are central at the provincial level retain their importance at an imperial scale, and if so, why. This would be an interesting aspect to explore.

We understand that this is interesting for completeness, and so we added a new Supplementary Dataset 7 which lists the degree, betweenness and closeness of capital cities at an empire-wide scale and we cite this in the section 'The structural location of major cities on the Empire-wide network'. That section describes the time-weighted betweenness centrality at an empire-wide scale and we describe how key major cities are central at this scale, which is reflected in the supplement. We further added an interpretation of this result to the discussion. We believe this section plus the province-level city metrics is the most appropriate way to describe centrality.

4. I applaud the authors for making both the data and R scripts available online to ensure reproducibility. I note, however, that the GitHub repository containing the code for reproducing the network analysis does not currently include the original dataset. This should be added. In addition, I recommend publishing the dataset via Zenodo using the GitHub release function. Zenodo provides a permanent, citable DOI and offers greater reliability than GitHub for long-term data archiving

The datasets are available in a dedicated Zenodo repository: Pažout, A. et al. Network Science reveals the structure and lasting impact of the Roman road system: Supplementary datasets. Zenodo https://doi.org/10.5281/ZENODO.18610056 (2025) .

To facilitate reproduction of analysis, all data sets are now also included on the network analysis GitHub repository cited in the text. https://github.com/MatteoMazzamurro/roman-road-networks

See also comment by Reviewer #1.

Comment directly in article (PDF) on p. 14: What about doing the same also for the imperial road network?

The provincial aspect is important, but it would be nice to see what are the results with a broader scale of analysis by considering the whole Roman network.

See reply to comment 3 above.

Comment directly in article (PDF) on p. 4: Maybe it is better if you show them as bullet points, as it has a greater visual impact

We have implemented this suggestion.

Comment directly in article (PDF) on p. 8 (1/2): The observed differences in kernel density between the Roman and modern networks may reflect the incomplete nature of the Roman road dataset. Just a sentence about the Roman dataset completeness and limitations could be added here or below in the "Materials and Methods" section.

A sentence evaluating the incompleteness of the Roman road dataset on the results of the density analysis was added to the end of the paragraph.

Comment directly in article (PDF) on p. 8 (2/2): They are not very low. I would say weak negative correlation.

In Figure 3d would be good to add an asterisk to these values having a significant correlation p-value<0.05.

I guess that most of the correlations highlighted are not significant and quite weak

Asterisks were added to statistically significant (p-value < 0.05) relationships in Figure 3, figure caption was updated accordingly.

Comment directly in article (PDF) on p. 12: I think that the cited features should appear in Figure 4.

I know, Figure 4 is already dense, but I think that the major cities and rivers should be labeled in the map. It would help a lot the reader.

We have modified the figure (see next comment) and included for reference selected major cities mentioned in results

Comment directly in article (PDF) on p.13: It is not clear to me the key of the colours.

Does the black represent a linear structure?

The green a distributed structure?

It would be worth adding a legend explaining the colours. Alternatively, you can explain this in the caption.

We have significantly changed this figure and its caption to more directly reflect the results (colours now represent three types of provincial network structure).

Reviewer #5 (Remarks on code availability):

I applaud the authors for making both the data and R scripts available online to ensure reproducibility. I note, however, that the GitHub repository containing the code for reproducing the network analysis does not currently include the original dataset. This should be added. In addition, I recommend publishing the dataset via Zenodo using the GitHub release function. Zenodo provides a permanent, citable DOI and offers greater reliability than GitHub for long-term data archiving

The datasets are available in a dedicated Zenodo repository: Pažout, A. et al. Network Science reveals the structure and lasting impact of the Roman road system: Supplementary datasets. Zenodo https://doi.org/10.5281/ZENODO.18610056 (2025) .

To facilitate reproduction of analysis, all data sets are now also included on the network analysis GitHub repository cited in the text.
https://github.com/MatteoMazzamurro/roman-road-networks

See also comment by Reviewer #1.

Reviewer #6 (Remarks to the Author):

The authors attempt and, in my estimation, succeed at producing the first ever truly quantitative study at scale of the Roman road system. The methods and data are significant and the analyses and their interpretations are appropriate in the context of the scale of the question and the limitations of the evidence. The results dispel many anecdotal myths, such as “Roman roads are straight”, “all roads lead to Rome”, “modern roads follow the route of Roman roads”, as well as adding nuance to other forms of received wisdom, including the idea that modern urbanism and industrial development are correlated if not predicated on previous Roman investment in transport infrastructure. These are important corrective results.

For this study to be taken up and extended by domain specialists, especially those from humanities disciplines, much of the statistical jargon and methods of quantitative analysis would need definition in plain language and to be built out in more detail. The scientific format of the paper, separating definitions from results and from interpretations is a further, if unavoidable, impediment. Additionally, since the methods used here are not novel (even if their application on these data is), the number of interpretive areas not undertaken in this, perhaps initial, paper is noteworthy. While it might be too much to ask for an attempt at “what it all means”, there are many next steps that might be briefly included or at least explored if the authors want their paper to be more than an announcement of analyses conducted. For example, the quantitative analysis leads to interpretive regimes that are bounded by quantitative parameters, but many answers in the historical disciplines are also found in qualitative and even subjective evidence. How might these be included? Next, for a subject that is archaeological in nature, the absence of chronology as a variable is striking. What further research would the authors recommend be undertaking for the development of the network over time to be a subject of study? Indeed, this might be a requirement for additional investigation into the question of whether the development of the Roman road system was strategic or organic?

As humanities research, we would like to thank the reviewer for their valuable and constructive comment that emphasises the importance for clear communication to domain specialists. We have defined key jargon like TPI, sinuosity and edge betweenness in clear language where they are first mentioned, leaving more technical explanations for the Methods section. We agree that more historical context and interpretation is merited (this was also pointed out by reviewers 1 and 5). We have revised the Introduction and discussion to situate the study more explicitly within key debates on Roman mobility, infrastructure, and imperial organisation. We have also expanded our description of future substantive research avenues in the Discussion section.

As the reviewer notes, this article is framed differently from much historical scholarship, in that it prioritises quantitative analyses over qualitative discussion. Moreover, our article structure (Results, Discussion, Methods) follows the guidelines of Nature Communications. We adopt this quantitative approach and structure because the Itiner-e data allows us for the first time at high spatial detail to explore the structure of the Roman imperial road system at the scale of the whole Empire, and this new contribution will be of interest to an audience beyond the Humanities.

We also fully recognise the importance of chronology for historical interpretation, especially for a transport network that developed over more than eight centuries. The issue of temporality was also raised by reviewer 1. Our discussion now more elaborately emphasises the value of such longitudinal analysis and argues for the need to collect the necessary temporal data. We have also foregrounded this temporal development in the introduction. (see also elaborate reply to Major comment 9 by reviewer 1, which also concerns temporal data)

In the end, this is an extremely impressive first analysis of a subject matter that lends itself to such analysis and which, in its absence, has generated many unsubstantiated myths. I recommend its publication, but believe the question if the extension of the results toward the humanistic disciplines in this iteration is best determined by discussion between the authors and editors.

Thank you! It is a pleasure to read such kind words about research we are passionate about that was so many years in the making.

These definitions have now been added.

Reply to reviews 2

We are grateful and delighted that all reviewers and editors accept the changes, with only reviewer 1 suggesting further minor changes. We have made revisions in light of these. We present here a reply in bold to each individual comment. All changes in this second round of reviews are highlighted in the manuscript in orange.

General Assessment
We thank the authors for submitting a revised version of the manuscript that we evaluated individually and discussed collectively. The paper has improved in terms of clarity, positioning within the literature, and methodological transparency compared to the initial submission. We think that the authors-reviewers interaction was positive and productive as far as the paper's content.
The contribution remains primarily descriptive and exploratory, but this is now more clearly acknowledged and explicitly framed as complementary to causal econometric studies. This clarification significantly improves the conceptual coherence of the manuscript and helps align the empirical strategy with the scope of the conclusions. We think that the paper will become a benchmark in this field of study and the minor revisions that we ask the authors to implement are mainly due to the conviction of the important role the paper will play as a reference for future works.

We are very grateful to the reviewers for their deep engagement with our work and their assessment of its importance for the field.

The treatment of uncertainty is now more transparent and methodologically sound. In addition, the discussion of data heterogeneity—both in terms of representativeness and reliability—has been expanded, and the new robustness exercises using smaller spatial grid cells (0.25 degrees) are a useful addition. The reconsideration of the GWR bandwidth and neighborhood specification, now based on a standard AICc optimization criterion with an adaptive neighborhood structure further strengthens the analysis.
The authors have also addressed several technical concerns raised in our previous review, including the order-of-magnitude error in the reported study area, the clarification of sinuosity endpoints, and a more precise description of the modern road dataset.
Overall, the revision represents a substantial improvement over the original version. That said, we think that a small number of methodologically relevant issues remain and should be addressed before the manuscript can be considered for acceptance. At this stage, we recommend minor revisions focused on these remaining points.

Comments
1) Positioning in the literature. While the narrative is now more coherent and the complementary nature of the contribution is clearly articulated, one aspect could still be sharpened. In particular, the paper would benefit from a more explicit distinction between i) findings that confirm previous results, ii) those that refine them, and iii) those that lead to substantively different conclusions.
At present, this differentiation is still largely implicit. For example, on page 18 the authors note that "*This result puts into question the previous finding by Dalgaard et al., where high correlation between Roman and modern roads was postulated for Western Europe and low*

correlation for North Africa and the Near East […]". However, the broader implications of this statement for the existing literature are not fully systematized.
Given the relevance that this paper will play for future research in this field, a brief and explicit summary paragraph (even with the use of a Table of results) would help the readers substantially. This would strengthen the paper's positioning and make its added value more immediately visible.

We have significantly restructured the text in the discussion, such that we now have a direct pairing of one of our results followed by a sentence(s) that explicitly states how this relates to previous research. This includes explicit statements where our results confirm, differ or refine previous work.

2) Uncertainty. The paper still treats uncertainty largely as a robustness dimension rather than integrating it directly into estimation. We think this could be acceptable for an exploratory, high-level Nature Communications paper, but the authors should be careful not to overstate "robustness" where representativity is structurally low. A sharp sentencein the Results or Discussion paragraph clarifying that these are robustness checks, not corrections for measurement error, would prevent overinterpretation.

A sharp sentence has been added at the end of the discussion where the impact of data uncertainty is raised as a concern, including a statement that future work could go beyond this by including uncertainty into estimation.

3) Causality language. The GWR section still uses language such as "influence", "effect", "prediction", "explanatory variable" (for instance, on page 27 the authors states "[…] *the influence of Roman roads on the location of modern roads varies widely*"; "*There is no contiguous region or area where the model shows good prediction*."). Even if intended descriptively, this phrasing can be read as causal by non-technical readers. We recommend systematically replacing: "influence/effect" à "association/relationship"; "predict/prediction" à "fit/local association/ explanatory power".

We have made these changes in the formulation throughout, especially in the 'Spatial Regression' and the 'Robustness of spatial regression' section.

4) Travel-time weighting (Tobler hiking function constant). The manuscript continues to report a factor of 0.6 in the stated pace equation without further explanation or correction. This parameter choice requires clarification. If it is a typo error, the constant should be corrected and the results updated accordingly. If it is intentional, the authors should provide a clear justification and citation for this specification. For example, whether it reflects calibration to carts or pack animals, terrain constraints, or empirical estimates of Roman travel times. In addition, a sensitivity analysis would be appropriate. The authors should demonstrate that the main centrality results (particularly the distinction between primary and secondary roads and the inferred centrality of cities) remain stable under plausible variations of this parameter. Given that the betweenness-based findings are central to the paper's contribution, this issue is not merely technical but substantively important for the manuscript's credibility and acceptance.

We would like to point out that we already addressed this comment in the previous round of reviews (Minor comment 8), in which we clarified that this was not an error and the equation is correct since it is the version that represents pace (and reflects around 5km/h), hence we specified this in our previous revision and added the physical units. However, since the reviewers bring it up again, this may confuse other readers, so for this reason we have replaced our formulation with Tobler's formulation and also cited Tobler's paper. We would like to emphasise that the previous formulation was correct and equal to the current, our results reflect the widely used Tobler's hiking function, and we therefore believe a sensitivity analysis of this parameter is not needed since evaluations of alternative speeds and modes of transport (or alternative functions) lie outside the scope of this paper that describes the structure of the dataset. Instead, we have stated in our discussion that diverse modes/speeds of terrestrial transport are part of future work.

5) Geographical heterogeneity. The paper still presents the results largely as a broad overview of the legacy of Roman roads across a wide geographical context. As noted in point 1, the authors should more clearly distinguish between: (i) genuinely novel results that arise at the geographical level from the use of a more complete dataset; (ii) new evidence that revisits or extends previous findings, such as the discussion related to the evidence in Dalgaard et al. for North Africa; and (iii) results that mainly confirm the existing literature on the persistence of historical infrastructure and its effects on modern outcomes in some geographical areas. Structuring the results section around these three elements—potentially as separate subsections—would significantly improve the clarity of the contribution and help readers understand what is new relative to the existing literature from a geographical standpoint.

This comment is related to the above comment 1, since it concerns the positioning and communication of our results. We have made strategic changes in the discussion to specify how some of our specific results relate to prior work. We do not believe restructuring the results section was the right way to achieve this. Instead, we implemented a major restructuring of the text in the discussion. The pairing of one of our results with an explicit comparison to previous work clarifies where we confirm or differ from previous work. These statements in the discussion explicitly address the geographical heterogeneity of our results.

Minor remark

6) In sentence *"[…] which is complementary to previous work in economic history that focused on identifying causal inference*" on page 3 we would rephrase as "*which is complementary to previous work in economic history and historical economics that focused on identifying causal inference*".

We have implemented this change.
